# Supplementary material for: Measuring geographical disparities in England at the time of COVID-19: results using a composite indicator of population vulnerability
Source: BMJ Open. 2020 Sep 29;10(9):e039749. doi: 10.1136/bmjopen-2020-039749 (PMC7526277; doi:10.1136/bmjopen-2020-039749)
Supplement: Supplementary data [file bmjopen-2020-039749supp001.pdf]

Supplementary file

Table S1: Descriptive Statistics

	Mean	Sd	Min	Max	# Obs
Nurses per 10,000 population	3.05	0.87	1.15	6.4	191
GPs per 10,000 population	5.98	0.88	3.81	9.26	191
NHS Hospital staff per 10,000 population	191.92	236.18	0	1592.15	191
IMD CCG Rank	16242.3	4927.95	2261.74	27645	191
COPD prevalence per 10,000 patients	201.32	66.28	79.52	406.13	191
Cancer prevalence per 10,000 patients	306.04	83.35	67.83	593.81	191
CKD prevalence per 10,000 patients	336.75	114.19	108.96	814.88	191
Diabetes prevalence per 10,000 patients	569.58	107.99	241.18	1090.75	191
Hypertension prevalence per 10,000 patients	1427.8	241.87	738.36	1857.47	191
Cardiovascular disease prevalence per 10,000 patients	137.8	35.94	56.69	216.38	191
Proportion aged 70+ per 10,000 patients	38706	27289	3845	207788	191
Ratio of residential care homes per 10,000 population aged 70+	21.11	7.15	1.52	44.73	191
Indicator of unplanned admissions for chronic disease	924.97	272.18	170.3	1666.9	191
Indicator of hospital readmission within 30 days	14.22	1.09	11.6	17.6	184
Indicator of mortality within 30 days of discharge	403.6	67.64	211.27	573.09	184
Indicator of acute emergency admission	1427.97	376.18	243.4	2305.8	191
Rural/Urban GGC Indicator	4.17	1.57	1	6	191

Figure S1: Residential homes care and population age distribution at CCGs level

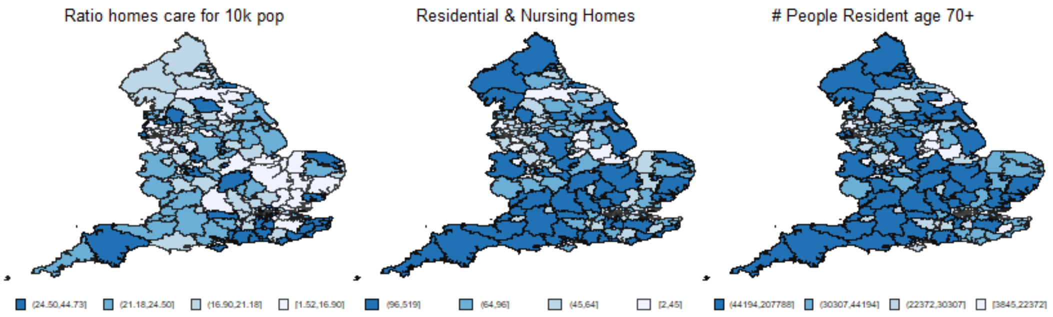

Source: Own elaboration based on NHS digital data at CCGs areas.

Figure S2: Prevalence diseases distribution across CCGs areas

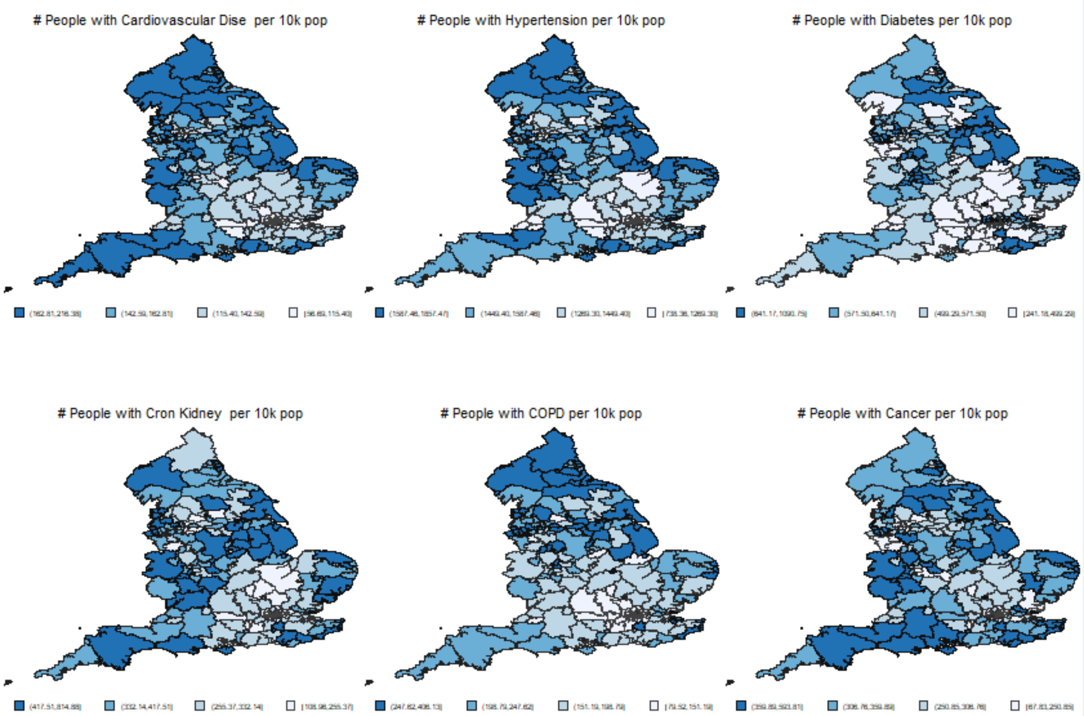

Source: Own elaboration based on NHS digital data at CCGs areas 2019.

Figure S3: Workforce employed by the National Health System at CCGs level

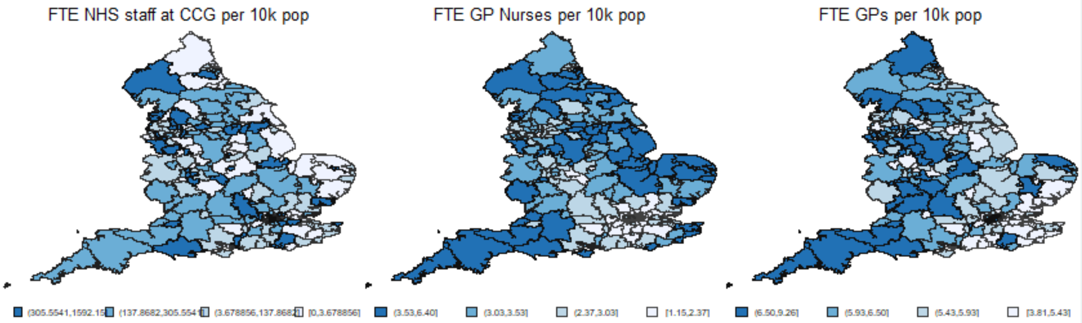

Source: Own elaboration based on NHS digital data at CCGs areas 2019.

Figure S4: Quality of Healthcare Indicators at CCGs level

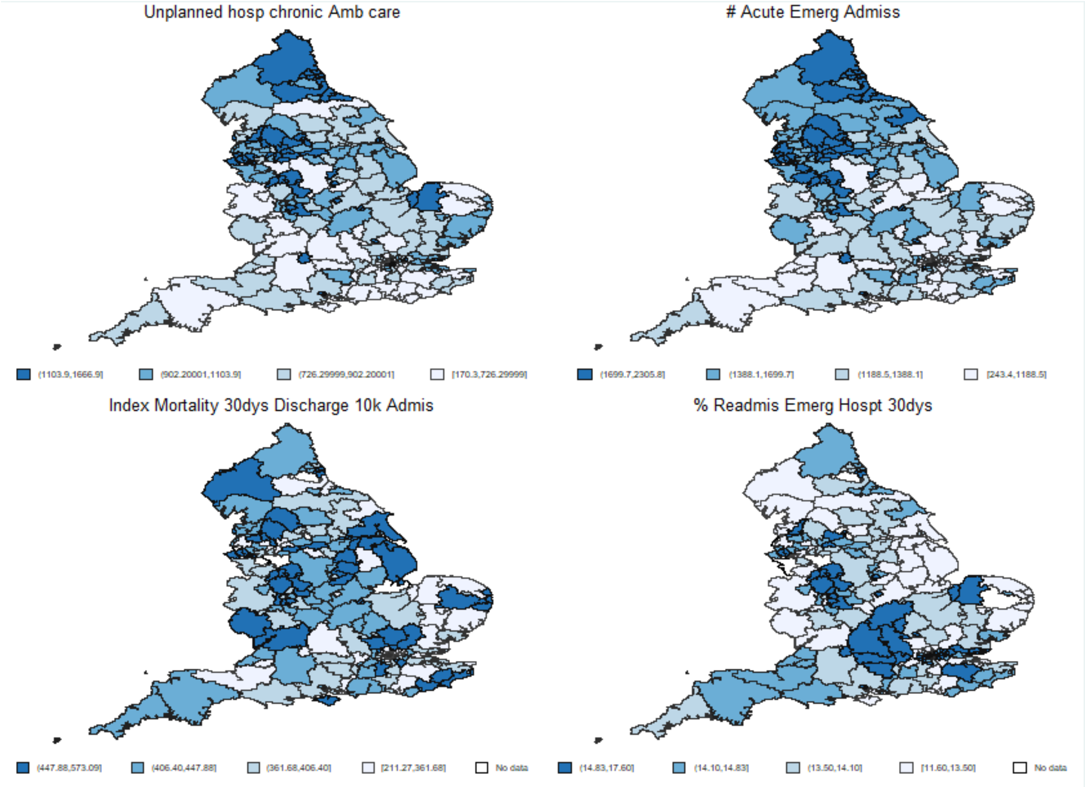

Source: Own elaboration based on NHS digital data at CCGs areas.

Figure S5: **Quartile-based Index of Vulnerability at CCGs level**

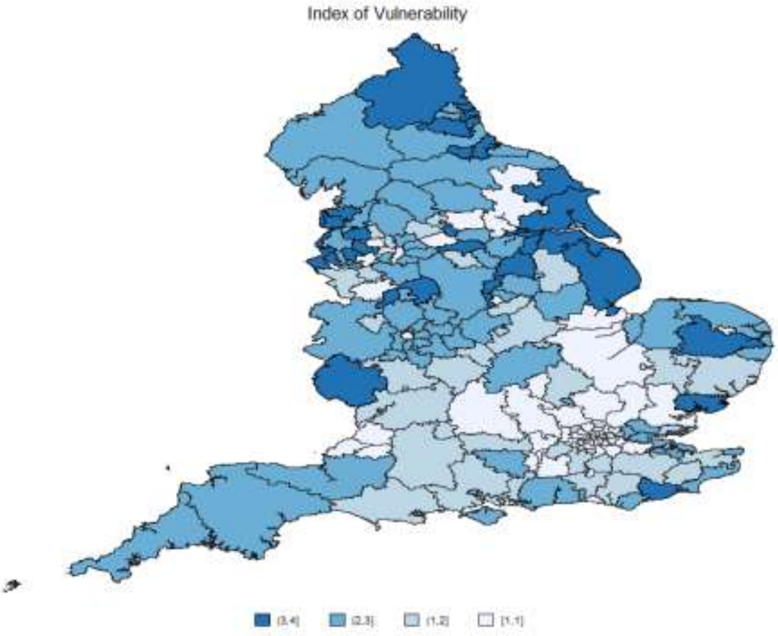

Source: Own elaboration based on NHS digital data at CCGs areas.

Figure S6: **Spatial Cross-Correlation of Index of Vulnerability (left) and IMD (right) with morality**

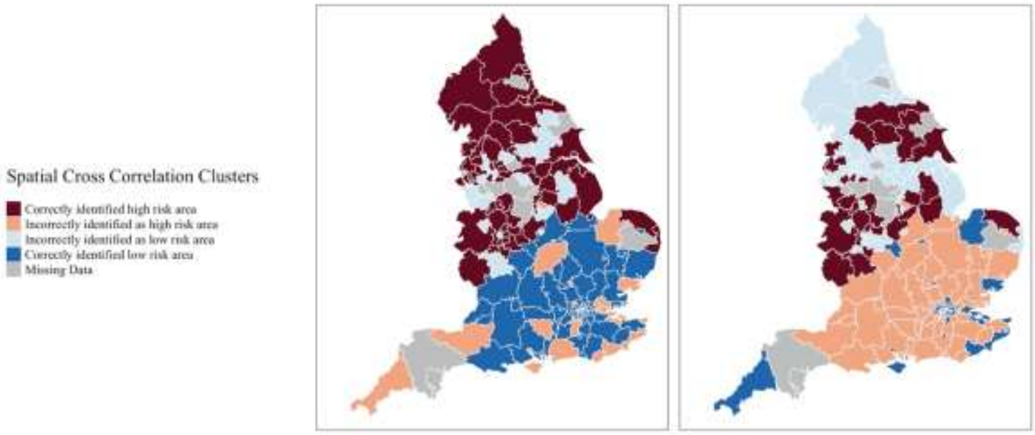

Source: Own elaboration based on NHS digital data at CCGs areas 2019 and mortality rate of the first 22 weeks of the year 2020: computed via the SpatialEco R-package
