# Supplementary material for: Measuring geographical disparities in England at the time of COVID-19: results using a composite indicator of population vulnerability
Source: BMJ Open. 2020 Sep 29;10(9):e039749. doi: 10.1136/bmjopen-2020-039749 (PMC7526277; doi:10.1136/bmjopen-2020-039749)
Supplement: Author's manuscript [file bmjopen-2020-039749.draft_revisions.pdf]

BMJ Open

BMJ Open is committed to open peer review. As part of this commitment we make the peer review history of every article we publish publicly available.

When an article is published we post the peer reviewers' comments and the authors' responses online. We also post the versions of the paper that were used during peer review. These are the versions that the peer review comments apply to.

The versions of the paper that follow are the versions that were submitted during the peer review process. They are not the versions of record or the final published versions. They should not be cited or distributed as the published version of this manuscript.

BMJ Open is an open access journal and the full, final, typeset and author-corrected version of record of the manuscript is available on our site with no access controls, subscription charges or pay-per-view fees (http://bmjopen.bmj.com).

If you have any questions on BMJ Open's open peer review process please email info.bmjopen@bmj.com

BMJ Open

Population Vulnerability to Unexpected Health Shocks: Measuring Geographical Disparities in England at the Time of COVID-19

Journal:	*BMJ Open*
Manuscript ID	bmjopen-2020-039749
Article Type:	Original research
Date Submitted by the Author:	29-Apr-2020

Complete List of Authors:	Nicodemo, Catia; Oxford University, Nuffield Primary Care ; CHSEO, University of Oxford<br>Lasserson, Daniel; University of Birmingham, Institute of Applied Health Research<br>Moscone, F; Brunel University,<br>Shaikh, Mujaheed; Hertie School gGmbH<br>Redding, Stuart; Nuffield Department of Primary Care Health Sciences<br>Barzin, Sammira; Department of Mathematics University of Oxford<br>Cavalli, Nicolo'; University of Oxford Nuffield College

Keywords:	Health policy < HEALTH SERVICES ADMINISTRATION & MANAGEMENT, HEALTH ECONOMICS, Adult intensive & critical care < INTENSIVE & CRITICAL CARE

SCHOLARONE™
Manuscripts

I, the Submitting Author has the right to grant and does grant on behalf of all authors of the Work (as defined in the below author licence), an exclusive licence and/or a non-exclusive licence for contributions from authors who are: i) UK Crown employees; ii) where BMJ has agreed a CC-BY licence shall apply, and/or iii) in accordance with the terms applicable for US Federal Government officers or employees acting as part of their official duties; on a worldwide, perpetual, irrevocable, royalty-free basis to BMJ Publishing Group Ltd ("BMJ") its licensees and where the relevant Journal is co-owned by BMJ to the co-owners of the Journal, to publish the Work in this journal and any other BMJ products and to exploit all rights, as set out in our licence.

The Submitting Author accepts and understands that any supply made under these terms is made by BMJ to the Submitting Author unless you are acting as an employee on behalf of your employer or a postgraduate student of an affiliated institution which is paying any applicable article publishing charge ("APC") for Open Access articles. Where the Submitting Author wishes to make the Work available on an Open Access basis (and intends to pay the relevant APC), the terms of reuse of such Open Access shall be governed by a Creative Commons licence – details of these licences and which Creative Commons licence will apply to this Work are set out in our licence referred to above.

Other than as permitted in any relevant BMJ Author's Self Archiving Policies, I confirm this Work has not been accepted for publication elsewhere, is not being considered for publication elsewhere and does not duplicate material already published. I confirm all authors consent to publication of this Work and authorise the granting of this licence.

For peer review only

Population Vulnerability to Unexpected Health Shocks: Measuring Geographical Disparities in England at the Time of COVID-19

Catia Nicodemo,[1 2 *] Samira Barzin,[3] Daniel S. Lasserson,[4 5] Francesco Moscone,[6 7] Stuart Redding,[1] Mujaheed Shaikh,[8] Nicolò Cavalli [9 10]

[1] Centre for Health Service Economics & Organisation, Nuffield Department of Primary Care Health Sciences, Oxford University, Woodstock Road, Oxford, OX2 6GG United Kingdom
[2] Department of Economics, Oxford University, Manor Road, Oxford, OX1 3UQ United Kingdom
[3] Mathematical Institute, Oxford University, Woodstock Road, Oxford, OX2 6GG United Kingdom
[4] Institute of Applied Health Research, College of Medical and Dental Sciences, University of Birmingham, Birmingham, B15 255 United Kingdom
[5] Department of Acute Medicine, Sandwell and West Birmingham NHS Trust, City Hospital, Dudley Road, Birmingham, United Kingdom
[6] Brunel University London, Kingston Lane, Uxbridge, UB83PH United Kingdom
[7] Università Ca' Foscari Venezia, Dorsoduro 3246, Venezia 30123, Italy
[8] Hertie School, Friedrichstraße 180, Berlin, 30123 Germany
[9] Nuffield College, Oxford University, 1 New Road, Oxford, OX1 1NF United Kingdom
[10] Carlo F. Dondena Centre for Research on Social Dynamics and Public Policy, Bocconi University, 1 Via Guglielmo Röntgen, Milan, 20136 Italy

*Corresponding author: catia.nicodemo@gmail.com, Centre for Health Service Economics and Organisation, Nuffield Department of Primary Care Health Sciences, University of Oxford, Gibson Building, Radcliffe Observatory Quarter, Woodstock Road, Oxford OX2 6GG, Tel+447775158584

For peer review only - http://bmjopen.bmj.com/site/about/guidelines.xhtml

Abstract

Objectives – The growth of COVID-19 infections in England raises questions about system vulnerability. Several factors that vary across geographies, such as age, existing disease prevalence, medical resource availability, and deprivation, can trigger adverse effects on the National Health System during a pandemic. In this paper we present data on these factors and create an index to show which areas are most at risk and to help policy makers moderate the impact of pandemics.

Design – We combine several sources of data to capture vulnerabilities to respiratory pathogen outbreaks on the demand-side and on the supply-side of the healthcare system. We merge these measures to generate an index of community-level vulnerability.

Setting – 191 CCG areas in England.

Main outcome measures – Prevalence rates of high-risk diseases; proxies for the at-risk population density; availability of staff and quality of healthcare facilities. We merge these measures to generate an index of community-level vulnerability.

Results – On the demand-side, we find that the North and South West of England are among the most vulnerable areas. These areas, together with the East Coast areas of Lancashire, also appear vulnerable to adverse shocks to healthcare supply. When turning to the synthetic index, we find that the most vulnerable areas are in the East of England, with the lowest areas for vulnerability corresponding to London, even though this area was hit severely at the start of the COVID-19 pandemic.

Conclusions – Evidence-based policy is crucial to mitigate the negative health impact of pandemics such as COVID-19. While current attention focuses on curbing rates of contagion, we introduce a heuristic tool that can help policy makers to identify the most vulnerable communities. Importantly, we find that this index is correlated to area-level indices of economic deprivation.

Keywords: Geographical disparity; Pandemic; Emergency preparedness; Index of vulnerability; Health inequalities; Covid-19.

Word count: 2948

Strengths and limitations of this study

- We provide important information to help identify the communities most vulnerable to harmful effects of COVID-19
- This fills an important gap in the literature, with only a handful of previous studies that show the distribution of underlying risks within national systems
- Judgement was required when deciding which variables to include or omit
- Our methods give equal weight to each variable when creating the index
- CCGs are quite large and using smaller areas may be more appropriate

For peer review only - http://bmjopen.bmj.com/site/about/guidelines.xhtml

1 Introduction

The current COVID-19 outbreak is triggering significant questions over the distribution of vulnerabilities across different social groups.[1] Over the past decades, evidence accumulated from recurring viral outbreaks such as SARS, Swine Flu, MERS and now COVID-19, indicates infections requiring critical care, and associated case fatalities, are usually, but not exclusively, concentrated among older adults and in patients suffering from specific health conditions, such as chronic obstructive pulmonary disease, cardiovascular diseases, diabetes, cancer, and chronic kidney disease.[2-4] Socially deprived populations have also been found to display generally lower levels of healthcare access and higher levels of infections and mortality in both low middle-income and high-income countries during pandemics,[5] including in England during the 1918 Spanish flu and during the 2009 N1H1 outbreak.[6,7] Previous and current viral pandemics further suggest that respiratory pathogen outbreaks may lead to adverse healthcare supply shocks, as a function of the increased risk of transmission to healthcare workers and of the physical and mental impact of sudden surges in workloads that come with high case fatality conditions.[8] Correctly identifying existing inequalities in vulnerability to viral pandemics can produce critical information to shape effective public policies and help informing a fair and efficient allocation of the stretched resources of national health systems among patients and geographies.[9] Currently, however, epidemic intelligence lacks appreciation of the role of community-level variables in moderating the outcomes of viral outbreaks, with only a handful of studies focused on reconstructing the distribution of underlying risks within national systems.[10,11] To address this gap, we build an indicator of vulnerability for 191 CCGs areas in England by combining information on the prevalence of at-risk individuals within a local community and on the available healthcare supply in that same locality. Consulting this indicator would allow policymakers and healthcare managers to understand which areas might require strengthened surveillance and active monitoring so as to minimise the negative outcomes of unexpected health shocks.

2 Methods

We combine several sources of data gathered from NHS Digital. We present all data at the level of the Clinical Commissioning Groups (CCG, *n*=191) for England, with the 2018/2019 financial year as our main reference, except when explicitly mentioned otherwise. We assess potential vulnerabilities stemming from both the demand- and the supply-side. To assess demand-side vulnerabilities, we consider i) the number of people above 70 years old per 10,000 residents across CCGs; ii) the CCG-area prevalence of specific diseases, collected through the Quality and Outcomes Framework (QOF). In particular, we use the local prevalence of cardiovascular diseases (atrial fibrillation, coronary heart disease, cardiovascular, stroke, heart failure, heart failure due to left ventricular systolic dysfunction), chronic obstructive pulmonary disease (COPD), cancer, chronic kidney disease (for patients aged 18 or older) (CKD), hypertension, and diabetes (patients aged 17 or older). A QOF prevalence is given by the total number of patients recorded in the General Practice register with a certain health condition at a specific point in time, divided by the total number of patients registered within the same practice at that same time. We chose to focus on these diseases in order to match the groups of patients

For peer review only - http://bmjopen.bmj.com/site/about/guidelines.xhtml

considered specifically at risk by Public Health England.[12] Finally, we include iii) the ratio of residential and nursing homes per 10,000 population aged 70 years or over. This indicator allows us to capture the density of older people with high levels of frailty.

To measure potential supply-side vulnerabilities, we map the English National Health System's (NHS) workforce working within each CCG, and a set of indicators that proxy for the quality of the healthcare supply. To measure the size of the workforce, we consider the number of full-time equivalent (FTE) nurses within general practices, general practitioners (GPs) and hospital staff, including non-medical personnel. To consider the supply in each CCG, we use FTE equivalents rather than raw headcounts, as FTEs are a better measure of workforce availability, especially in more deprived and rural areas, where more GPs work part-time. We standardised these values by the CCG-area population. GPs and practice nurses are measured directly at the CCG level. However, NHS hospital staff are measured at NHS Hospital Trust level and CCG level: to harmonise this indicator with the other supply-side indicators, we use the provider code, and the postcode to link the NHS Trusts to the CCG area. Drawing from existing literature,[13] we measure quality by considering i) the percentage of emergency hospital admissions occurring within 30 days of the most recent discharge at the Local Authority (LA) level; ii) the ratio of deaths within 30 days of a non-elective hospital procedure at the Local Authority level (last data available are at 2014, after which NHS Digital has constructed a new indicator that considers total mortality at hospital level). We use a lookup between LAs and CCGs areas to aggregate these indicators at the CCG-level. 7 CCGs do not map correctly with LAs. On the supply-side we also use: iii) the prevalence of unplanned admissions for ambulatory care sensitive conditions at CCG level; and iv) emergency admissions for acute conditions that should not usually require hospital admission at CCG level. These two latter measures are typically used to capture acute hospitalisations in people with specific long-term conditions where optimal primary care delivery can prevent deterioration of those conditions.[14] All supply indicators are indirectly standardised rates based on age and sex specific rates in England.

We combine demand- and supply-side indicators to generate a synthetic index of area-level vulnerability, in order to identify communities which are most vulnerable to the negative health outcomes that could be caused by a severe respiratory pathogen outbreak. In order to build this index, we first create a dummy variable for each area using the England national mean of the indicators presented above. In particular, on the supply side, we assign value "one" to CCGs with lower-than-average healthcare personnel FTE, and lower than average hospital quality - while we assigned value "zero" for CCGs whose values are above the national average. On the demand side, we assign value one to higher-than-average prevalence of underlying diseases associated with higher risk of poor clinical outcomes and to a lower-than-average ratio of residential care home facilities - zero otherwise. Building on OECD guidelines, we obtain a synthetic vulnerability index as the arithmetic sum of these dummies for each CCG area.[15] Therefore, our index represents a functional transformation that, for each CCG, maps the vector of dichotomous indicators into an integer number (*min*=0; *max*=15, *mean*=7.38). Above-or-below mean indices are among a class of additive aggregation methods considered to be robust to outliers. However, they suffer from a substantial loss of information on the relative distance of each CCG compared to the national average. In the Figure 1a in Appendix, we address this issue

For peer review only - http://bmjopen.bmj.com/site/about/guidelines.xhtml

by presenting a quartile-based indicator, which offers similar results to the above-or-below average index.

Patient and Public Involvement – patients and the public were not involved in this research. We would expect that the information provided here can help encourage public debate when considering what other variables may deserve inclusion in similar indices in the future.

3 Results

3.1 Demand side

In Figure 1, we report on the prevalence of residential and nursing homes, the resident population above 70 years old and the ratio of residential care homes across the older-age population. Figure 1 shows that residential and nursing facilities are concentrated in the North and the South West of England, which are also relatively more populated with older people. However, looking at the ratio of homecare facilities over the older population, we observe that areas in the North and East Coast have the lowest ratios. There is evidence that a good system of long-term care could reduce hospital admissions and mortality, especially formal care.[16] However, during a viral pandemic, a higher density of residential home care facilities can represent a risk factor given the frailty of their residents.[17]

Figure 1 HERE

Figure 2 depicts the CCG-level distribution of population prevalence for six diseases chosen with the aim of assessing the overall susceptibility of the system to healthcare demand shocks from the spread of SARS-CoV-2 infections. As mentioned in the Methods section, we consider: Cardiovascular diseases; Hypertension; Diabetes; Chronic kidney disease; COPD; Cancer. We find that the areas with the largest prevalence of these underlying diseases are again located in the North and South West of the country, especially cardiovascular diseases, hypertension and chronic kidney disease. CCGs located in the East of the country, including the East Midlands as well as the Yorkshire and Humber areas are particularly affected by diabetes, COPD and cancer, implying a higher underlying health risk for the population living in these areas, compared to the English average. On the other hand, as Figures 1 and 2 show, the area located around London has fewer older residents and a lower prevalence of high-risk disease types.

For peer review only - http://bmjopen.bmj.com/site/about/guidelines.xhtml

Figure 2 HERE

3.2 Supply side

Potential increases in the demand for critical care driven by the prevalence of underlying comorbidities raise the question of whether the existing community-level supply of healthcare is adequate or whether targeted capacity building should be considered. In Figure 3, we report on the availability of GPs, nurses in general practices and medical personnel in hospitals. We can observe that the healthcare workers are not equally spatially distributed across different areas in England, GPs are more concentrated in the North, the Midlands, and the South West. GP Nurses are spread more evenly across the country, and levels of hospital staff are higher in the South and South East, with the lowest values observed in the coastal areas of the East Midlands and the North East of England.

Figure 3 HERE

Finally, in Figure 4 we plot four indicators of hospital quality: i) unplanned hospitalisations for ambulatory care sensitive conditions; ii) unplanned acute condition admissions; iii) emergency admissions to any hospital in England, occurring within 30 days of the most recent discharge from hospital; and iv) deaths occurring within 30 days of a non-elective hospital procedure. High values of these indicators suggest that the quality of hospital care is low, and that primary care may not be well managed. We show that there is high variation in these quality markers across England. The North East and North West region of the country (North Kirklees and South Sefton CCGs, for example), report a high number of unplanned hospitalisations, acute emergency admissions and a higher rate of deaths after discharge. High mortality rates appear to be spread more widely, with high levels not only in the North but also in the South (e.g. Sussex and Essex CCGs), and especially in the areas of the South West (Devon CCG). In Table 1 (Appendix) we report the descriptive statistics of all indicators used in this study.

Figure 4 HERE

For peer review only

For peer review only - http://bmjopen.bmj.com/site/about/guidelines.xhtml

3.3 A Synthetic Index of Vulnerability

In Figure 5, we plot the synthetic vulnerability index described in the Methods section. According to this index, CCGs in the North and the South of England appear most at risk to the consequences of an outbreak of a respiratory pathogen, with Sunderland, Lincolnshire East, Norfolk, and Borough, among the most vulnerable. (see Table 2 in the appendix for a list of all CCGs by vulnerability score). To test whether CCGs with similar vulnerability levels are more spatially clustered than expected by chance, we computed Moran's I statistic, one of the most widely adopted tests of spatial correlation between observations. Moran's I can be seen as a correlation index that compares statistical units by weighting each pair by a distance function specified by the means of a spatial weight matrix. We have adopted the contiguity criterium to build the spatial weights matrix and standardized it so that the sum of the elements for each row is unity. Results show that Moran's I,[18] calculated on our vulnerability index, is highly significant, indicating positive spatial correlation (I=0.155, with a p-value of 0.00). This implies that geographically contiguous CCGs tend to show similarly high levels of vulnerability.

Figure 5 HERE

When defining the geographical distribution of health vulnerabilities, it is important to consider the potential role of underlying, area-level socio-economic factors as the resources available to tackle a viral outbreak are likely to depend on the level of affluence in the local area. Deprivation negatively interacts with both demand- and supply-side vulnerabilities, as it is found to be positively associated with the timing of the onset of multimorbidities and with the prevalence of long-term conditions.[19] On the supply-side, deprived areas also display higher usage of emergency care relative to elective care, 30% of which is not explained by the different case-mix of conditions suffered by patients from those areas.[20] In addition, recruitment of clinicians to areas of deprivation is challenging.[21] In Figure 7, we plot our vulnerability index against the Index of Multiple Deprivation rank (IMD). IMD is widely used by UK Government departments and previous literature to track relative deprivation in England across seven "domains" (income, employment, education, health, crime, barriers to housing and services, living environment).[22] We show that there is a correlation between this index of deprivation and our vulnerability index. For example, the North East of England appears to have high levels of vulnerability and also poverty.

Figure 6 HERE

4 Discussion

In this article, we have presented a set of indicators to map the vulnerability of the English healthcare system to the unexpected and combined consequences of demand- and supply-shocks associated with respiratory pathogen pandemics such as COVID-19. Before discussing the implications of this work for our understanding of local-area vulnerabilities

For peer review only - http://bmjopen.bmj.com/site/about/guidelines.xhtml

and for future public health action in England, we should first consider the limitations of our analysis. The main limitation of this work stems from the selection of indicators to track supply and demand vulnerabilities, and the connected possibility of omitting relevant variables. For example, we have not considered how potential shortages in the availability of beds can generate supply-side pressures that could influence the healthcare response to a viral pandemic.[12] However, responses to the COVID-19 crisis suggest that the elasticity in the supply of beds is higher than that of the supply of workforce. In fact, while both bed and healthcare personnel can be increased during a crisis, solutions that could feasibly increase the latter (such as speeding up the promotion of trainees into the labour force or recalling retired physicians) have suboptimal implications in terms of quality and organisational efficiency. Moreover, the health workforce is more exposed to infections that could create staff shortages.

Despite these limitations, by generating a synthetic index we show vulnerable communities, with the choice of the variables included to generate our indicator reflecting the sources of these vulnerabilities. In England today, cases of COVID-19 infections are rapidly increasing. Currently, the distribution of recorded cases and fatalities is concentrated in areas with a less frail population and with a better-quality healthcare, such as London and the South East. However, as the outbreak is set to progress, more vulnerable areas in the East, North and South West of the country face considerable risks that will endanger health. Comprehensive evidence regarding the distribution of these 'vulnerabilities', and their sources, can guide policy makers in allocating limited resources across areas by engaging directly with the local communities and health managers. Specifically, community-level knowledge will aid in choosing the right guiding principle - for example, population demographics and illness prevalence within a locality might help; when deciding whether to maximize benefits/prognosis; to give priority to the worst off; or to reward value to the community. Accurate data collection and dynamic information-sharing mechanisms between CCGs - not only of diagnosed cases, but also of staff, medical supplies and excess capacity - will help spread the disease burden and prevent the entire system from collapsing. Our results can help to identify vulnerable communities a) that would benefit particularly from early testing; and b) in which isolation of vulnerable population groups would be particularly beneficial.

Over the past weeks, public health authorities in the country have engaged in a significant capacity-building effort, including opening three temporary hospitals in East London, Birmingham and Manchester. Policymakers should be prepared to deal with pandemics in the most effective manner even in the face of difficult choices. Regardless of whether the stage of community level spread has been reached, CCGs should be prepared at least in accordance with WHO guidelines.[23] As a minimum, this includes a coordinated strategy across hospitals and communities, and between hospitals and healthcare staff within the community. Planning and stocking medical equipment and drug supplies that are necessary to treat not only COVID-19 patients, but also those with other predisposing illnesses at the local community level is crucial. Evidence from previous epidemics, such as Malaria, warns that funding during emergencies tend to be provided in line with health and economic need, but biased towards richer areas.[24] In light of the observed correlation between CCG-level vulnerability and deprivation, it is therefore crucial to pay attention to

For peer review only - http://bmjopen.bmj.com/site/about/guidelines.xhtml

how resources are distributed in times of emergencies. These guidelines for 'fair allocation of resources' should be developed based on direct knowledge about local communities - not only considering the health impact of such choices, but also long-run economic and human capital impacts for the community.

Contributorship: CN and NC developed the original concept, conducted the analysis and prepared the article draft. SB and FM contributed to the empirical analysis and to drafting the article. DSL, MS and SR contributed to the concept of the paper and to writing the article. CN, SR and MS led the data collection. CN, SR and NC led the literature review (research in context).

Declaration of interests: No competing interests. All authors have completed the ICMJE uniform disclosure form at www.icmje.org/coi_disclosure.pdf and declare: no support from any organisation for the submitted work; no financial relationships with any organisations that might have an interest in the submitted work in the previous three years; no other relationships or activities that could appear to have influenced the submitted work.

Funding: This study was not directly funded. CN and DSL are supported by the National Institute for Health Research (NIHR) Applied Research Collaboration (ARC) West Midlands. The views expressed are those of the authors and not necessarily those of the NIHR or Department of Health and Social Care.

Data sharing agreement: All data used in this project is freely available from NHS Digital.

For peer review only

References

1. The Lancet. Redefining vulnerability in the era of COVID-19. *Lancet*. 2020; *395*(10230): P1089. doi: 10.1016/S0140-6736(20)30757-1.

2. Lau EH, Hsiung CA, Cowling BJ *et al.* A comparative epidemiologic analysis of SARS in Hong Kong, Beijing and Taiwan. *BMC Infect. Dis*. 2010; *10*(50). doi: 10.1186/1471-2334-10-50

3. Rivers CM, Majumder MS, Lofgren ET. Risks of Death and Severe Disease in Patients with Middle East Respiratory Syndrome Coronavirus, 2012-2015. *Am J. Epidemiol*. 2016 Sep 15; *184*(6): 460-4. doi: 10.1093/aje/kww013.

4. Zhou F, Yu T, Du R *et al.* Clinical course and risk factors for mortality of adult inpatients with COVID-19 in Wuhan, China: a retrospective cohort study. *Lancet*. 2020; *6736*(20).1-9. doi: 10.1016/S0140-6736(20)30566

5. Tricco AC, Lillie E, Soobiah C, Perrier L, Straus SE. Impact of H1N1 on socially disadvantaged populations: Systematic review. *PLoS ONE*. 2012; *7*(6): e39437. doi: 10.1371/journal.pone.0039437

6. Pearce DC, Pallaghy PK, McCaw JM, McVernon J, Mathews JD. Understanding mortality in the 1918-1919 influenza pandemic in England and Wales. *Influenza Other Respir Viruses*. 2011; *5*(2): 89–98. doi:10.1111/j.1750-2659.2010.00186.x

7. Rutter PD, Mytton OT, Mak M, Donaldson LJ. Socio-economic disparities in mortality due to pandemic influenza in England. *Int J Public Health*. 2012; *57*(4): 745–750. doi:10.1007/s00038-012-0337-1

8. Nacoti M, Ciocca A, Meng AG, Brambillasca P, Lussana F, Pisano M, *et al*. At the Epicenter of the Covid-19 Pandemic and Humanitarian Crises in Italy: Changing Perspectives on Preparation and Mitigation. *N. Engl. J. Med.* 2013; *1*(2). doi: 10.1056/CAT.20.0080

9. Morens D, Folkers G., Fauci A. The challenge of emerging and re-emerging infectious diseases. *Nature*. 2004; *430*: 242–249. doi: 10.1038/nature02759

10. Hayward AC, Fragaszy EB, Bermingham A, Wang L, Copas A, Edmunds WJ *et al.* Comparative community burden and severity of seasonal and pandemic influenza: results of the Flu Watch cohort study. *Lancet Respir Med*. 2014; *2*(6): 445-454. doi: 10.1016/S2213-2600(14)70034-7

11. Verhagen M, Brazel D, Dowd JB, Kashnitsky I, Mills M. Mapping hospital demand: demographics, spatial variation, and the risk of "hospital deserts" during COVID-19 in England and Wales. *medRxiv*. 2020. doi: 10.31219/osf.io/g8s96

For peer review only - http://bmjopen.bmj.com/site/about/guidelines.xhtml

12. Public Health England [Internet]. Guidance on social distancing for everyone in the UK. Updated March 30, 2020. Available from: https://www.gov.uk/government/publications/covid-19-guidance-on-social-distancing-and-for-vulnerable-people/guidance-on-social-distancing-for-everyone-in-the-uk-and-protecting-older-people-and-vulnerable-adults

13. Gutacker N, Siciliani L, Moscelli G, Gravelle H. Choice of hospital: which type of quality matters?. *J Health Econ*. 2016; *50*:230-246. doi: 10.1016/j.jhealeco.2016.08.001

14. Sundmacher L, Fischbach D, Schuttig W, Naumann C, Augustin U, Faisst C. Which hospitalisations are ambulatory care-sensitive, to what degree, and how could the rates be reduced? Results of a group consensus study in Germany. *Health Policy*. 2015; *119*(11): 1415–1423. doi: 10.1016/j.healthpol.2015.08.007

15. Organisation for Economic Co-operation and Development. Handbook on Constructing Composite Indicators. OECD Publishing; 2008, 158 p. Available from: https://www.oecd.org/sdd/42495745.pdf

16. Barker I, Steventon A, Deeny SR. Association between continuity of care in general practice and hospital admissions for ambulatory care sensitive conditions: cross sectional study of routinely collected, person level data. *BMJ. 2017; 356*:j84. doi: 10.1136/bmj.j84

17. McMichael TM. COVID-19 in a long-term care facility - King County, Washington, February 27–March 9, 2020. MMWR. Morbidity and Mortality Weekly Report 69 (2020). Available from: https://www.cdc.gov/mmwr/volumes/69/wr/mm6912e1.htm

18. Moran PAP. Notes on Continuous Stochastic Phenomena. *Biometrika.* 1950; *37*(1): 17–23.

19. Barnett K, Mercer SW, Norbury M, Watt G, Wyke S, Guthrie B. Epidemiology of multi-morbidity and implications for health care, research and medical education: a cross-sectional study. *Lancet.* 2012; *380*: 37-43. doi: 10.1016/S0140- 6736(12)60240-2

20. McCormick B, Hill PS, Redding S. Comparative morbidities and the share of emergencies in hospital admissions in deprived areas: a method and evidence from English administrative data. *BMJ open*. 2018; *8*(8) e022573. doi: bmjopen-2018-022573

21. Gavin M, Esmail A. Solving the recruitment crisis in UK general practice: time to consider physician assistants?. *Soc Policy Adm.* 2002*; 36*(1): 76-89.

22. Ministry of Housing, Community & Local Government. The English Indices of Deprivation 2019. Available from: https://assets.publishing.service.gov.uk/government/uploads/system/uploads/attachment_data/file/853811/IoD2019_FAQ_v4.pdf

23. World Health Organisation. Responding to community spread of COVID-19. Interim guidance. Available from: https://www.dropbox.com/home/covid_uk/literature?preview=WHO-COVID-19-Community_Transmission-2020.1-eng.pdf

For peer review only - http://bmjopen.bmj.com/site/about/guidelines.xhtml

24. Barrenho E, Miraldo M, Shaikh M, Atun R. Vertical and horizontal equity of funding for malaria control: a global multisource funding analysis for 2006–2010. *BMJ Global Health*. 2017; 2:e000496. doi: 10.1136/bmjgh-2017-000496

For peer review only

For peer review only - http://bmjopen.bmj.com/site/about/guidelines.xhtml

Figure 1: Residential homes care and population age distribution at CCGs level

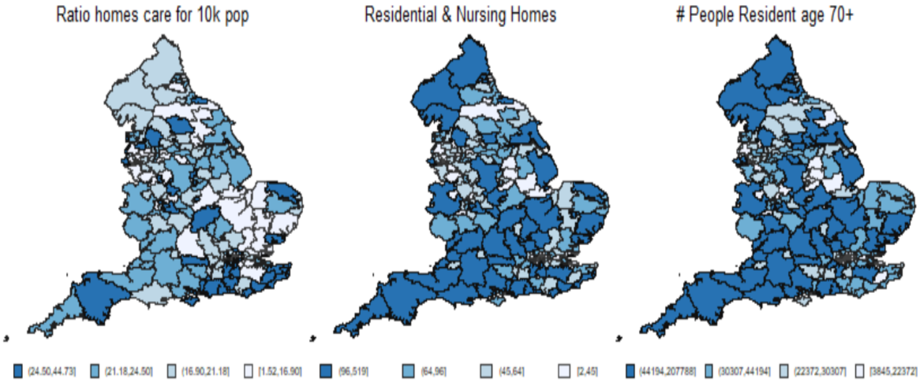

Source: Own elaboration based on NHS digital data at CCGs areas 2019.

Figure 2 Prevalence diseases distribution across CCGs areas

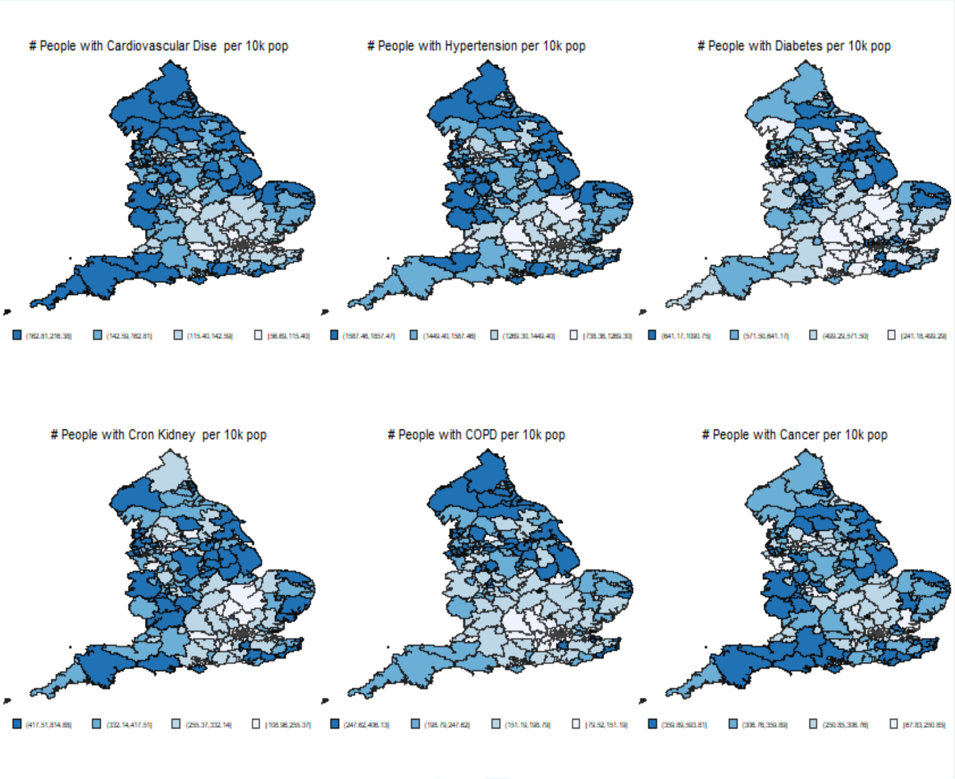

Source: Own elaboration based on NHS digital data at CCGs areas 2019.

eview only

Figure 3. NHS staff distribution at CCGs level

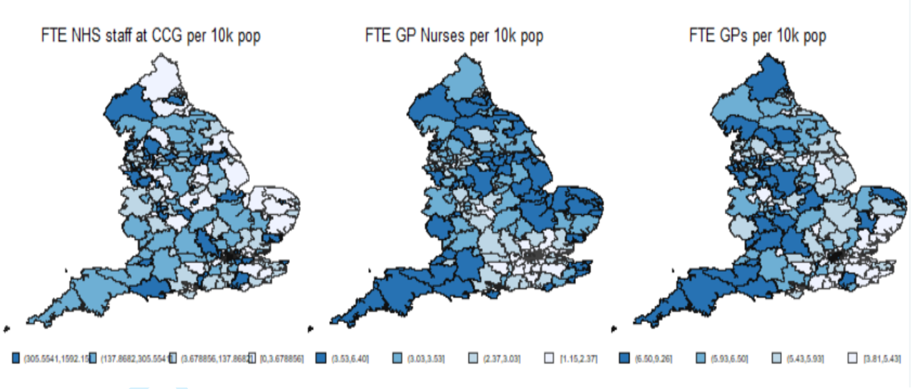

Source: Own elaboration based on NHS digital data at CCGs areas 2019.

Figure 4: Quality of Healthcare

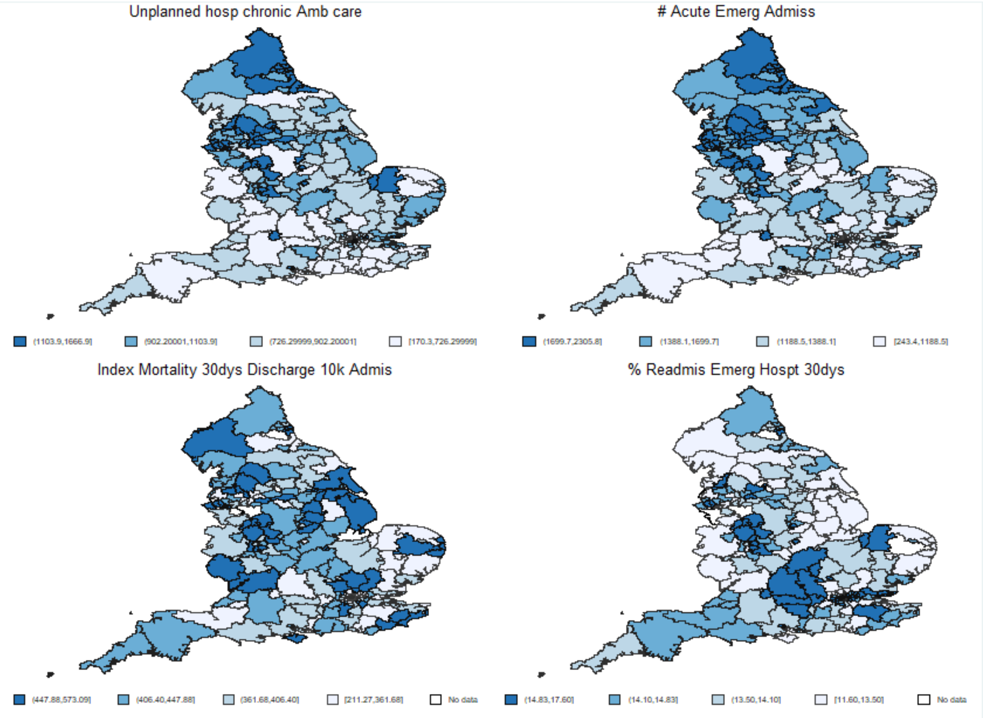

Source: Own elaboration based on NHS digital data at CCGs areas 2019.

Figure 5: Synthetic Indicator of Vulnerability at CCGs level

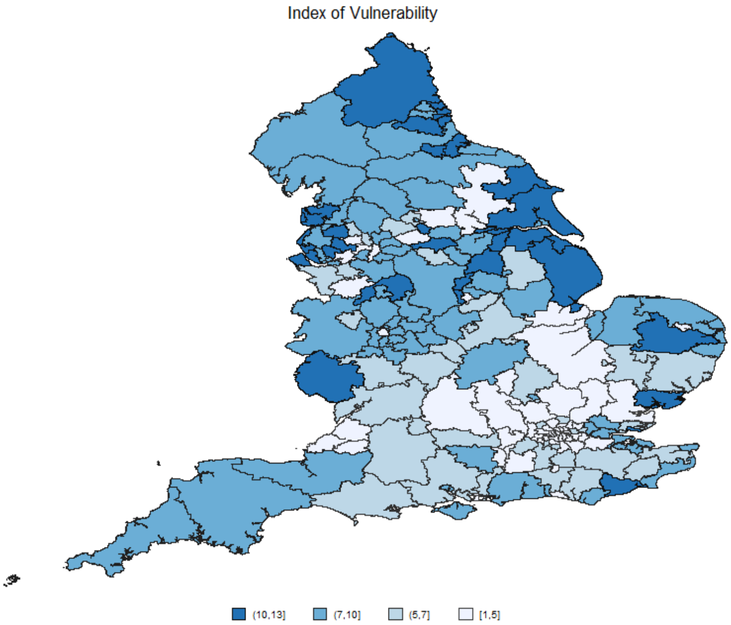

Source: Own elaboration based on NHS digital data at CCGs areas 2019.

For peer review only

Figure 6: Correlation between Indicator of Vulnerability and IMD Rank

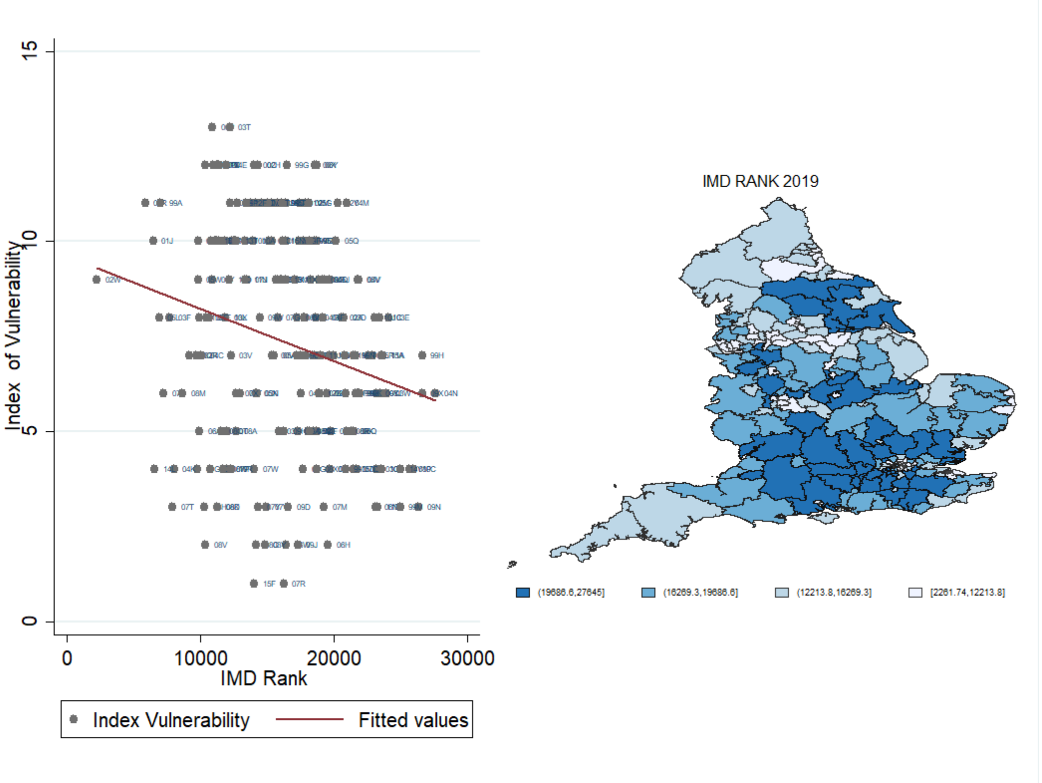

review only

Appendix

Table 1: Descriptive Statistics

	Mean	Sd	Min	Max	# Obs
Nurses per 10,000 population	3.05	0.87	1.15	6.4	191
GPs per 10,000 population	5.98	0.88	3.81	9.26	191
NHS Hospital staff per 10,000 population	191.92	236.18	0	1592.15	191
IMD Rank	16242.3	4927.95	2261.74	27645	191
COPD prevalence per 10,000 patients	201.32	66.28	79.52	406.13	191
Cancer prevalence per 10,000 patients	306.04	83.35	67.83	593.81	191
CKD prevalence per 10,000 patients	336.75	114.19	108.96	814.88	191
Diabetes prevalence per 10,000 patients	569.58	107.99	241.18	1090.75	191
Hypertension prevalence per 10,000 patients	1427.8	241.87	738.36	1857.47	191
Cardiovascular disease prevalence per 10,000 patients	137.8	35.94	56.69	216.38	191
Proportion aged 70+ per 10,000 patients	38706	27289	3845	207788	191
Ratio of residential care homes per 10,000 population aged 70+	21.11	7.15	1.52	44.73	191
Indicator of unplanned admissions for chronic disease	924.97	272.18	170.3	1666.9	191
Indicator of hospital readmission within 30 days	14.22	1.09	11.6	17.6	184

For peer review only

Indicator of mortality within 30 days of discharge	403.6	67.64	211.27	573.09	184
Indicator of acute emergency admission	1427.97	376.18	243.4	2305.8	191

Table 2: CCGs by quantile Index of Vulnerability

Index of Vulnerability			
Q1	Q2	Q3	Q4
NHS Barnet CCG	NHS Ashford CCG	NHS Airedale, Wharf..	NHS Barnsley CCG
NHS Bath and North ..	NHS Barking and Dag..	NHS Basildon and Br..	NHS Bassetlaw CCG
NHS Bexley CCG	NHS Bedfordshire CCG	NHS Birmingham and ..	NHS Blackpool CCG
NHS Brent CCG	NHS Berkshire West ..	NHS Blackburn with ..	NHS Chorley and Sou..
NHS Brighton and Ho..	NHS Bolton CCG	NHS Bradford City CCG	NHS Darlington CCG
NHS Bristol, North ..	NHS Bradford Distri..	NHS Cannock Chase CCG	NHS Doncaster CCG
NHS Bromley CCG	NHS Bury CCG	NHS Coastal West Su..	NHS Dudley CCG
NHS Buckinghamshire..	NHS Calderdale CCG	NHS Corby CCG	NHS East Lancashire..
NHS Cambridgeshire ..	NHS Canterbury and ..	NHS Coventry and Ru..	NHS Great Yarmouth ..
NHS Camden CCG	NHS Dorset CCG	NHS Derby and Derby..	NHS Lincolnshire Ea..
NHS Castle Point an..	NHS East Leicesters..	NHS Devon CCG	NHS Liverpool CCG
NHS Central London ..	NHS East Surrey CCG	NHS Durham Dales, E..	NHS Mansfield and A..
NHS City and Hackne..	NHS Eastern Cheshir..	NHS East Riding of ..	NHS Newcastle Gates..
NHS Crawley CCG	NHS Enfield CCG	NHS East Staffordsh..	NHS North Cumbria CCG
NHS Croydon CCG	NHS Gloucestershire..	NHS Eastbourne, Hai..	NHS North Durham CCG
NHS Dartford, Grave..	NHS Greater Hudders..	NHS Fylde and Wyre ..	NHS North East Esse..
NHS Ealing CCG	NHS Hambleton, Rich..	NHS Greater Preston..	NHS North East Linc..
NHS East Berkshire ..	NHS Harrogate and R..	NHS Halton CCG	NHS North Kirklees ..
NHS East and North ..	NHS Harrow CCG	NHS Hartlepool and ..	NHS North Lincolnsh..
NHS Fareham and Gos..	NHS Havering CCG	NHS Hastings and Ro..	NHS Scarborough and..

For peer review only

NHS Greenwich CCG	NHS High Weald Lewe..	NHS Herefordshire CCG	NHS South Sefton CCG
NHS Guildford and W..	NHS Ipswich and Eas..	NHS Heywood, Middle..	NHS South Tyneside ..
NHS Hammersmith and..	NHS Leicester City ..	NHS Hull CCG	NHS Southend CCG
NHS Haringey CCG	NHS Lincolnshire We..	NHS Isle of Wight CCG	NHS St Helens CCG
NHS Herts Valleys CCG	NHS Manchester CCG	NHS Kernow CCG	NHS Sunderland CCG
NHS Hillingdon CCG	NHS Nene CCG	NHS Knowsley CCG	NHS Tameside and Gl..
NHS Horsham and Mid..	NHS Newark and Sher..	NHS Luton CCG	NHS Thanet CCG
NHS Hounslow CCG	NHS Newham CCG	NHS Medway CCG	NHS Wakefield CCG
NHS Islington CCG	NHS North Hampshire..	NHS Morecambe Bay CCG	NHS Wigan Borough CCG
NHS Kingston CCG	NHS North Norfolk CCG	NHS North Staffords..	NHS Wirral CCG
NHS Lambeth CCG	NHS North West Surr..	NHS North Tyneside ..	
NHS Leeds CCG	NHS Nottingham Nort..	NHS Northumberland ..	
NHS Lewisham CCG	NHS Redditch and Br..	NHS Nottingham West..	
NHS Merton CCG	NHS Rushcliffe CCG	NHS Oldham CCG	
NHS Mid Essex CCG	NHS Salford CCG	NHS Rotherham CCG	
NHS Milton Keynes CCG	NHS Sheffield CCG	NHS Sandwell and We..	
NHS North East Hamp..	NHS Shropshire CCG	NHS Somerset CCG	
NHS Norwich CCG	NHS South Cheshire ..	NHS South East Staf..	
NHS Nottingham City..	NHS South Eastern H..	NHS South Kent Coas..	
NHS Oxfordshire CCG	NHS South Warwicksh..	NHS South Norfolk CCG	
NHS Portsmouth CCG	NHS South Worcester..	NHS South Tees CCG	
NHS Redbridge CCG	NHS Southwark CCG	NHS South West Linc..	
NHS Richmond CCG	NHS Stafford and Su..	NHS Southampton CCG	
NHS South Lincolnsh..	NHS Surrey Downs CCG	NHS Southport and F..	
NHS Surrey Heath CCG	NHS Sutton CCG	NHS Stockport CCG	
NHS Tower Hamlets CCG	NHS Swindon CCG	NHS Stoke on Trent ..	
NHS Vale of York CCG	NHS Telford and Wre..	NHS Swale CCG	
NHS Waltham Forest ..	NHS Vale Royal CCG	NHS Thurrock CCG	
NHS Wandsworth CCG	NHS West Hampshire ..	NHS Trafford CCG	
NHS Warrington CCG	NHS West Suffolk CCG	NHS Walsall CCG	
NHS West Cheshire CCG	NHS Wiltshire CCG	NHS Warwickshire No..	
NHS West Essex CCG	NHS Wolverhampton CCG	NHS West Lancashire..	
NHS West Kent CCG		NHS West Leicesters..	
NHS West London CCG		NHS West Norfolk CCG	
		NHS Wyre Forest CCG	

Figure a1: Quartile-based Index of Vulnerability

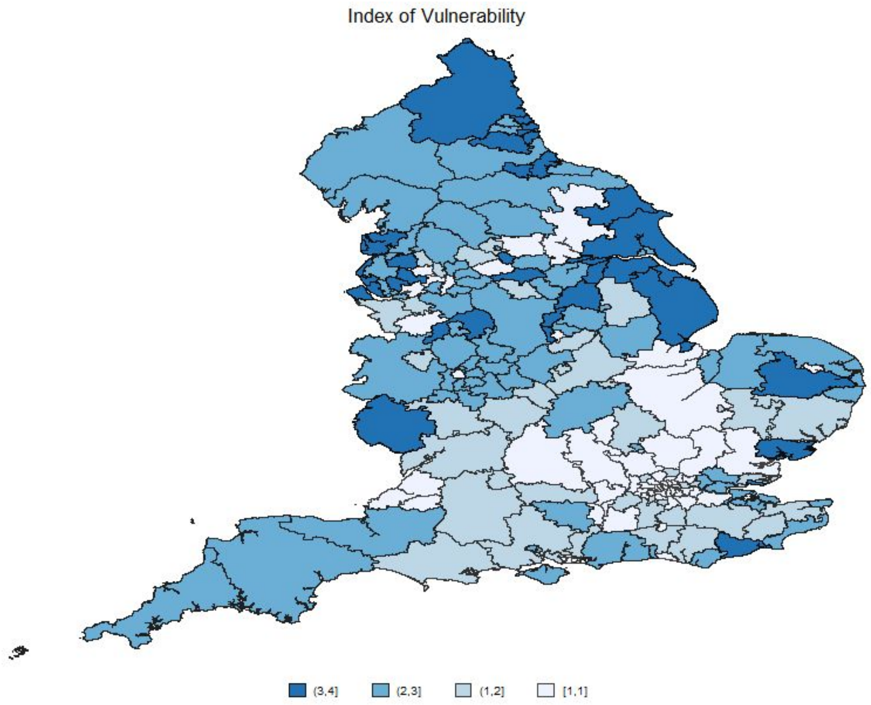
Index of Vulnerability
(3,4]
(2,3]
(1,2]
[1,1]

For peer review only

STROBE Statement—checklist of items that should be included in reports of observational studies

	Item No	Recommendation	Page No
**Title and abstract**	1	(*a*) Indicate the study's design with a commonly used term in the title or the abstract	1
		(*b*) Provide in the abstract an informative and balanced summary of what was done and what was found	2
**Introduction**			
Background/rationale	2	Explain the scientific background and rationale for the investigation being reported	3
Objectives	3	State specific objectives, including any prespecified hypotheses	3
**Methods**			
Study design	4	Present key elements of study design early in the paper	3
Setting	5	Describe the setting, locations, and relevant dates, including periods of recruitment, exposure, follow-up, and data collection	3
Participants	6	(*a*) *Cohort study*—Give the eligibility criteria, and the sources and methods of selection of participants. Describe methods of follow-up<br>*Case-control study*—Give the eligibility criteria, and the sources and methods of case ascertainment and control selection. Give the rationale for the choice of cases and controls<br>*Cross-sectional study*—Give the eligibility criteria, and the sources and methods of selection of participants	3
		(*b*) *Cohort study*—For matched studies, give matching criteria and number of exposed and unexposed<br>*Case-control study*—For matched studies, give matching criteria and the number of controls per case	NA
Variables	7	Clearly define all outcomes, exposures, predictors, potential confounders, and effect modifiers. Give diagnostic criteria, if applicable	3-4
Data sources/ measurement	8*	For each variable of interest, give sources of data and details of methods of assessment (measurement). Describe comparability of assessment methods if there is more than one group	3-4
Bias	9	Describe any efforts to address potential sources of bias	NA
Study size	10	Explain how the study size was arrived at	NA
Quantitative variables	11	Explain how quantitative variables were handled in the analyses. If applicable, describe which groupings were chosen and why	NA
Statistical methods	12	(*a*) Describe all statistical methods, including those used to control for confounding	4-7
		(*b*) Describe any methods used to examine subgroups and interactions	NA
		(*c*) Explain how missing data were addressed	NA
		(*d*) *Cohort study*—If applicable, explain how loss to follow-up was addressed<br>*Case-control study*—If applicable, explain how matching of cases and controls was addressed<br>*Cross-sectional study*—If applicable, describe analytical methods taking account of sampling strategy	NA
		(*e*) Describe any sensitivity analyses	4-5

Continued on next page

For peer review only - http://bmjopen.bmj.com/site/about/guidelines.xhtml

**Results**			
Participants	13*	(a) Report numbers of individuals at each stage of study—eg numbers potentially eligible, examined for eligibility, confirmed eligible, included in the study, completing follow-up, and analysed	NA
		(b) Give reasons for non-participation at each stage	NA
		(c) Consider use of a flow diagram	NA
Descriptive data	14*	(a) Give characteristics of study participants (eg demographic, clinical, social) and information on exposures and potential confounders	12
		(b) Indicate number of participants with missing data for each variable of interest	12
		(c) *Cohort study*—Summarise follow-up time (eg, average and total amount)	NA
Outcome data	15*	*Cohort study*—Report numbers of outcome events or summary measures over time	NA
		Case-control study—Report numbers in each exposure category, or summary measures of exposure	NA
		Cross-sectional study—Report numbers of outcome events or summary measures	NA
Main results	16	(*a*) Give unadjusted estimates and, if applicable, confounder-adjusted estimates and their precision (eg, 95% confidence interval). Make clear which confounders were adjusted for and why they were included	12-13
		(*b*) Report category boundaries when continuous variables were categorized	NA
		(*c*) If relevant, consider translating estimates of relative risk into absolute risk for a meaningful time period	NA
Other analyses	17	Report other analyses done—eg analyses of subgroups and interactions, and sensitivity analyses	4-5
**Discussion**			
Key results	18	Summarise key results with reference to study objectives	10
Limitations	19	Discuss limitations of the study, taking into account sources of potential bias or imprecision. Discuss both direction and magnitude of any potential bias	9-10
Interpretation	20	Give a cautious overall interpretation of results considering objectives, limitations, multiplicity of analyses, results from similar studies, and other relevant evidence	10
Generalisability	21	Discuss the generalisability (external validity) of the study results	10
**Other information**			
Funding	22	Give the source of funding and the role of the funders for the present study and, if applicable, for the original study on which the present article is based	11

*Give information separately for cases and controls in case-control studies and, if applicable, for exposed and unexposed groups in cohort and cross-sectional studies.

Note: An Explanation and Elaboration article discusses each checklist item and gives methodological background and published examples of transparent reporting. The STROBE checklist is best used in conjunction with this article (freely available on the Web sites of PLoS Medicine at http://www.plosmedicine.org/, Annals of Internal Medicine at http://www.annals.org/, and Epidemiology at http://www.epidem.com/). Information on the STROBE Initiative is available at www.strobe-statement.org.

For peer review only

For peer review only - http://bmjopen.bmj.com/site/about/guidelines.xhtml

BMJ Open

Measuring geographical disparities in England at the time of COVID-19: results using a composite indicator of population vulnerability

Journal:	*BMJ Open*
Manuscript ID	bmjopen-2020-039749.R1
Article Type:	Original research
Date Submitted by the Author:	25-Jun-2020
Complete List of Authors:	Nicodemo, Catia; Oxford University, Nuffield Primary Care ; CHSEO, University of Oxford<br>Barzin, Samira; Oxford University<br>Lasserson, Daniel; University of Birmingham, Institute of Applied Health Research<br>Moscone, F; Brunel University,<br>Redding, Stuart; Nuffield Department of Primary Care Health Sciences<br>Shaikh, Mujaheed; Hertie School gGmbH<br>Cavalli, Nicolo'; University of Oxford Nuffield College
**Primary Subject Heading**:	Health policy
Secondary Subject Heading:	Health economics, Infectious diseases, Public health, Sociology
Keywords:	Health policy < HEALTH SERVICES ADMINISTRATION & MANAGEMENT, HEALTH ECONOMICS, Adult intensive & critical care < INTENSIVE & CRITICAL CARE

SCHOLARONE™
Manuscripts

BMJ

I, the Submitting Author has the right to grant and does grant on behalf of all authors of the Work (as defined in the below author licence), an exclusive licence and/or a non-exclusive licence for contributions from authors who are: i) UK Crown employees; ii) where BMJ has agreed a CC-BY licence shall apply, and/or iii) in accordance with the terms applicable for US Federal Government officers or employees acting as part of their official duties; on a worldwide, perpetual, irrevocable, royalty-free basis to BMJ Publishing Group Ltd ("BMJ") its licensees and where the relevant Journal is co-owned by BMJ to the co-owners of the Journal, to publish the Work in this journal and any other BMJ products and to exploit all rights, as set out in our licence.

The Submitting Author accepts and understands that any supply made under these terms is made by BMJ to the Submitting Author unless you are acting as an employee on behalf of your employer or a postgraduate student of an affiliated institution which is paying any applicable article publishing charge ("APC") for Open Access articles. Where the Submitting Author wishes to make the Work available on an Open Access basis (and intends to pay the relevant APC), the terms of reuse of such Open Access shall be governed by a Creative Commons licence – details of these licences and which Creative Commons licence will apply to this Work are set out in our licence referred to above.

Other than as permitted in any relevant BMJ Author's Self Archiving Policies, I confirm this Work has not been accepted for publication elsewhere, is not being considered for publication elsewhere and does not duplicate material already published. I confirm all authors consent to publication of this Work and authorise the granting of this licence.

For peer review only

Measuring geographical disparities in England at the time of COVID-19: results using a composite indicator of population vulnerability

Catia Nicodemo,[1 2 *] Samira Barzin,[3] Daniel S. Lasserson,[4 5] Francesco Moscone,[6 7]

Stuart Redding,[1] Mujaheed Shaikh,[8] Nicolò Cavalli [9 10]

[1] Centre for Health Service Economics & Organisation, Nuffield Department of Primary Care Health Sciences, Oxford University, Woodstock Road, Oxford, OX2 6GG United Kingdom

[2] Department of Economics, Oxford University, Manor Road, Oxford, OX1 3UQ United Kingdom

[3] Mathematical Institute, Oxford University, Woodstock Road, Oxford, OX2 6GG United Kingdom

[4] Institute of Applied Health Research, College of Medical and Dental Sciences, University of Birmingham, Birmingham, B15 255 United Kingdom

[5] Department of Acute Medicine, Sandwell and West Birmingham NHS Trust, City Hospital, Dudley Road, Birmingham, United Kingdom

[6] Brunel University London, Kingston Lane, Uxbridge, UB83PH United Kingdom

[7] Università Ca' Foscari Venezia, Dorsoduro 3246, Venezia 30123, Italy

[8] Hertie School, Friedrichstraße 180, Berlin, 30123 Germany

[9] Nuffield College, Oxford University, 1 New Road, Oxford, OX1 1NF United Kingdom

[10] Carlo F. Dondena Centre for Research on Social Dynamics and Public Policy, Bocconi University, 1 Via Guglielmo Röntgen, Milan, 20136 Italy

*Corresponding author: catia.nicodemo@gmail.com, Centre for Health Service Economics and Organisation, Nuffield Department of Primary Care Health Sciences, University of Oxford, Gibson Building, Radcliffe Observatory Quarter, Woodstock Road, Oxford OX2 6GG, Tel+447775158584

For peer review only

Abstract

Objectives – The growth of COVID-19 infections in England raises questions about system vulnerability. Several factors that vary across geographies, such as age, existing disease prevalence, medical resource availability, and deprivation, can trigger adverse effects on the National Health System during a pandemic. In this paper, we present data on these factors and combine them to create an index to show which areas are more exposed. This technique can help policy makers to moderate the impact of similar pandemics.

Design – We combine several sources of data, which describe specific risk factors linked with the outbreak of a respiratory pathogen, that could leave local areas vulnerable to the harmful consequences of large-scale outbreaks of contagious diseases. We combine these measures to generate an index of community-level vulnerability.

Setting – 191 Clinical Commissioning Groups (CCGs) in England.

Main outcome measures – We merge 15 measures spatially to generate an index of community-level vulnerability. These measures cover prevalence rates of high-risk diseases; proxies for the at-risk population density; availability of staff and quality of healthcare facilities.

Results – We find that the North and South West of England are among the most vulnerable areas due to the ageing population who live there and their high prevalence rates of diseases. These areas, together with the East Coast areas of Lancashire, also appear particularly vulnerable to adverse shocks to healthcare supply. When turning to the synthetic index, we find that the most vulnerable areas are in the East of England.

Conclusions – Evidence-based policy is crucial to mitigate the health impact of pandemics such as COVID-19. While current attention focuses on curbing rates of contagion, we introduce a vulnerability index combining data that can help policy makers identify the most vulnerable communities. We find that this index is positively correlated with COVID-19 deaths and the index of deprivation.

Keywords: Geographical disparity; Pandemic; Emergency preparedness; Index of vulnerability; Health inequalities; Covid-19.

For peer review only

Word count: 3,365

Strengths and limitations of this study

- We provide important information to help identify the communities most vulnerable to harmful effects of COVID-19
- This fills an important gap in the literature, with only a handful of previous studies that show the distribution of underlying risks within national systems
- Judgement was required when deciding which variables to include or omit
- Our methods give equal weight to each variable when creating the index
- CCGs are quite large and using smaller areas may be more appropriate

For peer review only

1 Introduction

The current COVID-19 outbreak is triggering a renewed understanding of health risks and underlying health vulnerabilities.[1] In a pandemic, overlooked vulnerabilities may arise from the social and biological makeup of local communities. Accumulated evidence from emerging and re-emerging infectious diseases, such as SARS, swine influenza, MERS and now COVID-19, indicates that infections requiring critical care, and associated case fatalities, are usually, but not exclusively, concentrated in elderly patients, and in patients suffering from specific comorbidities, such as chronic obstructive pulmonary disease, cardiovascular diseases, diabetes, cancer, chronic kidney disease.[2–5]

Existing evidence further suggests that infectious disease outbreaks might lead to adverse shocks to the supply of healthcare. Healthcare workers are directly exposed to risk of transmission and may suffer from the physical and psychological impact of sudden surges in workloads that come with high case fatalities.[6,7] Lack of resources available to healthcare providers and suboptimal quality of health services in a community represent a further source of vulnerability, which hamper the supply of critical care, puts patients at higher risk of negative health outcomes, and endanger the safety of health workers.[8,9]

Despite the fact that, in epidemic intelligence, local disease maps have been in use at least since seminal work by Seaman and Pascalis-Ouvière, who in 1796-7 employed spot maps to trace cases of yellow fever in New York and Philadelphia,[10,11] interest in how community level variables may moderate the outcomes of infectious diseases was limited before COVID-19, with only a handful of studies reconstructing the distribution of health risks and vulnerabilities across communities in a broad range of settings.[9,11–14] Recent studies have started to address this gap.[15]

In this paper we focus on England, a large part of the United Kingdom which by June 1st 2020 was the third-worst hit country for per capita COVID-19 deaths.[16] We collect administration information at the level of the Clinical Commissioning Groups (CCG), the administrative units that provide National Health System (NHS) services in England, and combine 15 vulnerability indicators in a synthetic Index of Vulnerability.[17] We report geographic vulnerabilities across 191 CCGs and match them with COVID-19 death data up to the 23rd week of 2020. We find a positive relationship between our indicator of vulnerability and the COVID-19 related death rate over the local population, with a correlation coefficient of around 10%.

We also find that vulnerability is not randomly distributed across geographies. Socio-economically deprived areas display higher prevalence of pre-existing health conditions and lower access to healthcare services. This makes deprived communities disproportionately vulnerable to critical infections and case fatalities during an infectious disease outbreak.[18–24] Identifying these vulnerabilities ahead of time may help to shape preparedness and response policies and, during a pandemic, may be key to fairly allocating the stretched resources of a nation's health systems across communities in need.[14,25–27] In

For peer review only - http://bmjopen.bmj.com/site/about/guidelines.xhtml

Section 2, we describe the methods used to build our Index of Vulnerability, which we present in Section 3. In Section 4, we discuss our results and conclude.

2 Methods

Using data gathered from NHS Digital and focusing on the geographic level of Clinical Commissioning Group areas for England (CCGs, *n*=191) in the financial year 2018/2019, we combine area-level indicators of vulnerability in an Index of Vulnerability to obtain a standardised synthetic vulnerability measure for each CCG in England. To select relevant vulnerability indicators, which we combine in our Index, we queried Pubmed for articles containing the terms 'health', ''inequality', 'vulnerability' and 'pandemics', without language or geographic restrictions. Our search returned 105 results: whereas just 62 studies were published from 1993 to 2019, 43 further papers have been published on the COVID-19 pandemic.

These studies identify three macro-categories of factors that drive geographic differences in health vulnerabilities: i) disparities in susceptibility to a disease; ii) disparities in the likelihood of contracting a disease, and iii) disparities in treatment.[28] Factors that drive disparities in susceptibility include the prevalence of certain pre-existing diseases[29–32] and local demographics, such as a community's age structure.[11,29,31,33–37] There is evidence that the elderly have been hit heavily by the COVID-19 pandemic.[5,37,38] Therefore, in building our Index, we consider i) the number of people above 70 years old per 10,000 residents across CCGs.

We also consider the prevalence of patients suffering from ii) cardiovascular diseases; iii) chronic obstructive pulmonary disease (COPD); iv) cancer; v) chronic kidney disease (CKD) for patients over the age of 18; vi) hypertension; and viii) diabetes (patients aged 17 or older). These are identified by Public Health England, the English health authority, as characterised by higher risks of severe illness from SARS-CoV-2.[39] To track existing conditions, we used Quality and Outcomes Framework prevalences (QOF), computed as the number of patients registered in a General Practice with a certain health condition at a given time, divided by the total patients registered at the same GP and time.

Risk factors associated with the likelihood of contracting the disease include the patterns of population density and mobility;[29,36,40–42] whether a community is principally urban or rural.[11,32,43] Thus, we consider ix) an urban to rural indicator among our vulnerability factors. We also include x) the ratio of residential and nursing homes per 10,000 population aged over 70 years old. Although there is evidence that a good long-term care infrastructure could reduce hospital admissions and mortality,[44] during pandemic a higher density of home cares represents a risk factor due to the frailty of their residents and observed difficulties in stopping within-facility transmission.[29,32,45] During the ongoing pandemic in England, it also has been suggested that freeing up strained hospital capacity by discharging older patients into care homes may have exacerbated the spread of the disease, although the

For peer review only - http://bmjopen.bmj.com/site/about/guidelines.xhtml

evidence on this is still mixed.[38,46,47] Based on this, in our Index of Vulnerability, we leverage ix) the ratio of residential and nursing homes per 10,000 population aged over 70 years old; x) an urban to rural indicator.

While variables i)-x) measure quantities that affect the 'demand' of healthcare during a pandemic, extant literature also emphasises the relevance of a set of 'supply'-side factors. Among these, resources available to local healthcare systems, such as the number of intensive care beds and the size of the healthcare workforce, are considered to shape the capacity of local systems to absorb surges in demand.[31,33,40,48,49] The overall quality of a local health system also matters, as high quality practices shield inpatients from the downside risks of infections, and better protect their healthcare workforce.[29]

To build our Index of Vulnerability, we focus on indicators capturing the NHS workforce in each CCG, and on a set of proxies for the quality of healthcare supply. To measure NHS workforce, we measure (xi) the number of nurses within General Practices, general practitioners (GPs) and hospital staff, including non-medical personnel. We consider full-time equivalent equivalents (FTEs), which is a more accurate measure of workforce availability compared to raw headcounts, especially in more deprived and rural areas, where GPs frequently work part-time. We weighted each of these parameters by the CCG-area population. However, while GPs and practice nurses are measured directly at the CCG level, NHS hospital staff is measured at NHS Hospital Trust level. We used the provider code, and the postcode to link the NHS Trusts to the appropriate CCG area.

Following previous literature,[50] we measure quality by including in our Index xii) the percentage of emergency hospital admissions occurring within 30 days of the most recent discharge; xiii) the ratio of deaths within 30 days of a non-elective hospital procedure in 2014 (latest data point available); xiv) the prevalence of unplanned admissions for ambulatory care sensitive conditions; and xv) emergency admissions for acute conditions that should not usually require hospital admission. Measures xiv) and xv) capture acute hospitalisations in patients with specific long-term conditions, when primary care and the delivery of appropriate hospital outpatient services could have prevented deterioration and avoid hospitalization.[51] All the indicators of quality are indirectly standardised rates based on age and sex specific rates in England.

We combine these 15 indicators in a synthetic index of area-level vulnerability (see Table S1 in the supplementary file for descriptive statistics of all indicators used in this study). To build this index, we first dichotomize each variable by comparing it to the mean of the series in the whole of England. For each variable, value "one" (more risk) was assigned to CCGs with higher than average target diseases, higher than average elderly population; to CCGs in mostly urban areas; with higher than average prevalence of home and nursing care facilities; lower-than-average healthcare personnel FTEs; and lower than average hospital quality. We assigned value "zero" for all indicators below the mean.

Building on OECD guidelines,[52] we obtained a synthetic Index of Vulnerability as the arithmetic sum of these dummies for each CCG area (*min*=0; *max*=15, *mean*=7.38).

For peer review only

Therefore, our index represents a functional transformation that, for each CCG area, maps the vector of dichotomous indicators into an integer number. Above-or-below mean indices are a class of additive aggregation methods considered to be robust to outliers. However, they suffer from substantial information loss on the relative distance of each CCG from the national average. We address this issue by presenting a quartile-based indicator in the Supplementary file, which offers similar results to the above-or-below average index and thus provide support for our chosen quantification approach.

Patient and Public Involvement – patients and the public were not involved in this research. We would expect that the information provided here can help encourage a public debate when considering what other variables may deserve inclusion in similar indices in the future.

3 Results

3.1 Spatial analysis of vulnerabilities in England's CCGs

In the supplementary file Figures S1-S4, we plot maps for each of the 15 indicators described in the Methods Section above. We show that, in Egland, residential and nursing facilities are concentrated in the North and the South West of England, areas also more populated by the elderly. However, looking at the ratio of homecare facilities over the older population, we observe that areas in the North and East Coast have the lowest ratios (Figure S1). Figure S2 depicts the CCG-level distribution of population prevalence for six diseases chosen with the aim of assessing the susceptibility of the system to healthcare demand shocks from the spread of COVID-19.

We find that the areas with the largest prevalence of these underlying diseases, especially cardiovascular diseases, hypertension and chronic kidney disease, are again located in the North and South West of the country. CCGs in the East of the country, including the East Midlands as well as the Yorkshire and Humber areas are particularly affected by diabetes, COPD and cancer, implying a higher underlying health risk for the population living in these areas, compared to the English average. Increases in the demand for critical care driven by the interaction within infectious diseases and underlying comorbidities raise the question of whether the existing supply of healthcare is adequate, or whether capacity building should be considered.

In Figure S3 in the supplementary file, we report on the availability of GPs, nurses in general practices and medical personnel in hospitals. We observe that the healthcare workers are not equally spatially distributed across different areas in England. GPs are more concentrated in the North, the Midlands, and the South West. GP Nurses are spread more evenly across the country, and levels of hospital staff are higher in the South and South East, with the lowest values observed in the coastal areas of the East Midlands and the North East of England.

For peer review only

Finally, in Figure S4, we plot four indicators of hospital quality. We show that there is high variation in these quality markers across England. The North East and North West region of the country (North Kirklees and South Sefton CCGs, for example), report a high number of unplanned hospitalisations, acute emergency admissions and a higher rate of deaths after discharge. High mortality rates appear to be spread more widely, with high levels not only in the North but also in the South (e.g. Sussex and Essex CCGs), and especially in the areas of the South West (Devon CCG).

3.2 A synthetic Index of Vulnerability for England

In Figure 1, we map the synthetic Index of Vulnerability, obtained with the procedure described in the Methods Section 2. According to this Index, CCGs in the North and the South of England appear most vulnerable to an infectious disease outbreak, with Sunderland, East and North Lincolnshire East, and Wigan Borough among the most vulnerable. Table 1 reports the list of all CCGs by vulnerability score, divided in quartiles. In the first quartile (Q1), we find CCG areas with lower Index of Vulnerability and in Q4 the CCGs area with the highest values. These last group of CCGs should be targeted by the NHS to improve their outcomes and increase the measures of protection in case of pandemic. Figure S5 in the supplementary file maps the inter-quartile index of vulnerability at CCG level, with comparable findings.

[FIGURE 1 HERE]

For peer review only

Table 1: CCGs by quantile Index of Vulnerability

**Quartile**	**CCGs**
**Q1**	Barnet; Bath and North East Somerset; Bexley; Brent; Brighton and Hove; Bristol, North Somerset and South Gloucestershire; Bromley; Buckinghamshire; Cambridgeshire and Peterborough; Camden; Castle Point and Rochford; Central London (Westminster); City and Hackney; Crawley; Croydon; Dartford, Gravesham and Swanley; Ealing; East Berkshire; East and North Hertfordshire; Fareham and Gosport; Greenwich; Guildford and Waverley; Hammersmith and Fulham; Haringey; Herts Valleys; Hillingdon; Horsham and Mid Sussex; Hounslow; Islington; Kingston; Lambeth; Leeds; Lewisham; Merton; Mid Essex; Milton Keynes; North East Hampshire; Norwich; Nottingham City; Oxfordshire; Portsmouth; Redbridge; Richmond; South Lincolnshire; Surrey Heath; Tower Hamlets; Vale of York; Waltham Forest; Wandsworth; Warrington; West Cheshire; West Essex; West Kent; West London.

**Q2**	Ashford; Barking and Dagenham; Bedfordshire; Berkshire West; Bolton; Bradford District; Bury; Calderdale; Canterbury and Coastal; Dorset; East Leicestershire and Rutland; East Surrey; Eastern Cheshire; Enfield; Gloucestershire; Greater Huddersfield; Hambleton, Richmondshire and Whitby; Harrogate and Rural District; Harrow; Havering; High Weald Lewes Havens; Ipswich and East Suffolk; Leicester City; Lincolnshire West; Manchester; Nene; Newark and Sherwood; Newham; North Hampshire; North Norfolk; North West Surrey; Nottingham North and East; Redditch and Bromsgrove; Rushcliffe; Salford; Sheffield; Shropshire; South Cheshire; South Eastern Hampshire; South Warwickshire; South Worcestershire; Southwark; Stafford and Surrounds; Surrey Downs; Sutton; Swindon; Telford and Wrekin; Vale Royal; West Hampshire; West Suffolk; Wiltshire; Wolverhampton.
**Q3**	Airedale, Wharfedale and Craven; Basildon and Brentwood; Birmingham and Solihull; Blackburn with Darwen; Bradford City; Cannock Chase; Coastal West Sussex; Corby; Coventry and Rugby; Derby and Derbyshire; Devon; Durham Dales, Easington and Sedgefield; East Riding of Yorkshire; East Staffordshire; Eastbourne, Hailsham and Seaford; Fylde and Wyre; Greater Preston; Halton; Hartlepool and Stockton-on-Tees; Hastings and Rother; Herefordshire; Heywood, Middleton and Rochdale; Hull; Isle of Wight; Kernow; Knowsley; Luton; Medway; Morecambe Bay; North Staffordshire; North Tyneside; Northumberland; Nottingham West; Oldham; Rotherham; Sandwell and West Birmingham; Somerset; South East Staffordshire and Seisdon Peninsula; South Kent Coast; South Norfolk; South Tees; South West Lincolnshire; Southampton; Southport and Formby; Stockport; Stoke on Trent; Swale; Thurrock; Trafford; Walsall; Warwickshire North; West Lancashire; West Leicestershire; West Norfolk; Wyre Forest.
**Q4**	Barnsley; Bassetlaw; Blackpool; Chorley and South Ribble; Darlington; Doncaster; Dudley; East Lancashire; Great Yarmouth and Waveney; Lincolnshire East; Liverpool; Mansfield and Ashfield; Newcastle Gateshead; North Cumbria; North Durham; North East Essex; North East Lincolnshire; North Kirklees; North Lincolnshire; Scarborough and Ryedale; South Sefton; South Tyneside; Southend; St Helens; Sunderland; Tameside and Glossop; Thanet; Wakefield; Wigan Borough; Wirral.

Source: Own elaboration based on NHS digital data at CCGs areas 2019.

To test whether CCGs with similar vulnerability levels are more spatially clustered than expected by chance, we computed Moran's I statistic, one of the most widely adopted tests of spatial correlation between observations. Moran's I can be seen as a correlation index that compares statistical units by weighting each pair by a distance function specified by the means of a spatial weight matrix.[53] We have adopted the contiguity criterium to build the spatial weights matrix and standardized it so that the sum of the elements for each row is unity. Results show that Moran's I, calculated on our vulnerability index, is significant, indicating positive spatial correlation (I=0.155, with a p-value of 0.00). This implies that geographically contiguous CCGs tend to show similarly high levels of vulnerability.

For peer review only

3.3 Index of Vulnerability and Deaths to COVID-19

Although this paper was conceived in March 2020, before that the pandemic hit hard in England, we check if the vulnerability index could predict the mortality rate due to COVID-19 across areas up to June 2020. We use the deaths registered data in the first 22 weeks of 2020 across CCG areas from Official National Statistics (ONS), where the numbers of deaths for COVID-19 are reported. In Figure 2, we can observe a positive correlation between mortality rate due to COVID-19 and the Index of Vulnerability. However, some caution needs to be taken when we interpret this result because the data present some limitations. For example, not all deaths have been tested for COVID-19, and this is particularly true for those that happen at home. Most positive tests are recorded in hospitals and this means they are often not reported for the patient's area of residence but rather the CCG in which the hospital is located. These limitations could affect the correlation with the Index of Vulnerability as it is not possible to know accurately the residence of patients with COVID-19.

[FIGURE 2 HERE]

4 Discussion

In this article, we have presented an Index of Vulnerability to map the vulnerability of the English healthcare system to the unexpected and combined consequences of demand- and supply-related pressures associated with infectious disease outbreaks such as the COVID-19 pandemic. To cope with the pandemic, public health authorities across the country have engaged in significant capacity-building efforts, including opening three temporary hospitals that thankfully were not required, but this demonstrates the type of prompt and decisive action that policymakers should be prepared to pursue when faced with pandemics. In order to maximise the effectiveness of these actions, policy makers need evidence such as that presented in this paper so that limited resources are provided to the areas where they are needed the most.

Evidence from previous infectious disease outbreaks, such as Malaria, warns that funding during emergencies tend to be provided in line with health and economic need, but biased towards richer areas.[54] In light of the correlation between CCG-level vulnerability and deprivation reported in this paper, the distribution of resources in times of emergencies should be guided by community-level factors. Guidelines for 'fair allocation of resources' should be developed based on direct knowledge about local communities - not only considering the health impact of such choices, but also long-run economic and social impacts for the community.

Communities and local health care providers should also be involved in these decisions, because community-level knowledge will aid in choosing the right guiding principle - for

For peer review only

example, knowledge of population demographics and illness prevalence within a locality might help when deciding whether to maximize benefits/prognosis; to give priority to the worst off; or to reward value to the community. Accurate data collection and dynamic information sharing mechanisms between CCGs - not only of diagnosed cases, but also of staff, medical supplies and excess capacity - will help spread the disease burden and prevent the entire system from collapsing.

No matter what level of community spread has been reached, CCGs should be prepared to act at least in accordance with WHO guidelines.[55] As a minimum, this includes a coordinated strategy across hospitals and communities, and between hospitals and healthcare staff within the community. Planning and stocking medical equipment and drug supplies that are necessary to treat not only COVID-19 patients, but also those with other predisposing illnesses at the local community level is key. Consulting our indicator would allow policymakers and healthcare managers to understand which areas might require strengthened surveillance, monitoring and capacity building so as to minimise the negative outcomes of unexpected health shocks.

Attention should however be paid to the limitations of our Index. The main limitation stems from the selection of indicators of vulnerability. Although our choice was driven by literature, idiosyncratic choices and partial knowledge open the possibility of omitting relevant variables. For example, unlike some existing research,[34] we have not included the availability of beds as a factor that generates supply-side pressures. The reason for this choice is that responses to the COVID-19 crisis suggest that the elasticity in the supply of beds is higher than that of the supply of workforce. In fact, while both bed and healthcare personnel can be increased during a crisis, solutions that could feasibly increase the latter (such as speeding up the promotion of trainees or recalling retirees) have suboptimal implications for quality and organisational efficiency. Moreover, healthcare workers are more exposed to infections, which could create staff shortages, although the hard work of practitioners and managers avoided this to happen in England during the pandemic.

Second, an increasing body of evidence reports a higher risk of infection with COVID-19 and higher mortality in individuals belonging to certain disadvantaged ethnic groups.[19,23,24,56–59] However, rather than revealing ethnic and racial disparities, these differences could reflect underlying socio-economic inequalities, in some cases higher prevalences of pre-existing and at risk health conditions. Other indicators, such as indices of deprivation, might be promising to investigate intersectional vulnerabilities to COVID-19. Deprivation may in fact interact with the vulnerabilities contained in our Index. In fact, social deprivation is associated with the timing of the onset of multimorbidities and the prevalence of long-term conditions.[60] Deprivation also matters for the higher usage of emergency care relative to elective care, 30% of which is not explained by the different case-mix of conditions suffered by patients from a given area.[61] In addition, recruitment of clinicians to deprived areas presents challenges.[62]

In Figure 3, we plot our Index of Vulnerability against the Index of Multiple Deprivation rank (IMD).[63] We show a positive correlation between the two indices. For example, the

For peer review only

North East of England shows high levels of both vulnerability and deprivation. Calculating Moran's I local estimation (LISA)[64] for both indices we detected a positive, although weak (about 20%), statistically significant correlation between the two local spatial indices. This shows that there exists a moderate overlap between the clusters detected by the two indices, which we argue indicates that these indices present complementary evidence rather than substitutable evidence. Moreover, deprivation does not appear to be correlated with mortality from COVID-19 (see supplementary file). Intersectional analysis aimed at evaluating the potentially overlapping role of these factors is a fruitful area of research as efforts to collect more granular data are mounting.

[FIGURE 3 HERE]

References

1. The Lancet. Redefining vulnerability in the era of COVID-19. The Lancet. 2020 Apr;395(10230):1089.
2. Lau EH, Hsiung CA, Cowling BJ, Chen C-H, Ho L-M, Tsang T, et al. A comparative epidemiologic analysis of SARS in Hong Kong, Beijing and Taiwan. BMC Infect Dis. 2010 Dec;10(1):50.
3. Tekin S, Keske S, Alan S, Batirel A, Karakoc C, Tasdelen-Fisgin N, et al. Predictors of fatality in influenza A virus subtype infections among inpatients in the 2015–2016 season. Int J Infect Dis. 2019 Apr;81:6–9.
4. Rivers CM, Majumder MS, Lofgren ET. Risks of Death and Severe Disease in Patients With Middle East Respiratory Syndrome Coronavirus, 2012–2015. Am J Epidemiol. 2016 Sep 15;184(6):460–4.
5. Zhou F, Yu T, Du R, Fan G, Liu Y, Liu Z, et al. Clinical course and risk factors for mortality of adult inpatients with COVID-19 in Wuhan, China: a retrospective cohort study. The Lancet. 2020 Mar;395(10229):1054–62.
6. Nacoti Mirco, Ciocca Andrea, Giupponi Angelo, Brambillasca Pietro, Lussana Federico, Pisano Michele, et al. At the Epicenter of the Covid-19 Pandemic and Humanitarian Crises in Italy: Changing Perspectives on Preparation and Mitigation. N Engl J Med Catal [Internet]. [cited 2020 Mar 26];1(2). Available from: https://doi.org/10.1056/CAT.20.0080
7. Rosenbaum L. Facing Covid-19 in Italy — Ethics, Logistics, and Therapeutics on the Epidemic's Front Line. N Engl J Med. 2020 May 14;382(20):1873–5.

For peer review only

8. Morens DM, Folkers GK, Fauci AS. The challenge of emerging and re-emerging infectious diseases. Nature. 2004 Jul;430(6996):242–9.

9. Mareiniss DP, Hirshon JM, Thibodeau BC. Disaster planning: potential effects of an influenza pandemic on community healthcare resources. Am J Disaster Med. 2009 Jun;4(3):163–71.

10. Shannon GW. Disease mapping and early theories of Yellow Fever. Prof Geogr. 1981 May;33(2):221–7.

11. Timpka T, Eriksson H, Strömgren M, Eriksson O, Ekberg J, Grimvall A, et al. A neighborhood susceptibility index for planning of local physical interventions in response to pandemic influenza outbreaks. AMIA Annu Symp Proc AMIA Symp. 2010 Nov 13;2010:792–6.

12. Bizimana J-P, Twarabamenye E, Kienberger S. Assessing the social vulnerability to malaria in Rwanda. Malar J. 2015 Dec;14(1):2.

13. Hagenlocher M, Delmelle E, Casas I, Kienberger S. Assessing socioeconomic vulnerability to dengue fever in Cali, Colombia: statistical vs expert-based modeling. Int J Health Geogr. 2013;12(1):36.

14. Hayward AC, Fragaszy EB, Bermingham A, Wang L, Copas A, Edmunds WJ, et al. Comparative community burden and severity of seasonal and pandemic influenza: results of the Flu Watch cohort study. Lancet Respir Med. 2014 Jun;2(6):445–54.

15. Franch-Pardo I, Napoletano BM, Rosete-Verges F, Billa L. Spatial analysis and GIS in the study of COVID-19. A review. Sci Total Environ. 2020 Oct;739:140033.

16. Dong E, Du H, Gardner L. An interactive web-based dashboard to track COVID-19 in real time. Lancet Infect Dis. 2020 Feb;S1473309920301201.

17. Nicodemo C, Barzin S, Lasserson DS, Moscone F, Redding S, Shaikh M, et al. Population Vulnerability to Unexpected Health Shocks: Geographical Disparities in England at the Time of COVID-19. SSRN Electron J [Internet]. 2020 [cited 2020 Jun 21]; Available from: https://www.ssrn.com/abstract=3571524

18. Rutter PD, Mytton OT, Mak M, Donaldson LJ. Socio-economic disparities in mortality due to pandemic influenza in England. Int J Public Health. 2012 Aug;57(4):745–50.

19. Khalatbari-Soltani S, Cumming RG, Delpierre C, Kelly-Irving M. Importance of collecting data on socioeconomic determinants from the early stage of the COVID-19 outbreak onwards. J Epidemiol Community Health. 2020 May 8;jech-2020-214297.

20. Chung RY-N, Dong D, Li MM. Socioeconomic gradient in health and the covid-19 outbreak. BMJ. 2020 Apr 1;m1329.

For peer review only

21. Andayi F, Crepey P, Kieffer A, Salez N, Abdo AA, Carrat F, et al. Determinants of individuals' risks to 2009 pandemic influenza virus infection at household level amongst Djibouti city residents - A CoPanFlu cross-sectional study. Virol J. 2014 Dec;11(1):13.

22. Zhao H, Harris RJ, Ellis J, Pebody RG. Ethnicity, deprivation and mortality due to 2009 pandemic influenza A(H1N1) in England during the 2009/2010 pandemic and the first post-pandemic season. Epidemiol Infect. 2015 Dec;143(16):3375–83.

23. Gray DM, Anyane-Yeboa A, Balzora S, Issaka RB, May FP. COVID-19 and the other pandemic: populations made vulnerable by systemic inequity. Nat Rev Gastroenterol Hepatol [Internet]. 2020 Jun 15 [cited 2020 Jun 21]; Available from: http://www.nature.com/articles/s41575-020-0330-8

24. Dorn A van, Cooney RE, Sabin ML. COVID-19 exacerbating inequalities in the US. The Lancet. 2020 Apr;395(10232):1243–4.

25. Emanuel EJ, Persad G, Upshur R, Thome B, Parker M, Glickman A, et al. Fair Allocation of Scarce Medical Resources in the Time of Covid-19. N Engl J Med. 2020 May 21;382(21):2049–55.

26. Wang Z, Tang K. Combating COVID-19: health equity matters. Nat Med. 2020 Apr;26(4):458–458.

27. Hill AB. The environment and disease: association or causation? Proc R Soc Med. 1965 May;58:295–300.

28. Blumenshine P, Reingold A, Egerter S, Mockenhaupt R, Braveman P, Marks J. Pandemic Influenza Planning in the United States from a Health Disparities Perspective. Emerg Infect Dis. 2008 May;14(5):709–15.

29. Desmet K, Wacziarg R. Understanding Spatial Variation in COVID-19 across the United States [Internet]. Cambridge, MA: National Bureau of Economic Research; 2020 Jun [cited 2020 Jun 20] p. w27329. Report No.: w27329. Available from: http://www.nber.org/papers/w27329.pdf

30. Shigemura J, Ursano RJ, Morganstein JC, Kurosawa M, Benedek DM. Public responses to the novel 2019 coronavirus (2019-nCoV) in Japan: Mental health consequences and target populations. Psychiatry Clin Neurosci. 2020 Apr;74(4):281–2.

31. Renzaho AMN. The Need for the Right Socio-Economic and Cultural Fit in the COVID-19 Response in Sub-Saharan Africa: Examining Demographic, Economic Political, Health, and Socio-Cultural Differentials in COVID-19 Morbidity and Mortality. Int J Environ Res Public Health. 2020 May 15;17(10):3445.

32. Solis J, Franco-Paredes C, Henao-Martínez AF, Krsak M, Zimmer SM. Structural

For peer review only

Vulnerability in the United States Revealed in Three Waves of Novel Coronavirus Disease (COVID-19). Am J Trop Med Hyg [Internet]. 2020 May 7 [cited 2020 Jun 21]; Available from: http://www.ajtmh.org/content/journals/10.4269/ajtmh.20-0391

33. Lakhani A. Which Melbourne Metropolitan Areas Are Vulnerable to COVID-19 Based on Age, Disability, and Access to Health Services? Using Spatial Analysis to Identify Service Gaps and Inform Delivery. J Pain Symptom Manage. 2020 Jul;60(1):e41–4.
34. Verhagen MD, Brazel DM, Dowd JB, Kashnitsky I, Mills M. Forecasting spatial, socioeconomic and demographic variation in COVID-19 health care demand in England and Wales [Internet]. Open Science Framework; 2020 Mar [cited 2020 Jun 21]. Available from: https://osf.io/g8s96
35. Dowd JB, Andriano L, Brazel DM, Rotondi V, Block P, Ding X, et al. Demographic science aids in understanding the spread and fatality rates of COVID-19. Proc Natl Acad Sci. 2020 May 5;117(18):9696–8.
36. Santos JPC dos, Siqueira ASP, Praça HLF, Albuquerque HG. Vulnerabilidade a formas graves de COVID-19: uma análise intramunicipal na cidade do Rio de Janeiro, Brasil. Cad Saúde Pública. 2020;36(5):e00075720.
37. Lim W, Liang C, Assantachai P, Auyeung TW, Kang L, Lee W, et al. COVID-19 and older people in Asia: Asian Working Group for Sarcopenia calls to actions. Geriatr Gerontol Int. 2020 Jun;20(6):547–58.
38. Docherty AB, Harrison EM, Green CA, Hardwick HE, Pius R, Norman L, et al. Features of 16,749 hospitalised UK patients with COVID-19 using the ISARIC WHO Clinical Characterisation Protocol [Internet]. Infectious Diseases (except HIV/AIDS); 2020 Apr [cited 2020 Jun 22]. Available from: http://medrxiv.org/lookup/doi/10.1101/2020.04.23.20076042
39. Public Health England. Guidance on social distancing for everyone in the UK. Updated March 30, 2020 [Internet]. 2020. Available from: https://www.gov.uk/government/publications/covid-19-guidance-on-social-distancing-and-for-vulnerable-people/guidance-on-social-distancing-for-everyone-in-the-uk-and-protecting-older-people-and-vulnerable-adults
40. Ren H, Zhao L, Zhang A, Song L, Liao Y, Lu W, et al. Early forecasting of the potential risk zones of COVID-19 in China's megacities. Sci Total Environ. 2020 Aug;729:138995.
41. Gómez JM, Verdú M. Network theory may explain the vulnerability of medieval human settlements to the Black Death pandemic. Sci Rep. 2017 Apr;7(1):43467.

For peer review only

42. Hintzen PC. Precarity and the HIV/AIDS pandemic in the Caribbean: Structural stigma, constitutionality, legality in development practice. Glob Public Health. 2019 Nov 2;14(11):1624–38.
43. Peters DJ. Community Susceptibility and Resiliency to COVID-19 Across the Rural-Urban Continuum in the United States. J Rural Health. 2020 Jun 16;jrh.12477.
44. Barker I, Steventon A, Deeny SR. Association between continuity of care in general practice and hospital admissions for ambulatory care sensitive conditions: cross sectional study of routinely collected, person level data. BMJ. 2017 Feb 1;j84.
45. McMichael TM, Clark S, Pogosjans S, Kay M, Lewis J, Baer A, et al. COVID-19 in a Long-Term Care Facility — King County, Washington, February 27–March 9, 2020. MMWR Morb Mortal Wkly Rep. 2020 Mar 27;69(12):339–42.
46. National Audit Office. Readying the NHS and adult social care in England for COVID-19 [Internet]. 2020. Available from: https://www.nao.org.uk/wp-content/uploads/2020/06/Readying-the-NHS-and-adult-social-care-in-England-for-COVID-19.pdf
47. NHS Providers. Recent NHS discharghes into care homes [Internet]. 2020. (Coronavirus briefing). Available from: https://nhsproviders.org/media/689548/spotlight-on-recent-discharges-into-care-homes-nhs-providers.pdf
48. Marmot M. Society and the slow burn of inequality. The Lancet. 2020 May;395(10234):1413–4.
49. Kang J-Y, Michels AC, Lyu F, Wang S, Agbodo N, Freeman VL, et al. Rapidly Measuring Spatial Accessibility of COVID-19 Healthcare Resources: A Case Study of Illinois, USA [Internet]. Public and Global Health; 2020 May [cited 2020 Jun 20]. Available from: http://medrxiv.org/lookup/doi/10.1101/2020.05.06.20093534
50. Gutacker N, Siciliani L, Moscelli G, Gravelle H. Choice of hospital: Which type of quality matters? J Health Econ. 2016 Dec;50:230–46.
51. Sundmacher L, Fischbach D, Schuettig W, Naumann C, Augustin U, Faisst C. Which hospitalisations are ambulatory care-sensitive, to what degree, and how could the rates be reduced? Results of a group consensus study in Germany. Health Policy. 2015 Nov;119(11):1415–23.
52. European Commission, Organisation for Economic Co-operation and Development, SourceOECD (Online service), editors. Handbook on constructing composite indicators: methodology and user guide. Paris: OECD; 2008. 158 p.
53. Moran PAP. Notes on Continuous Stochastic Phenomena. Biometrika. 1950

For peer review only

Jun;37(1/2):17.

54. Barrenho E, Miraldo M, Shaikh M, Atun R. Vertical and horizontal equity of funding for malaria control: a global multisource funding analysis for 2006–2010. BMJ Glob Health. 2017 Dec;2(4):e000496.

55. World Health Organisation. Responding to community spread of COVID-19. Interim guidance. [Internet]. 2020. Available from: https://www.dropbox.com/home/covid_uk/literature?preview=WHO-COVID-19-Community_Transmission-2020.1-eng.pdf

56. Pan D, Sze S, Minhas JS, Bangash MN, Pareek N, Divall P, et al. The impact of ethnicity on clinical outcomes in COVID-19: A systematic review. EClinicalMedicine. 2020 Jun;100404.

57. Holmes L, Enwere M, Williams J, Ogundele B, Chavan P, Piccoli T, et al. Black–White Risk Differentials in COVID-19 (SARS-COV2) Transmission, Mortality and Case Fatality in the United States: Translational Epidemiologic Perspective and Challenges. Int J Environ Res Public Health. 2020 Jun 17;17(12):4322.

58. Ebor MT, Loeb TB, Trejo L. Social Workers Must Address Intersecting Vulnerabilities among Noninstitutionalized, Black, Latinx, and Older Adults of Color during the COVID-19 Pandemic. J Gerontol Soc Work. 2020 Jun 19;1–4.

59. Bodenmann P, Pahud-Vermeulen B, Bouche L, Sanchis Zozaya J, Bauermeister M, Berzig A. Left behind populations, COVID-19 and risks of health inequities : a guide of the local social-health network (Vaud, Switzerland). Rev Med Suisse. 2020 Apr 29;16(N° 691-2):859–62.

60. Barnett K, Mercer SW, Norbury M, Watt G, Wyke S, Guthrie B. Epidemiology of multimorbidity and implications for health care, research, and medical education: a cross-sectional study. The Lancet. 2012 Jul;380(9836):37–43.

61. McCormick B, Hill P-S, Redding S. Comparative morbidities and the share of emergencies in hospital admissions in deprived areas: a method and evidence from English administrative data. BMJ Open. 2018 Aug;8(8):e022573.

62. Gavin M, Esmail A. Solving the Recruitment Crisis in UK General Practice: Time to Consider Physician Assistants? Soc Policy Adm. 2002 Feb;36(1):76–89.

63. Ministry of Housing, Community & Local Government. The English Indices of Deprivation 2019 [Internet]. 2019. Available from: https://assets.publishing.service.gov.uk/government/uploads/system/uploads/attachment_data/file/835115/IoD2019_Statistical_Release.pdf

For peer review only

64. Anselin L. Local Indicators of Spatial Association-LISA. Geogr Anal. 2010 Sep 3;27(2):93–115.

Contributorship: CN and NC developed the original concept, conducted the analysis and prepared the article draft. SB and FM contributed to the empirical analysis and to drafting the article. DSL, MS and SR contributed to the concept of the paper and to writing the article. CN, SR and MS led the data collection. CN, SR and NC led the literature review (research in context).

Declaration of interests: No competing interests. All authors have completed the ICMJE uniform disclosure form at www.icmje.org/coi_disclosure.pdf and declare: no support from any organisation for the submitted work; no financial relationships with any organisations that might have an interest in the submitted work in the previous three years; no other relationships or activities that could appear to have influenced the submitted work.

Funding: This study was not directly funded. CN and DSL are supported by the National Institute for Health Research (NIHR) Applied Research Collaboration (ARC) West Midlands. The views expressed are those of the authors and not necessarily those of the NIHR or Department of Health and Social Care.

Data sharing agreement: All data used in this project is freely available from NHS Digital.

For peer review only

Figure 1: Synthetic Indicator of Vulnerability at Clinical Commissioning Groups (CCGs) level in England

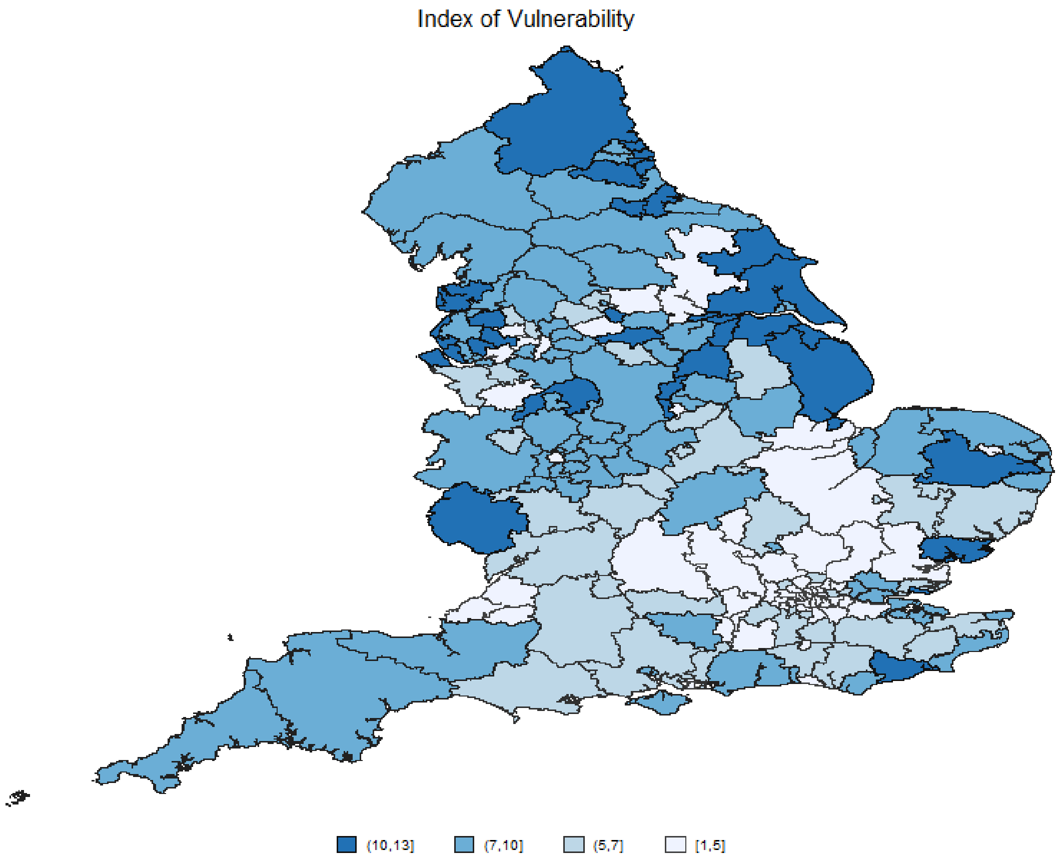

Source: Own elaboration based on NHS digital data at CCGs areas 2019.

For peer review only - http://bmjopen.bmj.com/site/about/guidelines.xhtml

Figure 2: **Correlation between Indicator of Vulnerability and Deaths due to COVID-19 at CCG areas**

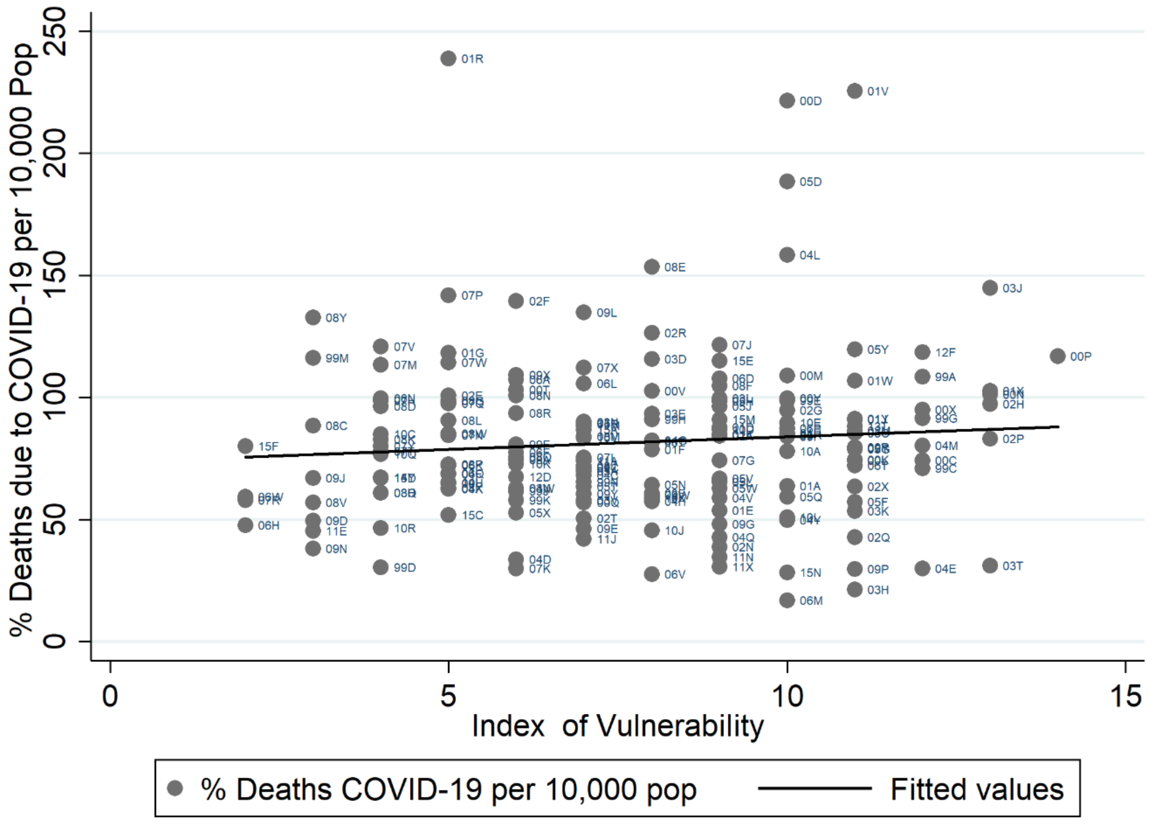

Source: Own elaboration based on NHS digital data at CCGs areas 2019 and the deaths registered data until 22 weeks provided by ONS 2020.

ew only

Figure 3: Correlation between Indicator of Vulnerability and Index of Multiple Deprivation rank (IMD)

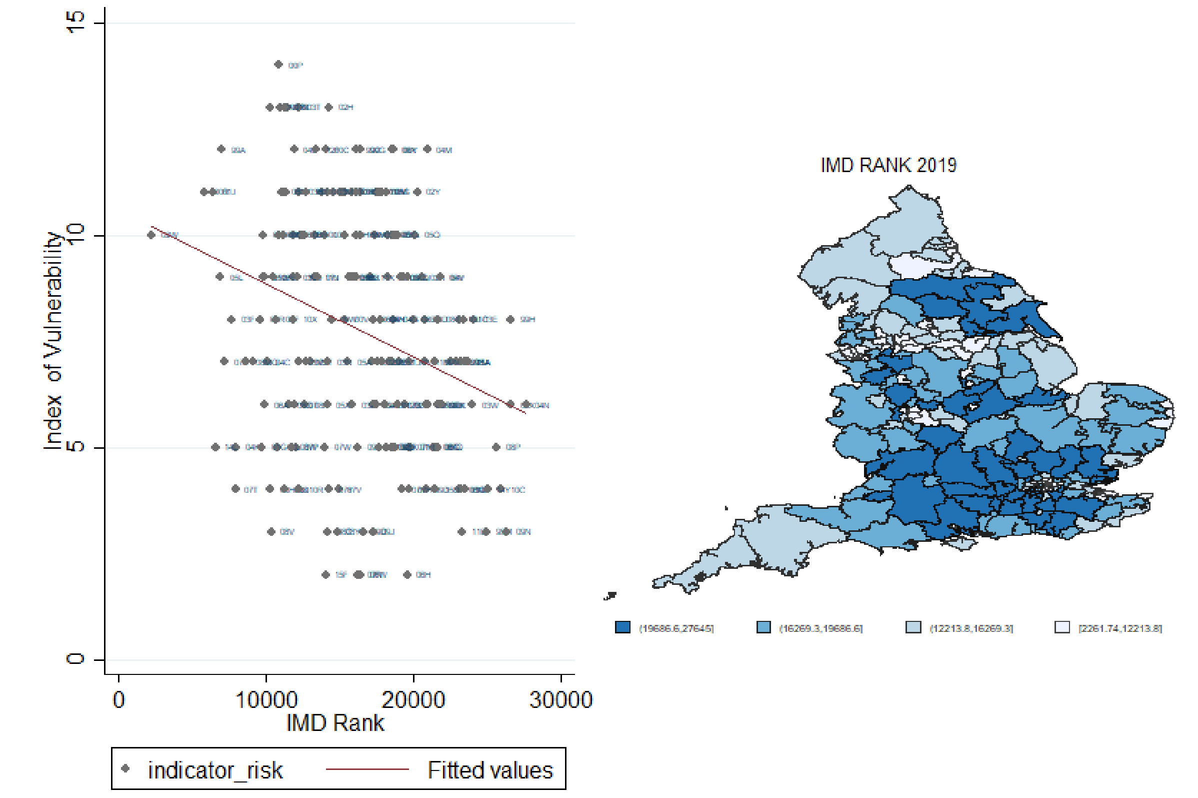

Source: Own elaboration based on NHS digital data at CCGs areas 2019.

Supplementary file

Table S1: Descriptive Statistics

	Mean	Sd	Min	Max	# Obs
Nurses per 10,000 population	3.05	0.87	1.15	6.4	191
GPs per 10,000 population	5.98	0.88	3.81	9.26	191
NHS Hospital staff per 10,000 population	191.92	236.18	0	1592.15	191
IMD CCG Rank	16242.3	4927.95	2261.74	27645	191
COPD prevalence per 10,000 patients	201.32	66.28	79.52	406.13	191
Cancer prevalence per 10,000 patients	306.04	83.35	67.83	593.81	191
CKD prevalence per 10,000 patients	336.75	114.19	108.96	814.88	191
Diabetes prevalence per 10,000 patients	569.58	107.99	241.18	1090.75	191
Hypertension prevalence per 10,000 patients	1427.8	241.87	738.36	1857.47	191
Cardiovascular disease prevalence per 10,000 patients	137.8	35.94	56.69	216.38	191
Proportion aged 70+ per 10,000 patients	38706	27289	3845	207788	191
Ratio of residential care homes per 10,000 population aged 70+	21.11	7.15	1.52	44.73	191
Indicator of unplanned admissions for chronic disease	924.97	272.18	170.3	1666.9	191
Indicator of hospital readmission within 30 days	14.22	1.09	11.6	17.6	184
Indicator of mortality within 30 days of discharge	403.6	67.64	211.27	573.09	184
Indicator of acute emergency admission	1427.97	376.18	243.4	2305.8	191
Rural/Urban GGC Indicator	4.17	1.57	1	6	191

For peer review only

Figure 1: Synthetic Indicator of Vulnerability at Clinical Commissioning Groups (CCGs) level in England

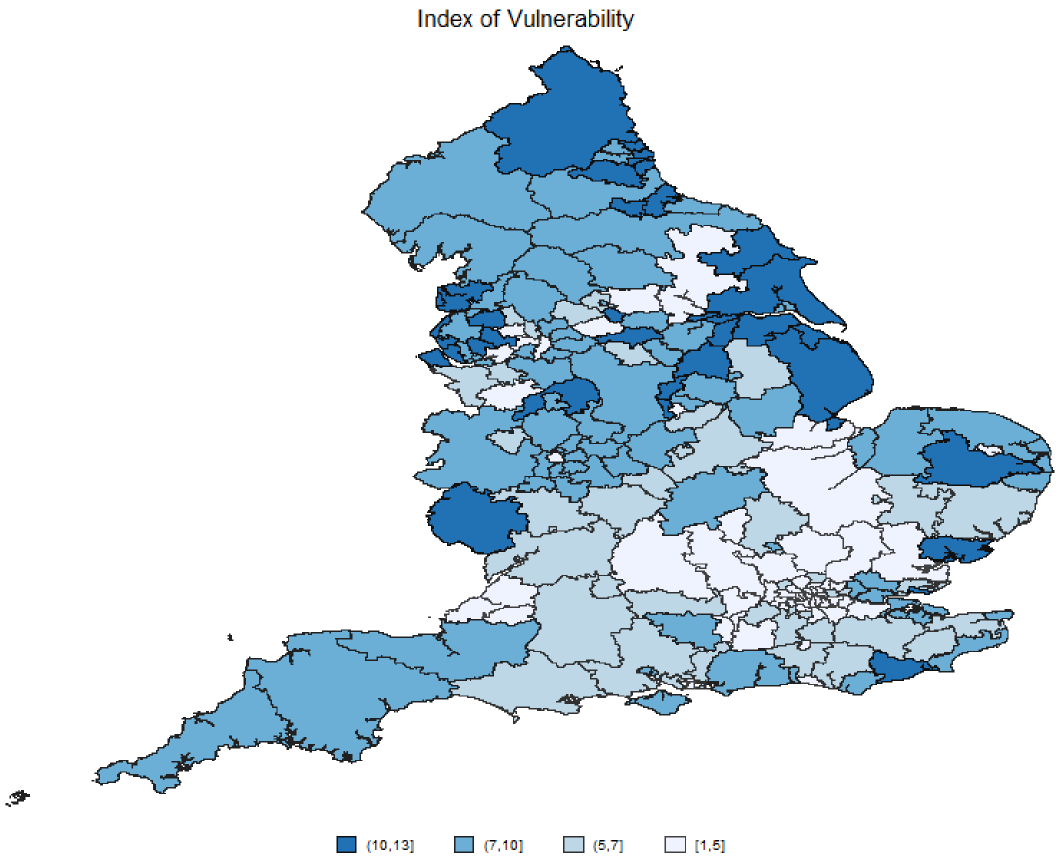

Source: Own elaboration based on NHS digital data at CCGs areas 2019.

Figure S2: Prevalence diseases distribution across CCGs areas

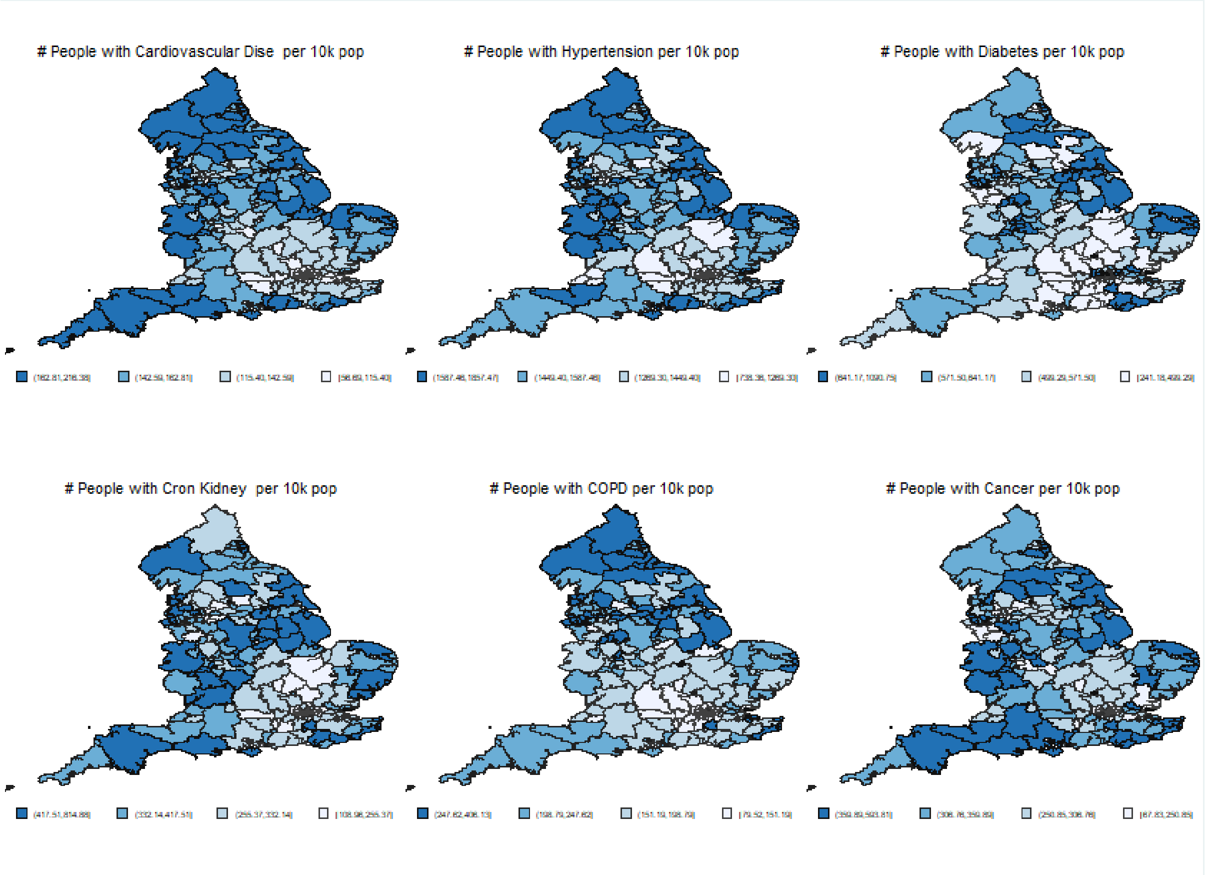

Source: Own elaboration based on NHS digital data at CCGs areas 2019.

For peer review only - http://bmjopen.bmj.com/site/about/guidelines.xhtml

Figure S3: Workforce employed by the National Health System at CCGs level

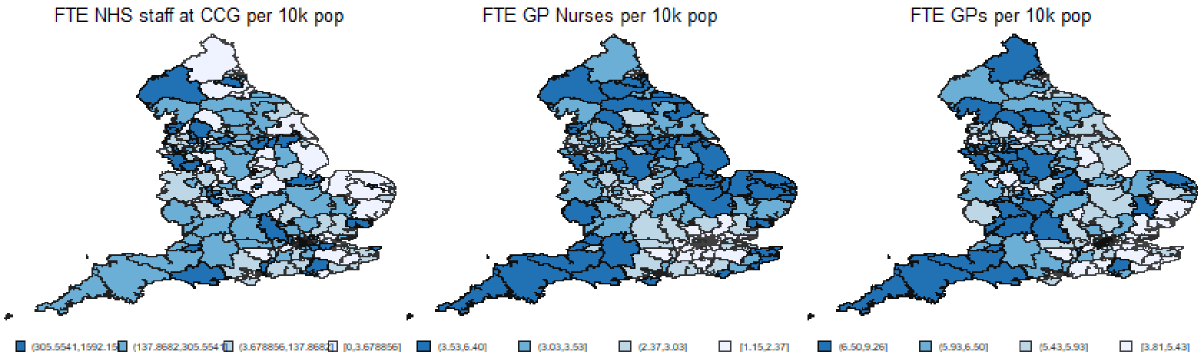

Source: Own elaboration based on NHS digital data at CCGs areas 2019.

Figure S4: Quality of Healthcare Indicators at CCGs level

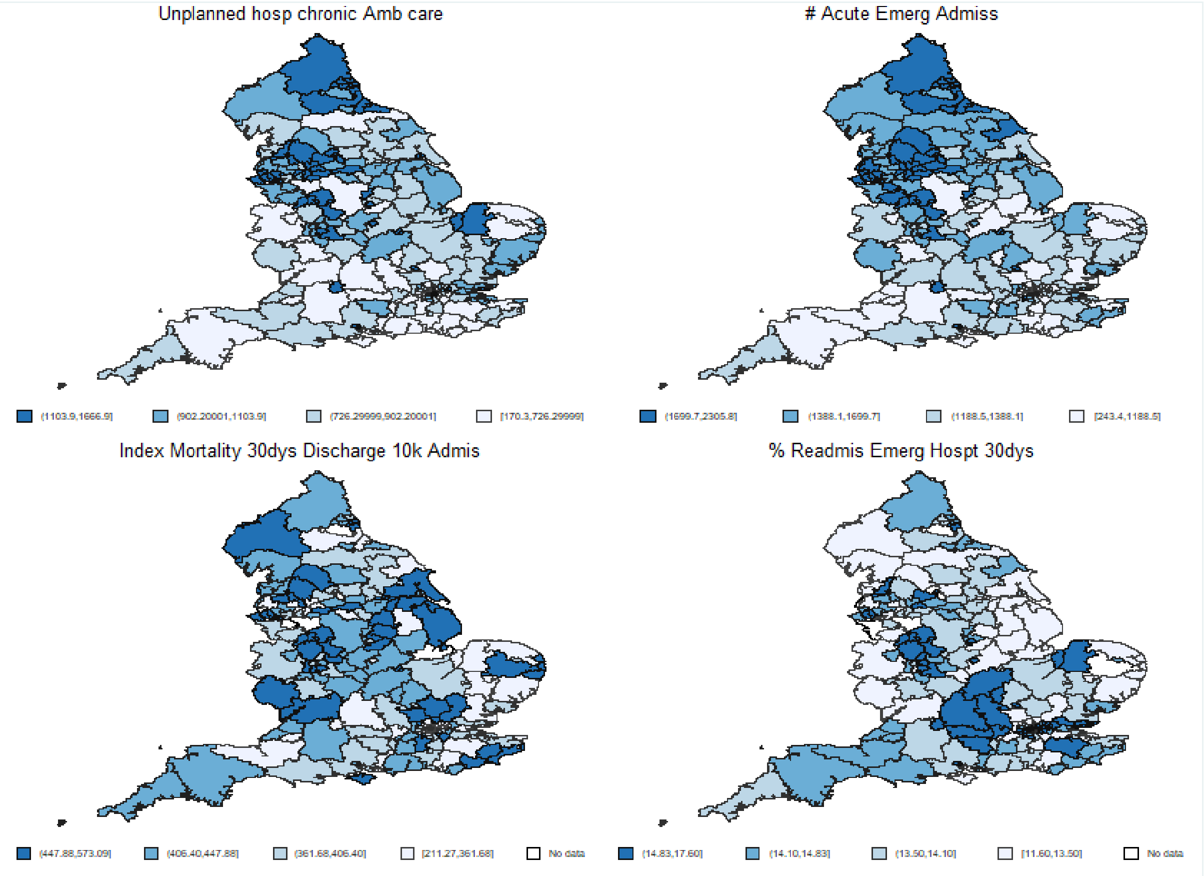

Source: Own elaboration based on NHS digital data at CCGs areas.

Figure S5: **: Quartile-based Index of Vulnerability at CCGs level**

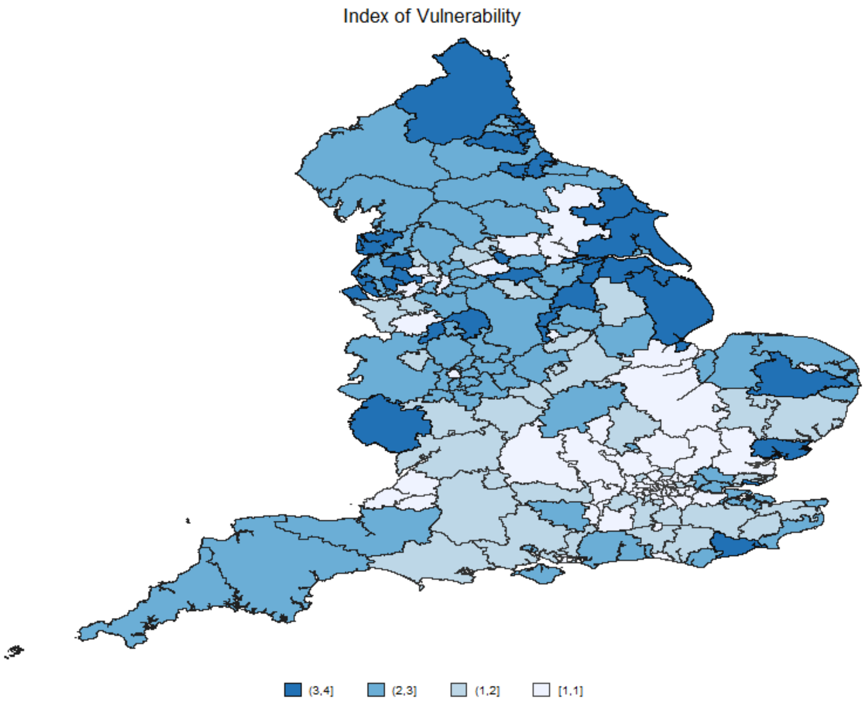

Source: Own elaboration based on NHS digital data at CCGs areas.

For peer review only

STROBE Statement—checklist of items that should be included in reports of observational studies

	Item No	Recommendation	Page No
**Title and abstract**	1	(*a*) Indicate the study's design with a commonly used term in the title or the abstract	1
		(*b*) Provide in the abstract an informative and balanced summary of what was done and what was found	2
**Introduction**			
Background/rationale	2	Explain the scientific background and rationale for the investigation being reported	3
Objectives	3	State specific objectives, including any prespecified hypotheses	3
**Methods**			
Study design	4	Present key elements of study design early in the paper	3
Setting	5	Describe the setting, locations, and relevant dates, including periods of recruitment, exposure, follow-up, and data collection	3
Participants	6	(*a*) *Cohort study*—Give the eligibility criteria, and the sources and methods of selection of participants. Describe methods of follow-up<br>*Case-control study*—Give the eligibility criteria, and the sources and methods of case ascertainment and control selection. Give the rationale for the choice of cases and controls<br>*Cross-sectional study*—Give the eligibility criteria, and the sources and methods of selection of participants	3
		(*b*) *Cohort study*—For matched studies, give matching criteria and number of exposed and unexposed<br>*Case-control study*—For matched studies, give matching criteria and the number of controls per case	NA
Variables	7	Clearly define all outcomes, exposures, predictors, potential confounders, and effect modifiers. Give diagnostic criteria, if applicable	3-4
Data sources/ measurement	8*	For each variable of interest, give sources of data and details of methods of assessment (measurement). Describe comparability of assessment methods if there is more than one group	3-4
Bias	9	Describe any efforts to address potential sources of bias	NA
Study size	10	Explain how the study size was arrived at	NA
Quantitative variables	11	Explain how quantitative variables were handled in the analyses. If applicable, describe which groupings were chosen and why	NA
Statistical methods	12	(*a*) Describe all statistical methods, including those used to control for confounding	4-7
		(*b*) Describe any methods used to examine subgroups and interactions	NA
		(*c*) Explain how missing data were addressed	NA
		(*d*) *Cohort study*—If applicable, explain how loss to follow-up was addressed<br>*Case-control study*—If applicable, explain how matching of cases and controls was addressed<br>*Cross-sectional study*—If applicable, describe analytical methods taking account of sampling strategy	NA
		(*e*) Describe any sensitivity analyses	4-5

Continued on next page

For peer review only - http://bmjopen.bmj.com/site/about/guidelines.xhtml

**Results**			
Participants	13*	(a) Report numbers of individuals at each stage of study—eg numbers potentially eligible, examined for eligibility, confirmed eligible, included in the study, completing follow-up, and analysed	NA
		(b) Give reasons for non-participation at each stage	NA
		(c) Consider use of a flow diagram	NA
Descriptive data	14*	(a) Give characteristics of study participants (eg demographic, clinical, social) and information on exposures and potential confounders	12
		(b) Indicate number of participants with missing data for each variable of interest	12
		(c) *Cohort study*—Summarise follow-up time (eg, average and total amount)	NA
Outcome data	15*	*Cohort study*—Report numbers of outcome events or summary measures over time	NA
		Case-control study—Report numbers in each exposure category, or summary measures of exposure	NA
		Cross-sectional study—Report numbers of outcome events or summary measures	NA
Main results	16	(*a*) Give unadjusted estimates and, if applicable, confounder-adjusted estimates and their precision (eg, 95% confidence interval). Make clear which confounders were adjusted for and why they were included	12-13
		(*b*) Report category boundaries when continuous variables were categorized	NA
		(*c*) If relevant, consider translating estimates of relative risk into absolute risk for a meaningful time period	NA
Other analyses	17	Report other analyses done—eg analyses of subgroups and interactions, and sensitivity analyses	4-5
**Discussion**			
Key results	18	Summarise key results with reference to study objectives	10
Limitations	19	Discuss limitations of the study, taking into account sources of potential bias or imprecision. Discuss both direction and magnitude of any potential bias	9-10
Interpretation	20	Give a cautious overall interpretation of results considering objectives, limitations, multiplicity of analyses, results from similar studies, and other relevant evidence	10
Generalisability	21	Discuss the generalisability (external validity) of the study results	10
**Other information**			
Funding	22	Give the source of funding and the role of the funders for the present study and, if applicable, for the original study on which the present article is based	11

*Give information separately for cases and controls in case-control studies and, if applicable, for exposed and unexposed groups in cohort and cross-sectional studies.

Note: An Explanation and Elaboration article discusses each checklist item and gives methodological background and published examples of transparent reporting. The STROBE checklist is best used in conjunction with this article (freely available on the Web sites of PLoS Medicine at http://www.plosmedicine.org/, Annals of Internal Medicine at http://www.annals.org/, and Epidemiology at http://www.epidem.com/). Information on the STROBE Initiative is available at www.strobe-statement.org.

For peer review only

BMJ Open

Measuring geographical disparities in England at the time of COVID-19: results using a composite indicator of population vulnerability

Journal:	*BMJ Open*
Manuscript ID	bmjopen-2020-039749.R2
Article Type:	Original research
Date Submitted by the Author:	21-Jul-2020
Complete List of Authors:	Nicodemo, Catia; Oxford University, Nuffield Primary Care ; CHSEO, University of Oxford<br>Barzin, Samira; Oxford University<br>Lasserson, Daniel; University of Birmingham, Institute of Applied Health Research<br>Moscone, F; Brunel University,<br>Redding, Stuart; Nuffield Department of Primary Care Health Sciences<br>Shaikh, Mujaheed; Hertie School gGmbH<br>Cavalli, Nicolo'; University of Oxford Nuffield College
**Primary Subject Heading**:	Health policy
Secondary Subject Heading:	Health economics, Infectious diseases, Public health, Sociology
Keywords:	Health policy < HEALTH SERVICES ADMINISTRATION & MANAGEMENT, HEALTH ECONOMICS, Adult intensive & critical care < INTENSIVE & CRITICAL CARE

SCHOLARONE™
Manuscripts

I, the Submitting Author has the right to grant and does grant on behalf of all authors of the Work (as defined in the below author licence), an exclusive licence and/or a non-exclusive licence for contributions from authors who are: i) UK Crown employees; ii) where BMJ has agreed a CC-BY licence shall apply, and/or iii) in accordance with the terms applicable for US Federal Government officers or employees acting as part of their official duties; on a worldwide, perpetual, irrevocable, royalty-free basis to BMJ Publishing Group Ltd ("BMJ") its licensees and where the relevant Journal is co-owned by BMJ to the co-owners of the Journal, to publish the Work in this journal and any other BMJ products and to exploit all rights, as set out in our licence.

The Submitting Author accepts and understands that any supply made under these terms is made by BMJ to the Submitting Author unless you are acting as an employee on behalf of your employer or a postgraduate student of an affiliated institution which is paying any applicable article publishing charge ("APC") for Open Access articles. Where the Submitting Author wishes to make the Work available on an Open Access basis (and intends to pay the relevant APC), the terms of reuse of such Open Access shall be governed by a Creative Commons licence – details of these licences and which Creative Commons licence will apply to this Work are set out in our licence referred to above.

Other than as permitted in any relevant BMJ Author's Self Archiving Policies, I confirm this Work has not been accepted for publication elsewhere, is not being considered for publication elsewhere and does not duplicate material already published. I confirm all authors consent to publication of this Work and authorise the granting of this licence.

For peer review only

Measuring geographical disparities in England at the time of COVID-19: results using a composite indicator of population vulnerability

Catia Nicodemo,[1 2 *] Samira Barzin,[3] Daniel S. Lasserson,[4 5] Francesco Moscone, [6 7]

Stuart Redding,[1] Mujaheed Shaikh,[8] Nicolò Cavalli [9 10]

[1] Centre for Health Service Economics & Organisation, Nuffield Department of Primary Care Health Sciences, Oxford University, Woodstock Road, Oxford, OX2 6GG United Kingdom

[2] Department of Economics, Oxford University, Manor Road, Oxford, OX1 3UQ United Kingdom

[3] Mathematical Institute, Oxford University, Woodstock Road, Oxford, OX2 6GG United Kingdom

[4] Institute of Applied Health Research, College of Medical and Dental Sciences, University of Birmingham, Birmingham, B15 255 United Kingdom

[5] Department of Acute Medicine, Sandwell and West Birmingham NHS Trust, City Hospital, Dudley Road, Birmingham, United Kingdom

[6] Brunel University London, Kingston Lane, Uxbridge, UB83PH United Kingdom

[7] Università Ca' Foscari Venezia, Dorsoduro 3246, Venezia 30123, Italy

[8] Hertie School, Friedrichstraße 180, Berlin, 30123 Germany

[9] Nuffield College, Oxford University, 1 New Road, Oxford, OX1 1NF United Kingdom

[10] Carlo F. Dondena Centre for Research on Social Dynamics and Public Policy, Bocconi University, 1 Via Guglielmo Röntgen, Milan, 20136 Italy

[*]Corresponding author: catia.nicodemo@gmail.com, Centre for Health Service Economics and Organisation, Nuffield Department of Primary Care Health Sciences, University of Oxford, Gibson Building, Radcliffe Observatory Quarter, Woodstock Road, Oxford OX2 6GG, Tel+447775158584

For peer review only

Abstract

Objectives – The growth of COVID-19 infections in England raises questions about system vulnerability. Several factors that vary across geographies, such as age, existing disease prevalence, medical resource availability, and deprivation, can trigger adverse effects on the National Health System during a pandemic. In this paper, we present data on these factors and combine them to create an index to show which areas are more exposed. This technique can help policy makers to moderate the impact of similar pandemics.

Design – We combine several sources of data, which describe specific risk factors linked with the outbreak of a respiratory pathogen, that could leave local areas vulnerable to the harmful consequences of large-scale outbreaks of contagious diseases. We combine these measures to generate an index of community-level vulnerability.

Setting – 191 Clinical Commissioning Groups (CCGs) in England.

Main outcome measures – We merge 15 measures spatially to generate an index of community-level vulnerability. These measures cover prevalence rates of high-risk diseases; proxies for the at-risk population density; availability of staff and quality of healthcare facilities.

Results – We find that 80% of CCGs that score in the highest quartile of vulnerability are located in the North of England (24 out of 30). Here, vulnerability stems from a faster rate of population ageing and from the widespread presence of underlying at-risk diseases. These same areas, especially the North-East Coast areas of Lancashire, also appear vulnerable to adverse shocks to healthcare supply due to tighter labour markets for healthcare personnel. Importantly, our Index correlates with a measure of social deprivation, indicating that these communities suffer from long-standing lack of economic opportunities and are characterised by low public and private resource endowments.

Conclusions – Evidence-based policy is crucial to mitigate the health impact of pandemics such as COVID-19. While current attention focuses on curbing rates of contagion, we introduce a vulnerability index combining data that can help policy makers identify the most vulnerable communities. We find that this index is positively correlated with COVID-19 deaths and it can thus be used to guide targeted capacity building. These results suggest that a stronger focus on deprived and vulnerable communities is needed to tackle future threats from emerging and re-emerging infectious disease.

Keywords: Geographical disparity; Pandemic; Emergency preparedness; Index of vulnerability; Health inequalities; Covid-19.

Word count: 3,519

For peer review only

Strengths and limitations of this study

- We provide important information to help identify the communities most vulnerable to harmful effects of COVID-19
- This fills an important gap in the literature, with only a handful of previous studies that show the distribution of underlying risks within national systems
- Judgement was required when deciding which variables to include or omit
- Our methods give equal weight to each variable when creating the index
- CCGs are quite large and using smaller areas may be more appropriate

For peer review only

1 Introduction

The current COVID-19 outbreak is triggering a renewed understanding of health risks and underlying health vulnerabilities.[1] In a pandemic, overlooked vulnerabilities may arise from the social and biological makeup of local communities. Accumulated evidence from emerging and re-emerging infectious diseases, such as SARS, swine influenza, MERS and now COVID-19, indicates that infections requiring critical care, and associated case fatalities, are usually, but not exclusively, concentrated in elderly patients, and in patients suffering from specific comorbidities, such as chronic obstructive pulmonary disease, cardiovascular diseases, diabetes, cancer, chronic kidney disease.[2–5]

Existing evidence further suggests that infectious disease outbreaks might lead to adverse shocks to the supply of healthcare. Healthcare workers are directly exposed to risk of transmission and may suffer from the physical and psychological impact of sudden surges in workloads that come with high case fatalities.[6,7] Lack of resources available to healthcare providers and suboptimal quality of health services in a community represent a further source of vulnerability, which hamper the supply of critical care, puts patients at higher risk of negative health outcomes, and endanger the safety of health workers.[8,9]

Despite the fact that, in epidemic intelligence, local disease maps have been in use at least since seminal work by Seaman and Pascalis-Ouvière, who in 1796-7 employed spot maps to trace cases of yellow fever in New York and Philadelphia,[10,11] interest in how community level variables may moderate the outcomes of infectious diseases was limited before COVID-19, with only a handful of studies reconstructing the distribution of health risks and vulnerabilities across communities in a broad range of settings.[9,11–14] Recent studies have started to address this gap.[15]

In this paper we focus on England, a large part of the United Kingdom which by June 1st 2020 was the third-worst hit country for per capita COVID-19 deaths.[16] We collect administration information at the level of the Clinical Commissioning Groups (CCG), the administrative units that provide National Health System (NHS) services in England, and combine 15 vulnerability indicators in a synthetic Index of Vulnerability.[17] We report geographic vulnerabilities across 191 CCGs and match them with COVID-19 death data up to the 23rd week of 2020. We find a positive relationship between our indicator of vulnerability and the COVID-19 related death rate over the local population, with a correlation coefficient of around 10%.

We also find that vulnerability is not randomly distributed across geographies. Socio-economically deprived areas display higher prevalence of pre-existing health conditions and lower access to healthcare services. This makes deprived communities disproportionately vulnerable to critical infections and case fatalities during an infectious disease outbreak.[18–24] Identifying these vulnerabilities ahead of time may help to shape preparedness and response policies and, during a pandemic, may be key to fairly allocating the stretched resources of a nation's health systems across communities in need.[14,25–27] In Section 2, we describe the methods used to build our Index of

For peer review only

Vulnerability, which we present in Section 3. In Section 4, we discuss our results and conclude.

2 Methods

Using data gathered from NHS Digital and focusing on the geographic level of Clinical Commissioning Group areas for England (CCGs, n=191) in the financial year 2018/2019, we combine area-level indicators of vulnerability in an Index of Vulnerability to obtain a standardised synthetic vulnerability measure for each CCG in England. To select relevant vulnerability indicators, which we combine in our Index, we queried Pubmed for articles containing the terms 'health', ''inequality', 'vulnerability' and 'pandemics', without language or geographic restrictions. Our search returned 105 results: whereas just 62 studies were published from 1993 to 2019, 43 further papers have been published on the COVID-19 pandemic.

These studies identify three macro-categories of factors that drive geographic differences in health vulnerabilities: i) disparities in susceptibility to a disease; ii) disparities in the likelihood of contracting a disease, and iii) disparities in treatment.[28] Factors that drive disparities in susceptibility include the prevalence of certain pre-existing diseases[29–32] and local demographics, such as a community's age structure.[11,29,31,33–37] There is evidence that the elderly have been hit heavily by the COVID-19 pandemic.[5,37,38] Therefore, in building our Index, we consider i) the number of people above 70 years old per 10,000 residents across CCGs.

We also consider the prevalence of patients suffering from ii) cardiovascular diseases; iii) chronic obstructive pulmonary disease (COPD); iv) cancer; v) chronic kidney disease (CKD) for patients over the age of 18; vi) hypertension; and viii) diabetes (patients aged 17 or older). These are identified by Public Health England, the English health authority, as characterised by higher risks of severe illness from SARS-CoV-2.[39] To track existing conditions, we used Quality and Outcomes Framework prevalences (QOF), computed as the number of patients registered in a General Practice with a certain health condition at a given time, divided by the total patients registered at the same GP and time.

Risk factors associated with the likelihood of contracting the disease include the patterns of population density and mobility;[29,36,40–42] whether a community is principally urban or rural.[11,32,43] Thus, we consider ix) an urban to rural indicator among our vulnerability factors. We also include x) the ratio of residential and nursing homes per 10,000 population aged over 70 years old. Although there is evidence that a good long-term care infrastructure could reduce hospital admissions and mortality,[44] during pandemic a higher density of home cares represents a risk factor due to the frailty of their residents and observed difficulties in stopping within-facility transmission.[29,32,45] During the ongoing pandemic in England, it also has been suggested that freeing up strained hospital capacity by discharging older patients into care homes may have exacerbated the spread of the

For peer review only

disease, although the evidence on this is still mixed.[38,46,47] Based on this, in our Index of Vulnerability, we leverage ix) the ratio of residential and nursing homes per 10,000 population aged over 70 years old; x) an urban to rural indicator.

While variables i)-x) measure quantities that affect the 'demand' of healthcare during a pandemic, extant literature also emphasises the relevance of a set of 'supply'-side factors. Among these, resources available to local healthcare systems, such as the number of intensive care beds and the size of the healthcare workforce, are considered to shape the capacity of local systems to absorb surges in demand.[31,33,40,48,49] The overall quality of a local health system also matters, as high quality practices shield inpatients from the downside risks of infections, and better protect their healthcare workforce.[29]

To build our Index of Vulnerability, we focus on indicators capturing the NHS workforce in each CCG, and on a set of proxies for the quality of healthcare supply. To measure NHS workforce, we measure (xi) the number of nurses within General Practices, general practitioners (GPs) and hospital staff, including non-medical personnel. We consider full-time equivalent equivalents (FTEs), which is a more accurate measure of workforce availability compared to raw headcounts, especially in more deprived and rural areas, where GPs frequently work part-time. We weighted each of these parameters by the CCG-area population. However, while GPs and practice nurses are measured directly at the CCG level, NHS hospital staff is measured at NHS Hospital Trust level. We used the provider code, and the postcode to link the NHS Trusts to the appropriate CCG area.

Following previous literature,[50] we measure quality by including in our Index xii) the percentage of emergency hospital admissions occurring within 30 days of the most recent discharge; xiii) the ratio of deaths within 30 days of a non-elective hospital procedure in 2014 (latest data point available); xiv) the prevalence of unplanned admissions for ambulatory care sensitive conditions; and xv) emergency admissions for acute conditions that should not usually require hospital admission. Measures xiv) and xv) capture acute hospitalisations in patients with specific long-term conditions, when primary care and the delivery of appropriate hospital outpatient services could have prevented deterioration and avoid hospitalization.[51] All the indicators of quality are indirectly standardised rates based on age and sex specific rates in England.

We combine these 15 indicators in a synthetic index of area-level vulnerability (see Table S1 in the supplementary file for descriptive statistics of all indicators used in this study). To build this index, we first dichotomize each variable by comparing it to the mean of the series in the whole of England. For each variable, value "one" (more risk) was assigned to CCGs with higher than average target diseases, higher than average elderly population; to CCGs in mostly urban areas; with higher than average prevalence of home and nursing care facilities; lower-than-average healthcare personnel FTEs; and lower than average hospital quality. We assigned value "zero" for all indicators below the mean.

Building on OECD guidelines,[52] we obtained a synthetic Index of Vulnerability as the arithmetic sum of these dummies for each CCG area (*min*=0; *max*=15, *mean*=7.38).

For peer review only

Therefore, our index represents a functional transformation that, for each CCG area, maps the vector of dichotomous indicators into an integer number. Above-or-below mean indices are a class of additive aggregation methods considered to be robust to outliers. However, they suffer from substantial information loss on the relative distance of each CCG from the national average. We address this issue by presenting a quartile-based indicator in the Supplementary file, which offers similar results to the above-or-below average index and thus provide support for our chosen quantification approach.

Patient and Public Involvement – patients and the public were not involved in this research. We would expect that the information provided here can help encourage a public debate when considering what other variables may deserve inclusion in similar indices in the future.

3 Results

3.1 Spatial analysis of vulnerabilities in England's CCGs

In the supplementary file Figures S1-S4, we plot maps for each of the 15 indicators described in the Methods Section above. We show that, in England, residential and nursing facilities are concentrated in the North and the South West of England, areas also more populated by the elderly. However, looking at the ratio of homecare facilities over the older population, we observe that areas in the North and East Coast have the lowest ratios (Figure S1). Figure S2 depicts the CCG-level distribution of population prevalence for six diseases chosen with the aim of assessing the susceptibility of the system to healthcare demand shocks from the spread of COVID-19.

We find that the areas with the largest prevalence of these underlying diseases, especially cardiovascular diseases, hypertension and chronic kidney disease, are again located in the North and South West of the country. CCGs in the East of the country, including the East Midlands as well as the Yorkshire and Humber areas are particularly affected by diabetes, COPD and cancer, implying a higher underlying health risk for the population living in these areas, compared to the English average. Increases in the demand for critical care driven by the interaction within infectious diseases and underlying comorbidities raise the question of whether the existing supply of healthcare is adequate, or whether capacity building should be considered.

In Figure S3 in the supplementary file, we report on the availability of GPs, nurses in general practices and medical personnel in hospitals. We observe that the healthcare workers are not equally spatially distributed across different areas in England. GPs are more concentrated in the North, the Midlands, and the South West. GP Nurses are spread more evenly across the country, and levels of hospital staff are higher in the South and South East, with the lowest values observed in the coastal areas of the East Midlands and the North East of England.

For peer review only

Finally, in Figure S4, we plot four indicators of hospital quality. We show that there is high variation in these quality markers across England. The North East and North West region of the country (North Kirklees and South Sefton CCGs, for example), report a high number of unplanned hospitalisations, acute emergency admissions and a higher rate of deaths after discharge. High mortality rates appear to be spread more widely, with high levels not only in the North but also in the South (e.g. Sussex and Essex CCGs), and especially in the areas of the South West (Devon CCG).

3.2 A synthetic Index of Vulnerability for England

In Figure 1, we map the synthetic Index of Vulnerability, obtained with the procedure described in the Methods Section 2. According to this Index, CCGs in the North and the South of England appear most vulnerable to an infectious disease outbreak, with Sunderland, East and North Lincolnshire East, and Wigan Borough among the most vulnerable. Table 1 reports the list of all CCGs by vulnerability score, divided in quartiles. In the first quartile (Q1), we find CCG areas with lower Index of Vulnerability and in Q4 the CCGs area with the highest values. These last group of CCGs should be targeted by the NHS to improve their outcomes and increase the measures of protection in case of pandemic. Figure S5 in the supplementary file maps the inter-quartile index of vulnerability at CCG level, with comparable findings.

[FIGURE 1 HERE]

Table 1: CCGs by quantile Index of Vulnerability

Quartile	CCGs
**Q1**	Barnet; Bath and North East Somerset; Bexley; Brent; Brighton and Hove; Bristol, North Somerset and South Gloucestershire; Bromley; Buckinghamshire; Cambridgeshire and Peterborough; Camden; Castle Point and Rochford; Central London (Westminster); City and Hackney; Crawley; Croydon; Dartford, Gravesham and Swanley; Ealing; East Berkshire; East and North Hertfordshire; Fareham and Gosport; Greenwich; Guildford and Waverley; Hammersmith and Fulham; Haringey; Herts Valleys; Hillingdon; Horsham and Mid Sussex; Hounslow; Islington; Kingston; Lambeth; Leeds; Lewisham; Merton; Mid Essex; Milton Keynes; North East Hampshire; Norwich; Nottingham City; Oxfordshire; Portsmouth; Redbridge; Richmond; South Lincolnshire; Surrey Heath; Tower Hamlets; Vale of York; Waltham Forest; Wandsworth; Warrington; West Cheshire; West Essex; West Kent; West London.

For peer review only

**Q2**	Ashford; Barking and Dagenham; Bedfordshire; Berkshire West; Bolton; Bradford District; Bury; Calderdale; Canterbury and Coastal; Dorset; East Leicestershire and Rutland; East Surrey; Eastern Cheshire; Enfield; Gloucestershire; Greater Huddersfield; Hambleton, Richmondshire and Whitby; Harrogate and Rural District; Harrow; Havering; High Weald Lewes Havens; Ipswich and East Suffolk; Leicester City; Lincolnshire West; Manchester; Nene; Newark and Sherwood; Newham; North Hampshire; North Norfolk; North West Surrey; Nottingham North and East; Redditch and Bromsgrove; Rushcliffe; Salford; Sheffield; Shropshire; South Cheshire; South Eastern Hampshire; South Warwickshire; South Worcestershire; Southwark; Stafford and Surrounds; Surrey Downs; Sutton; Swindon; Telford and Wrekin; Vale Royal; West Hampshire; West Suffolk; Wiltshire; Wolverhampton.
**Q3**	Airedale, Wharfedale and Craven; Basildon and Brentwood; Birmingham and Solihull; Blackburn with Darwen; Bradford City; Cannock Chase; Coastal West Sussex; Corby; Coventry and Rugby; Derby and Derbyshire; Devon; Durham Dales, Easington and Sedgefield; East Riding of Yorkshire; East Staffordshire; Eastbourne, Hailsham and Seaford; Fylde and Wyre; Greater Preston; Halton; Hartlepool and Stockton-on-Tees; Hastings and Rother; Herefordshire; Heywood, Middleton and Rochdale; Hull; Isle of Wight; Kernow; Knowsley; Luton; Medway; Morecambe Bay; North Staffordshire; North Tyneside; Northumberland; Nottingham West; Oldham; Rotherham; Sandwell and West Birmingham; Somerset; South East Staffordshire and Seisdon Peninsula; South Kent Coast; South Norfolk; South Tees; South West Lincolnshire; Southampton; Southport and Formby; Stockport; Stoke on Trent; Swale; Thurrock; Trafford; Walsall; Warwickshire North; West Lancashire; West Leicestershire; West Norfolk; Wyre Forest.
**Q4**	Barnsley; Bassetlaw; Blackpool; Chorley and South Ribble; Darlington; Doncaster; Dudley; East Lancashire; Great Yarmouth and Waveney; Lincolnshire East; Liverpool; Mansfield and Ashfield; Newcastle Gateshead; North Cumbria; North Durham; North East Essex; North East Lincolnshire; North Kirklees; North Lincolnshire; Scarborough and Ryedale; South Sefton; South Tyneside; Southend; St Helens; Sunderland; Tameside and Glossop; Thanet; Wakefield; Wigan Borough; Wirral.

Source: Own elaboration based on NHS digital data at CCGs areas 2019.

To test whether CCGs with similar vulnerability levels are more spatially clustered than expected by chance, we computed Moran's I statistic, one of the most widely adopted tests of spatial correlation between observations. Moran's I can be seen as a correlation index that compares statistical units by weighting each pair by a distance function specified by the means of a spatial weight matrix.[53] We have adopted the contiguity criterium to build the spatial weights matrix and standardized it so that the sum of the elements for each row is unity. Results show that Moran's I, calculated on our vulnerability index, is significant, indicating positive spatial correlation (I=0.155, with a p-value of 0.00). This implies that geographically contiguous CCGs tend to show similarly high levels of vulnerability.

For peer review only

3.3 Index of Vulnerability and Deaths to COVID-19

Although this paper was conceived in March 2020, before that the pandemic hit hard in England, we check if the vulnerability index could predict the mortality rate due to COVID-19 across areas up to June 2020. We use the deaths registered data in the first 22 weeks of 2020 across CCG areas from Official National Statistics (ONS), where the numbers of deaths for COVID-19 are reported. In Figure 2, we can observe a positive correlation between mortality rate due to COVID-19 and the Index of Vulnerability. However, some caution needs to be taken when we interpret this result because the data present some limitations. For example, not all deaths have been tested for COVID-19, and this is particularly true for those that happen at home. Most positive tests are recorded in hospitals and this means they are often not reported for the patient's area of residence but rather the CCG in which the hospital is located. These limitations could affect the correlation with the Index of Vulnerability as it is not possible to know accurately the residence of patients with COVID-19.

[FIGURE 2 HERE]

4 Discussion

In this article, we have presented an Index of Vulnerability to map the vulnerability of the English healthcare system to the unexpected and combined consequences of demand- and supply-related pressures associated with infectious disease outbreaks such as the COVID-19 pandemic. To cope with the pandemic, public health authorities across the country have engaged in significant capacity-building efforts, including opening three temporary hospitals that thankfully were not required, but this demonstrates the type of prompt and decisive action that policymakers should be prepared to pursue when faced with pandemics. In order to maximise the effectiveness of these actions, policy makers need evidence such as that presented in this paper so that limited resources are provided to the areas where they are needed the most.

Evidence from previous infectious disease outbreaks, such as Malaria, warns that funding during emergencies tend to be provided in line with health and economic need, but biased towards richer areas.[54] In light of the correlation between CCG-level vulnerability and deprivation reported in this paper, the distribution of resources in times of emergencies should be guided by community-level factors. Guidelines for 'fair allocation of resources' should be developed based on direct knowledge about local communities – not only considering the health impact of such choices, but also long-run economic and social impacts for the community.

For peer review only

Communities and local health care providers should also be involved in these decisions, because community-level knowledge will aid in choosing the right guiding principle – for example, knowledge of population demographics and illness prevalence within a locality might help when deciding whether to maximize benefits/prognosis; to give priority to the worst off; or to reward value to the community. Accurate data collection and dynamic information sharing mechanisms between CCGs – not only of diagnosed cases, but also of staff, medical supplies and excess capacity – will help spread the disease burden and prevent the entire system from collapsing.

No matter what level of community spread has been reached, CCGs should be prepared to act at least in accordance with WHO guidelines.[55] As a minimum, this includes a coordinated strategy across hospitals and communities, and between hospitals and healthcare staff within the community. Planning and stocking medical equipment and drug supplies that are necessary to treat not only COVID-19 patients, but also those with other predisposing illnesses at the local community level is key. Consulting our indicator would allow policymakers and healthcare managers to understand which areas might require strengthened surveillance, monitoring and capacity building so as to minimise the negative outcomes of unexpected health shocks.

Attention should however be paid to the limitations of our Index. The main limitation stems from the selection of indicators of vulnerability. Although our choice was driven by literature, idiosyncratic choices and partial knowledge open the possibility of omitting relevant variables. For example, unlike some existing research,[34] we have not included the availability of beds as a factor that generates supply-side pressures. The reason for this choice is that responses to the COVID-19 crisis suggest that the elasticity in the supply of beds is higher than that of the supply of workforce. In fact, while both bed and healthcare personnel can be increased during a crisis, solutions that could feasibly increase the latter (such as speeding up the promotion of trainees or recalling retirees) have suboptimal implications for quality and organisational efficiency. Moreover, healthcare workers are more exposed to infections, which could create staff shortages, although the hard work of practitioners and managers avoided this to happen in England during the pandemic.

Second, an increasing body of evidence reports a higher risk of infection with COVID-19 and higher mortality in individuals belonging to certain disadvantaged ethnic groups.[19,23,24,56–59] However, rather than revealing ethnic and racial disparities, these differences could reflect underlying socio-economic inequalities, in some cases higher prevalences of pre-existing and at risk health conditions. Other indicators, such as indices of deprivation, might be promising to investigate intersectional vulnerabilities to COVID-19. Deprivation may in fact interact with the vulnerabilities contained in our Index. In fact, social deprivation is associated with the timing of the onset of multimorbidities and the prevalence of long-term conditions.[60] Deprivation also matters for the higher usage of emergency care relative to elective care, 30% of which is not explained by the different

For peer review only

case-mix of conditions suffered by patients from a given area.[61] In addition, recruitment of clinicians to deprived areas presents challenges.[62]

In Figure 3, we plot our Index of Vulnerability against the Index of Multiple Deprivation rank (IMD).[63] We show a positive correlation between the two indices. For example, the North East of England shows high levels of both vulnerability and deprivation. Calculating Moran's I local estimation (LISA)[64] for both indices we detect a positive, although weak (about 20%), statistically significant correlation between the two local spatial indices. This shows that there exists a moderate overlap between the clusters detected by the two indices, which we argue indicates that these indices present complementary evidence rather than substitutable evidence. This is more evident when we plot the two indices against the total mortality rate in figure 4. While the index of vulnerability shows a high correlation with deaths, the line of best fit between mortality rates and the IMD is quite flat. This is not a surprise as the IMD captures inequality in England well but is heavily influenced by an array of factors (education, crime, employment, etc), many of which are unlikely to have a major direct role to play in the spread or outcome of a pandemic. Intersectional analysis aimed at evaluating the potentially overlapping role of social deprivation and health factors, such as those employed in our index, is a fruitful area of research as efforts to collect more granular data are mounting. In order to further test the predictive properties of the generated index of vulnerability, we analyse the spatial cross-correlation[65] between the amount of deaths in each CCG and both the index of vulnerability and IMD respectively. This allows us to identify how strongly each of these indexes correlates with the amount of death, and thus how well it identifies high risk CCGs, while allowing space to be continuous by using the spatial information as weighting parameters. The results indicate that our proposed index of vulnerability performs noticeably better in identifying high risk areas than the IMD, which thus provides support for the proposed index, see figure S6.

[FIGURE 3 HERE]

[FIGURE 4 HERE]

References

1. The Lancet. Redefining vulnerability in the era of COVID-19. The Lancet. 2020 Apr;395(10230):1089.

2. Lau EH, Hsiung CA, Cowling BJ, Chen C-H, Ho L-M, Tsang T, et al. A comparative epidemiologic analysis of SARS in Hong Kong, Beijing and Taiwan. BMC Infect Dis. 2010 Dec;10(1):50.

For peer review only

3. Tekin S, Keske S, Alan S, Batirel A, Karakoc C, Tasdelen-Fisgin N, et al. Predictors of fatality in influenza A virus subtype infections among inpatients in the 2015–2016 season. Int J Infect Dis. 2019 Apr;81:6–9.
4. Rivers CM, Majumder MS, Lofgren ET. Risks of Death and Severe Disease in Patients With Middle East Respiratory Syndrome Coronavirus, 2012–2015. Am J Epidemiol. 2016 Sep 15;184(6):460–4.
5. Zhou F, Yu T, Du R, Fan G, Liu Y, Liu Z, et al. Clinical course and risk factors for mortality of adult inpatients with COVID-19 in Wuhan, China: a retrospective cohort study. The Lancet. 2020 Mar;395(10229):1054–62.
6. Nacoti Mirco, Ciocca Andrea, Giupponi Angelo, Brambillasca Pietro, Lussana Federico, Pisano Michele, et al. At the Epicenter of the Covid-19 Pandemic and Humanitarian Crises in Italy: Changing Perspectives on Preparation and Mitigation. N Engl J Med Catal [Internet]. [cited 2020 Mar 26];1(2). Available from: https://doi.org/10.1056/CAT.20.0080
7. Rosenbaum L. Facing Covid-19 in Italy — Ethics, Logistics, and Therapeutics on the Epidemic's Front Line. N Engl J Med. 2020 May 14;382(20):1873–5.
8. Morens DM, Folkers GK, Fauci AS. The challenge of emerging and re-emerging infectious diseases. Nature. 2004 Jul;430(6996):242–9.
9. Mareiniss DP, Hirshon JM, Thibodeau BC. Disaster planning: potential effects of an influenza pandemic on community healthcare resources. Am J Disaster Med. 2009 Jun;4(3):163–71.
10. Shannon GW. Disease mapping and early theories of Yellow Fever. Prof Geogr. 1981 May;33(2):221–7.
11. Timpka T, Eriksson H, Strömgren M, Eriksson O, Ekberg J, Grimvall A, et al. A neighborhood susceptibility index for planning of local physical interventions in response to pandemic influenza outbreaks. AMIA Annu Symp Proc AMIA Symp. 2010 Nov 13;2010:792–6.
12. Bizimana J-P, Twarabamenye E, Kienberger S. Assessing the social vulnerability to malaria in Rwanda. Malar J. 2015 Dec;14(1):2.
13. Hagenlocher M, Delmelle E, Casas I, Kienberger S. Assessing socioeconomic vulnerability to dengue fever in Cali, Colombia: statistical vs expert-based modeling. Int J Health Geogr. 2013;12(1):36.
14. Hayward AC, Fragaszy EB, Bermingham A, Wang L, Copas A, Edmunds WJ, et al. Comparative community burden and severity of seasonal and pandemic influenza:

For peer review only

results of the Flu Watch cohort study. Lancet Respir Med. 2014 Jun;2(6):445–54.

15. Franch-Pardo I, Napoletano BM, Rosete-Verges F, Billa L. Spatial analysis and GIS in the study of COVID-19. A review. Sci Total Environ. 2020 Oct;739:140033.
16. Dong E, Du H, Gardner L. An interactive web-based dashboard to track COVID-19 in real time. Lancet Infect Dis. 2020 Feb;S1473309920301201.
17. Nicodemo C, Barzin S, Lasserson DS, Moscone F, Redding S, Shaikh M, et al. Population Vulnerability to Unexpected Health Shocks: Geographical Disparities in England at the Time of COVID-19. SSRN Electron J [Internet]. 2020 [cited 2020 Jun 21]; Available from: https://www.ssrn.com/abstract=3571524
18. Rutter PD, Mytton OT, Mak M, Donaldson LJ. Socio-economic disparities in mortality due to pandemic influenza in England. Int J Public Health. 2012 Aug;57(4):745–50.
19. Khalatbari-Soltani S, Cumming RG, Delpierre C, Kelly-Irving M. Importance of collecting data on socioeconomic determinants from the early stage of the COVID-19 outbreak onwards. J Epidemiol Community Health. 2020 May 8;jech-2020-214297.
20. Chung RY-N, Dong D, Li MM. Socioeconomic gradient in health and the covid-19 outbreak. BMJ. 2020 Apr 1;m1329.
21. Andayi F, Crepey P, Kieffer A, Salez N, Abdo AA, Carrat F, et al. Determinants of individuals' risks to 2009 pandemic influenza virus infection at household level amongst Djibouti city residents - A CoPanFlu cross-sectional study. Virol J. 2014 Dec;11(1):13.
22. Zhao H, Harris RJ, Ellis J, Pebody RG. Ethnicity, deprivation and mortality due to 2009 pandemic influenza A(H1N1) in England during the 2009/2010 pandemic and the first post-pandemic season. Epidemiol Infect. 2015 Dec;143(16):3375–83.
23. Gray DM, Anyane-Yeboa A, Balzora S, Issaka RB, May FP. COVID-19 and the other pandemic: populations made vulnerable by systemic inequity. Nat Rev Gastroenterol Hepatol [Internet]. 2020 Jun 15 [cited 2020 Jun 21]; Available from: http://www.nature.com/articles/s41575-020-0330-8
24. Dorn A van, Cooney RE, Sabin ML. COVID-19 exacerbating inequalities in the US. The Lancet. 2020 Apr;395(10232):1243–4.
25. Emanuel EJ, Persad G, Upshur R, Thome B, Parker M, Glickman A, et al. Fair Allocation of Scarce Medical Resources in the Time of Covid-19. N Engl J Med. 2020 May 21;382(21):2049–55.
26. Wang Z, Tang K. Combating COVID-19: health equity matters. Nat Med. 2020 Apr;26(4):458–458.
27. Hill AB. The environment and disease: association or causation? Proc R Soc Med. 1965

For peer review only

May;58:295–300.

28. Blumenshine P, Reingold A, Egerter S, Mockenhaupt R, Braveman P, Marks J. Pandemic Influenza Planning in the United States from a Health Disparities Perspective. Emerg Infect Dis. 2008 May;14(5):709–15.
29. Desmet K, Wacziarg R. Understanding Spatial Variation in COVID-19 across the United States [Internet]. Cambridge, MA: National Bureau of Economic Research; 2020 Jun [cited 2020 Jun 20] p. w27329. Report No.: w27329. Available from: http://www.nber.org/papers/w27329.pdf
30. Shigemura J, Ursano RJ, Morganstein JC, Kurosawa M, Benedek DM. Public responses to the novel 2019 coronavirus (2019-nCoV) in Japan: Mental health consequences and target populations. Psychiatry Clin Neurosci. 2020 Apr;74(4):281–2.
31. Renzaho AMN. The Need for the Right Socio-Economic and Cultural Fit in the COVID-19 Response in Sub-Saharan Africa: Examining Demographic, Economic Political, Health, and Socio-Cultural Differentials in COVID-19 Morbidity and Mortality. Int J Environ Res Public Health. 2020 May 15;17(10):3445.
32. Solis J, Franco-Paredes C, Henao-Martínez AF, Krsak M, Zimmer SM. Structural Vulnerability in the United States Revealed in Three Waves of Novel Coronavirus Disease (COVID-19). Am J Trop Med Hyg [Internet]. 2020 May 7 [cited 2020 Jun 21]; Available from: http://www.ajtmh.org/content/journals/10.4269/ajtmh.20-0391
33. Lakhani A. Which Melbourne Metropolitan Areas Are Vulnerable to COVID-19 Based on Age, Disability, and Access to Health Services? Using Spatial Analysis to Identify Service Gaps and Inform Delivery. J Pain Symptom Manage. 2020 Jul;60(1):e41–4.
34. Verhagen MD, Brazel DM, Dowd JB, Kashnitsky I, Mills M. Forecasting spatial, socioeconomic and demographic variation in COVID-19 health care demand in England and Wales [Internet]. Open Science Framework; 2020 Mar [cited 2020 Jun 21]. Available from: https://osf.io/g8s96
35. Dowd JB, Andriano L, Brazel DM, Rotondi V, Block P, Ding X, et al. Demographic science aids in understanding the spread and fatality rates of COVID-19. Proc Natl Acad Sci. 2020 May 5;117(18):9696–8.
36. Santos JPC dos, Siqueira ASP, Praça HLF, Albuquerque HG. Vulnerabilidade a formas graves de COVID-19: uma análise intramunicipal na cidade do Rio de Janeiro, Brasil. Cad Saúde Pública. 2020;36(5):e00075720.
37. Lim W, Liang C, Assantachai P, Auyeung TW, Kang L, Lee W, et al. COVID-19 and older people in Asia: Asian Working Group for Sarcopenia calls to actions. Geriatr

For peer review only

Gerontol Int. 2020 Jun;20(6):547–58.
38. Docherty AB, Harrison EM, Green CA, Hardwick HE, Pius R, Norman L, et al. Features of 16,749 hospitalised UK patients with COVID-19 using the ISARIC WHO Clinical Characterisation Protocol [Internet]. Infectious Diseases (except HIV/AIDS); 2020 Apr [cited 2020 Jun 22]. Available from: http://medrxiv.org/lookup/doi/10.1101/2020.04.23.20076042
39. Public Health England. Guidance on social distancing for everyone in the UK. Updated March 30, 2020 [Internet]. 2020. Available from: https://www.gov.uk/government/publications/covid-19-guidance-on-social-distancing-and-for-vulnerable-people/guidance-on-social-distancing-for-everyone-in-the-uk-and-protecting-older-people-and-vulnerable-adults
40. Ren H, Zhao L, Zhang A, Song L, Liao Y, Lu W, et al. Early forecasting of the potential risk zones of COVID-19 in China's megacities. Sci Total Environ. 2020 Aug;729:138995.
41. Gómez JM, Verdú M. Network theory may explain the vulnerability of medieval human settlements to the Black Death pandemic. Sci Rep. 2017 Apr;7(1):43467.
42. Hintzen PC. Precarity and the HIV/AIDS pandemic in the Caribbean: Structural stigma, constitutionality, legality in development practice. Glob Public Health. 2019 Nov 2;14(11):1624–38.
43. Peters DJ. Community Susceptibility and Resiliency to COVID-19 Across the Rural-Urban Continuum in the United States. J Rural Health. 2020 Jun 16;jrh.12477.
44. Barker I, Steventon A, Deeny SR. Association between continuity of care in general practice and hospital admissions for ambulatory care sensitive conditions: cross sectional study of routinely collected, person level data. BMJ. 2017 Feb 1;j84.
45. McMichael TM, Clark S, Pogosjans S, Kay M, Lewis J, Baer A, et al. COVID-19 in a Long-Term Care Facility — King County, Washington, February 27–March 9, 2020. MMWR Morb Mortal Wkly Rep. 2020 Mar 27;69(12):339–42.
46. National Audit Office. Readying the NHS and adult social care in England for COVID-19 [Internet]. 2020. Available from: https://www.nao.org.uk/wp-content/uploads/2020/06/Readying-the-NHS-and-adult-social-care-in-England-for-COVID-19.pdf
47. NHS Providers. Recent NHS discharghes into care homes [Internet]. 2020. (Coronavirus briefing). Available from: https://nhsproviders.org/media/689548/spotlight-on-recent-discharges-into-care-homes-nhs-providers.pdf

For peer review only

48. Marmot M. Society and the slow burn of inequality. The Lancet. 2020 May;395(10234):1413–4.

49. Kang J-Y, Michels AC, Lyu F, Wang S, Agbodo N, Freeman VL, et al. Rapidly Measuring Spatial Accessibility of COVID-19 Healthcare Resources: A Case Study of Illinois, USA [Internet]. Public and Global Health; 2020 May [cited 2020 Jun 20]. Available from: http://medrxiv.org/lookup/doi/10.1101/2020.05.06.20093534

50. Gutacker N, Siciliani L, Moscelli G, Gravelle H. Choice of hospital: Which type of quality matters? J Health Econ. 2016 Dec;50:230–46.

51. Sundmacher L, Fischbach D, Schuettig W, Naumann C, Augustin U, Faisst C. Which hospitalisations are ambulatory care-sensitive, to what degree, and how could the rates be reduced? Results of a group consensus study in Germany. Health Policy. 2015 Nov;119(11):1415–23.

52. European Commission, Organisation for Economic Co-operation and Development, SourceOECD (Online service), editors. Handbook on constructing composite indicators: methodology and user guide. Paris: OECD; 2008. 158 p.

53. Moran PAP. Notes on Continuous Stochastic Phenomena. Biometrika. 1950 Jun;37(1/2):17.

54. Barrenho E, Miraldo M, Shaikh M, Atun R. Vertical and horizontal equity of funding for malaria control: a global multisource funding analysis for 2006–2010. BMJ Glob Health. 2017 Dec;2(4):e000496.

55. World Health Organisation. Responding to community spread of COVID-19. Interim guidance. [Internet]. 2020. Available from: https://www.dropbox.com/home/covid_uk/literature?preview=WHO-COVID-19-Community_Transmission-2020.1-eng.pdf

56. Pan D, Sze S, Minhas JS, Bangash MN, Pareek N, Divall P, et al. The impact of ethnicity on clinical outcomes in COVID-19: A systematic review. EClinicalMedicine. 2020 Jun;100404.

57. Holmes L, Enwere M, Williams J, Ogundele B, Chavan P, Piccoli T, et al. Black–White Risk Differentials in COVID-19 (SARS-COV2) Transmission, Mortality and Case Fatality in the United States: Translational Epidemiologic Perspective and Challenges. Int J Environ Res Public Health. 2020 Jun 17;17(12):4322.

58. Ebor MT, Loeb TB, Trejo L. Social Workers Must Address Intersecting Vulnerabilities among Noninstitutionalized, Black, Latinx, and Older Adults of Color during the COVID-19 Pandemic. J Gerontol Soc Work. 2020 Jun 19;1–4.

For peer review only

59. Bodenmann P, Pahud-Vermeulen B, Bouche L, Sanchis Zozaya J, Bauermeister M, Berzig A. Left behind populations, COVID-19 and risks of health inequities : a guide of the local social-health network (Vaud, Switzerland). Rev Med Suisse. 2020 Apr 29;16(N° 691-2):859–62.
60. Barnett K, Mercer SW, Norbury M, Watt G, Wyke S, Guthrie B. Epidemiology of multimorbidity and implications for health care, research, and medical education: a cross-sectional study. The Lancet. 2012 Jul;380(9836):37–43.
61. McCormick B, Hill P-S, Redding S. Comparative morbidities and the share of emergencies in hospital admissions in deprived areas: a method and evidence from English administrative data. BMJ Open. 2018 Aug;8(8):e022573.
62. Gavin M, Esmail A. Solving the Recruitment Crisis in UK General Practice: Time to Consider Physician Assistants? Soc Policy Adm. 2002 Feb;36(1):76–89.
63. Ministry of Housing, Community & Local Government. The English Indices of Deprivation 2019 [Internet]. 2019. Available from: https://assets.publishing.service.gov.uk/government/uploads/system/uploads/attachment_data/file/835115/IoD2019_Statistical_Release.pdf
64. Anselin L. Local Indicators of Spatial Association-LISA. Geogr Anal. 2010 Sep 3;27(2):93–115.
65. Chen, Yanguang. A New Methodology of Spatial-Cross Correlation Analysis. PLOS One 10(5). 2015.

For peer review only

Contributorship: CN and NC developed the original concept, conducted the analysis and prepared the article draft. SB and FM contributed to the empirical analysis and to drafting the article. DSL, MS and SR contributed to the concept of the paper and to writing the article. CN, SR and MS led the data collection. CN, SR and NC led the literature review (research in context).

Declaration of interests: No competing interests. All authors have completed the ICMJE uniform disclosure form at www.icmje.org/coi_disclosure.pdf and declare: no support

from any organisation for the submitted work; no financial relationships with any organisations that might have an interest in the submitted work in the previous three years; no other relationships or activities that could appear to have influenced the submitted work.

Funding: This study was not directly funded. CN and DSL are supported by the National Institute for Health Research (NIHR) Applied Research Collaboration (ARC) West Midlands. The views expressed are those of the authors and not necessarily those of the NIHR or Department of Health and Social Care.

Data sharing agreement: no additional data available

For peer review only

Figure 1: Synthetic Indicator of Vulnerability at Clinical Commissioning Groups (CCGs) level in England

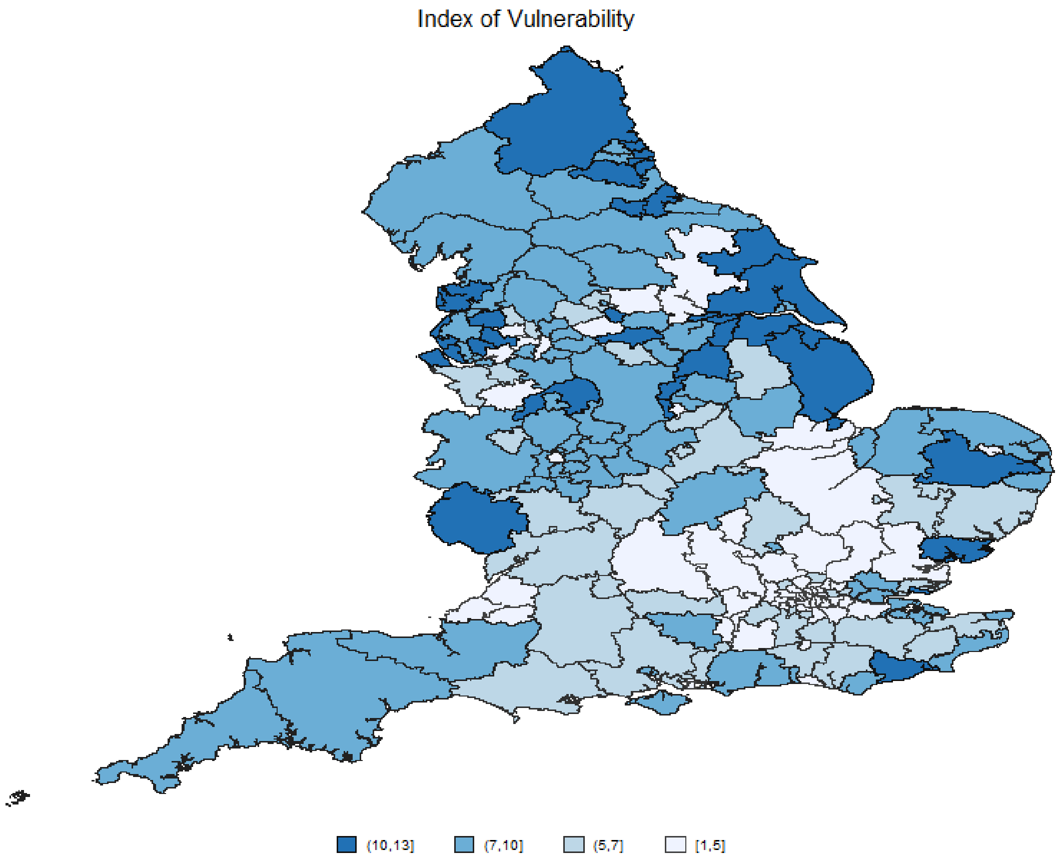

Source: Own elaboration based on NHS digital data at CCGs areas 2019.

Figure 2: Correlation between Indicator of Vulnerability and Deaths due to COVID-19 at CCG areas

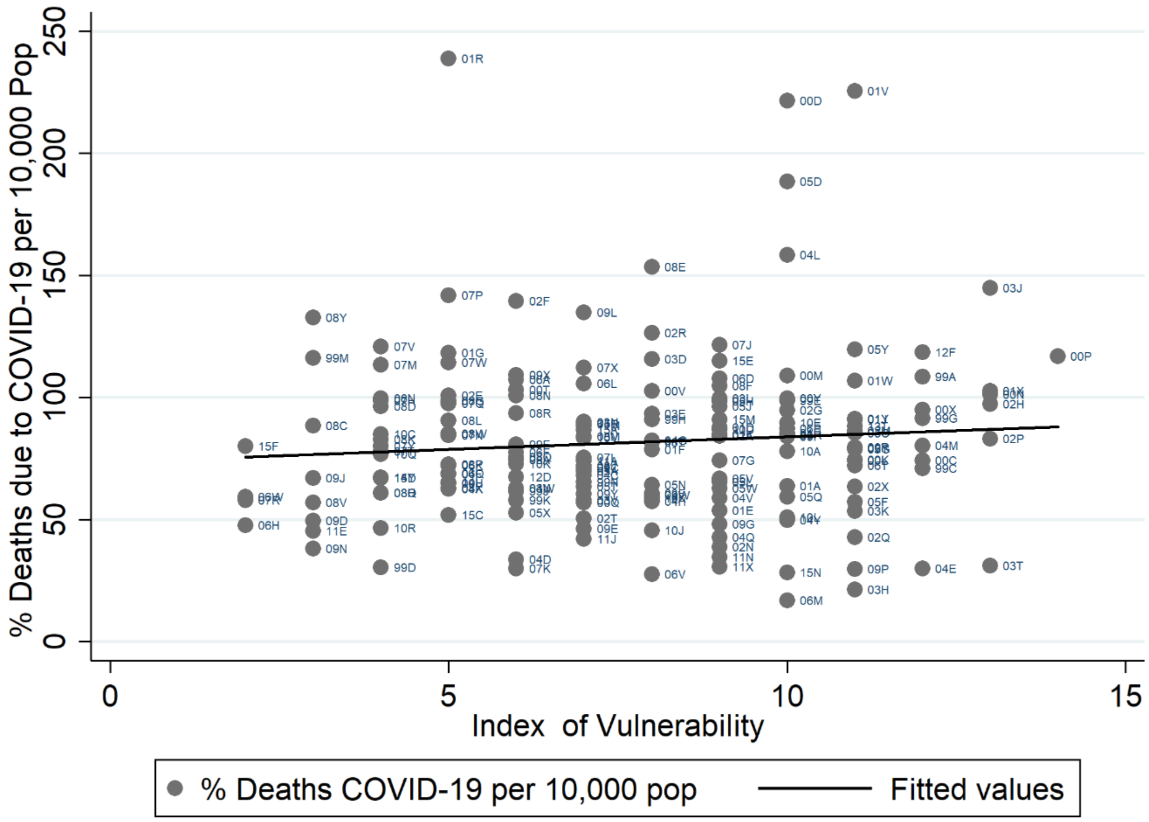

Source: Own elaboration based on NHS digital data at CCGs areas 2019 and the deaths registered data until 22 weeks provided by ONS 2020.

For peer review only

Figure 3: Correlation between Indicator of Vulnerability and Index of Multiple Deprivation rank (IMD)

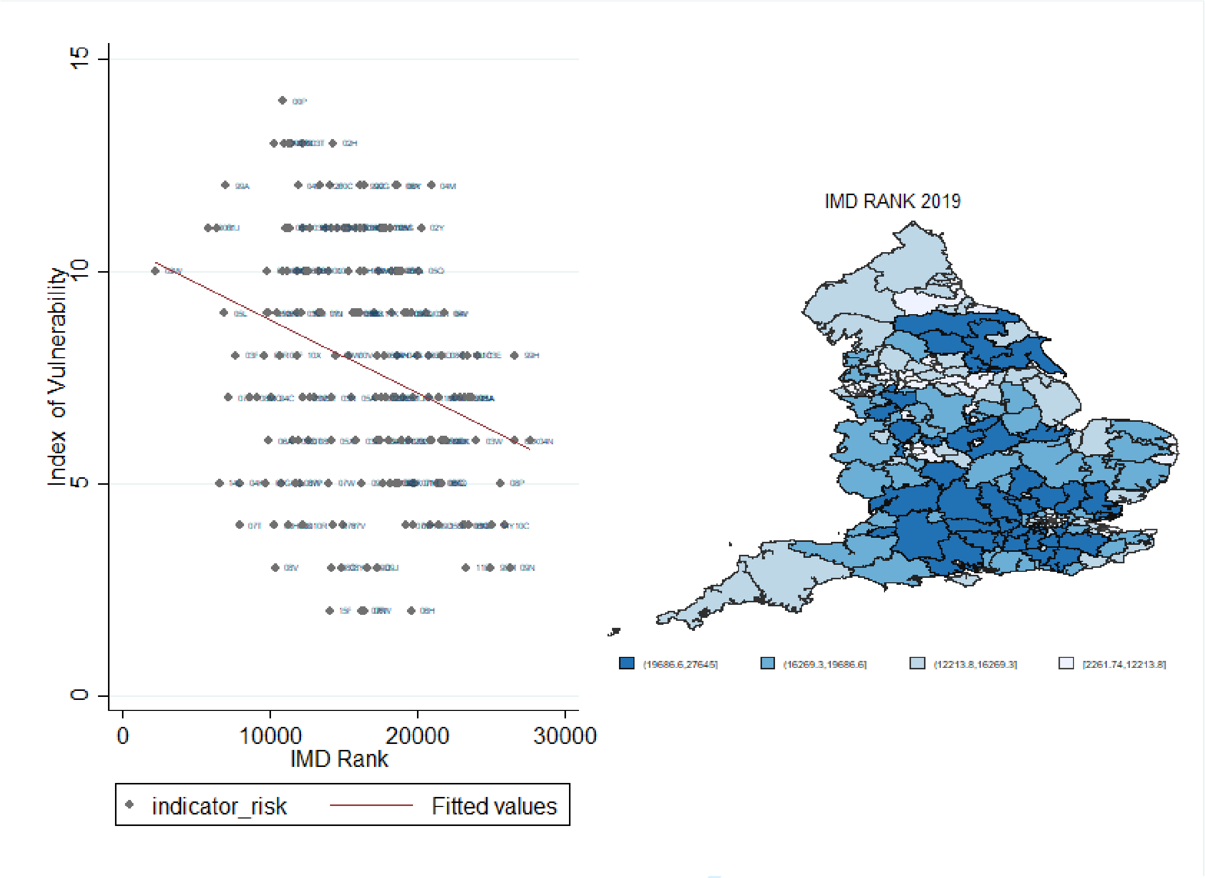

Source: Own elaboration based on NHS digital data at CCGs areas 2019.

Figure 4: **Correlation of IMD Rank and Index of vulnerability versus mortality rate**

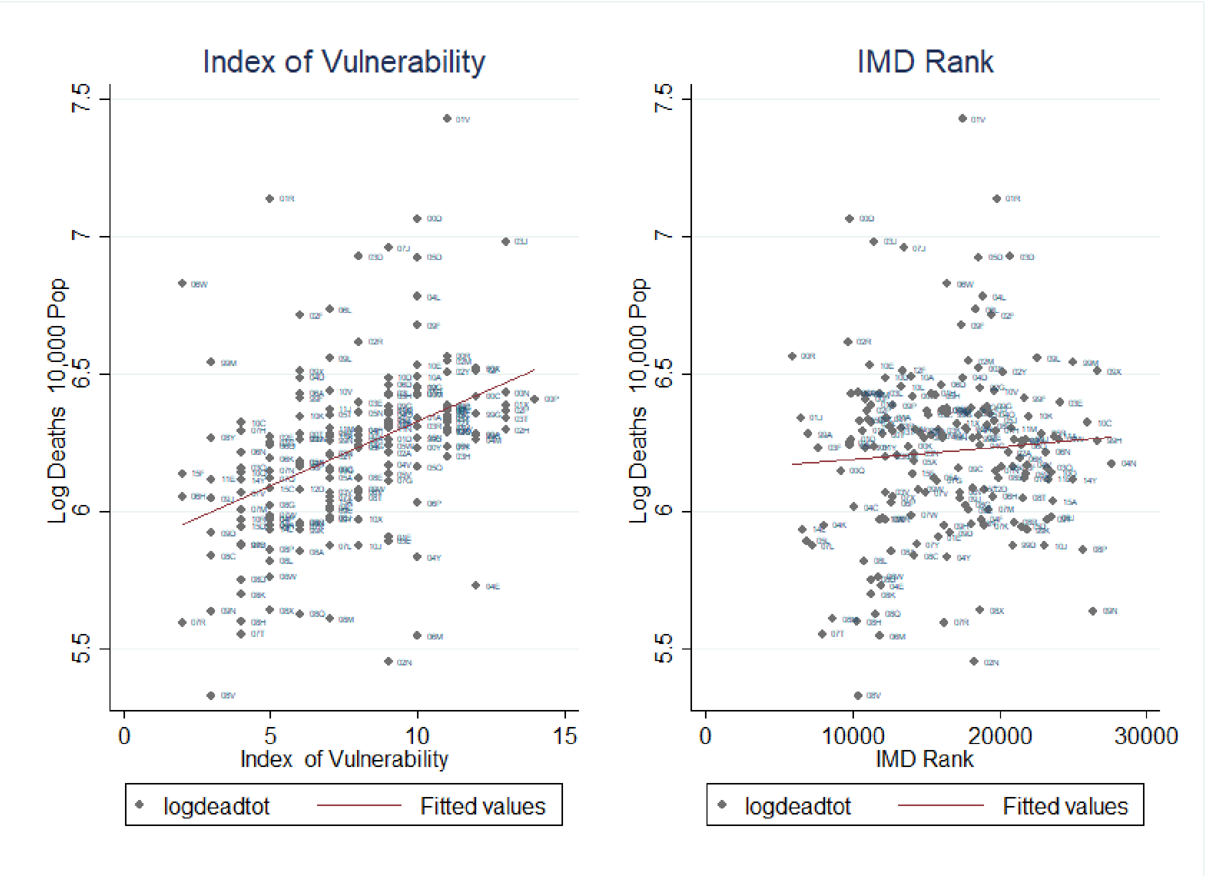

Source: Own elaboration based on NHS digital data at CCGs areas 2019 and mortality rate of the first 22 weeks of the year 2020.

For peer review only - http://bmjopen.bmj.com/site/about/guidelines.xhtml

Supplementary file

Table S1: Descriptive Statistics

	Mean	Sd	Min	Max	# Obs
Nurses per 10,000 population	3.05	0.87	1.15	6.4	191
GPs per 10,000 population	5.98	0.88	3.81	9.26	191
NHS Hospital staff per 10,000 population	191.92	236.18	0	1592.15	191
IMD CCG Rank	16242.3	4927.95	2261.74	27645	191
COPD prevalence per 10,000 patients	201.32	66.28	79.52	406.13	191
Cancer prevalence per 10,000 patients	306.04	83.35	67.83	593.81	191
CKD prevalence per 10,000 patients	336.75	114.19	108.96	814.88	191
Diabetes prevalence per 10,000 patients	569.58	107.99	241.18	1090.75	191
Hypertension prevalence per 10,000 patients	1427.8	241.87	738.36	1857.47	191
Cardiovascular disease prevalence per 10,000 patients	137.8	35.94	56.69	216.38	191
Proportion aged 70+ per 10,000 patients	38706	27289	3845	207788	191
Ratio of residential care homes per 10,000 population aged 70+	21.11	7.15	1.52	44.73	191
Indicator of unplanned admissions for chronic disease	924.97	272.18	170.3	1666.9	191
Indicator of hospital readmission within 30 days	14.22	1.09	11.6	17.6	184
Indicator of mortality within 30 days of discharge	403.6	67.64	211.27	573.09	184
Indicator of acute emergency admission	1427.97	376.18	243.4	2305.8	191
Rural/Urban GGC Indicator	4.17	1.57	1	6	191

For peer review only

Figure S1: Residential homes care and population age distribution at CCGs level

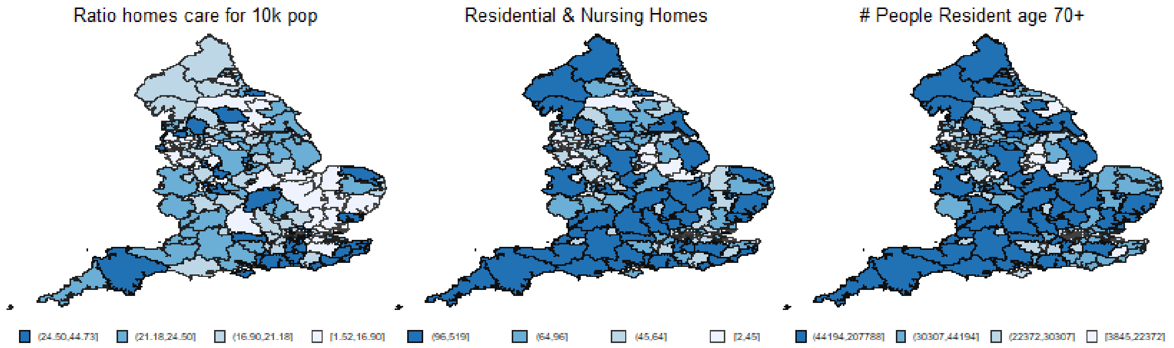

Source: Own elaboration based on NHS digital data at CCGs areas.

Figure S2: Prevalence diseases distribution across CCGs areas

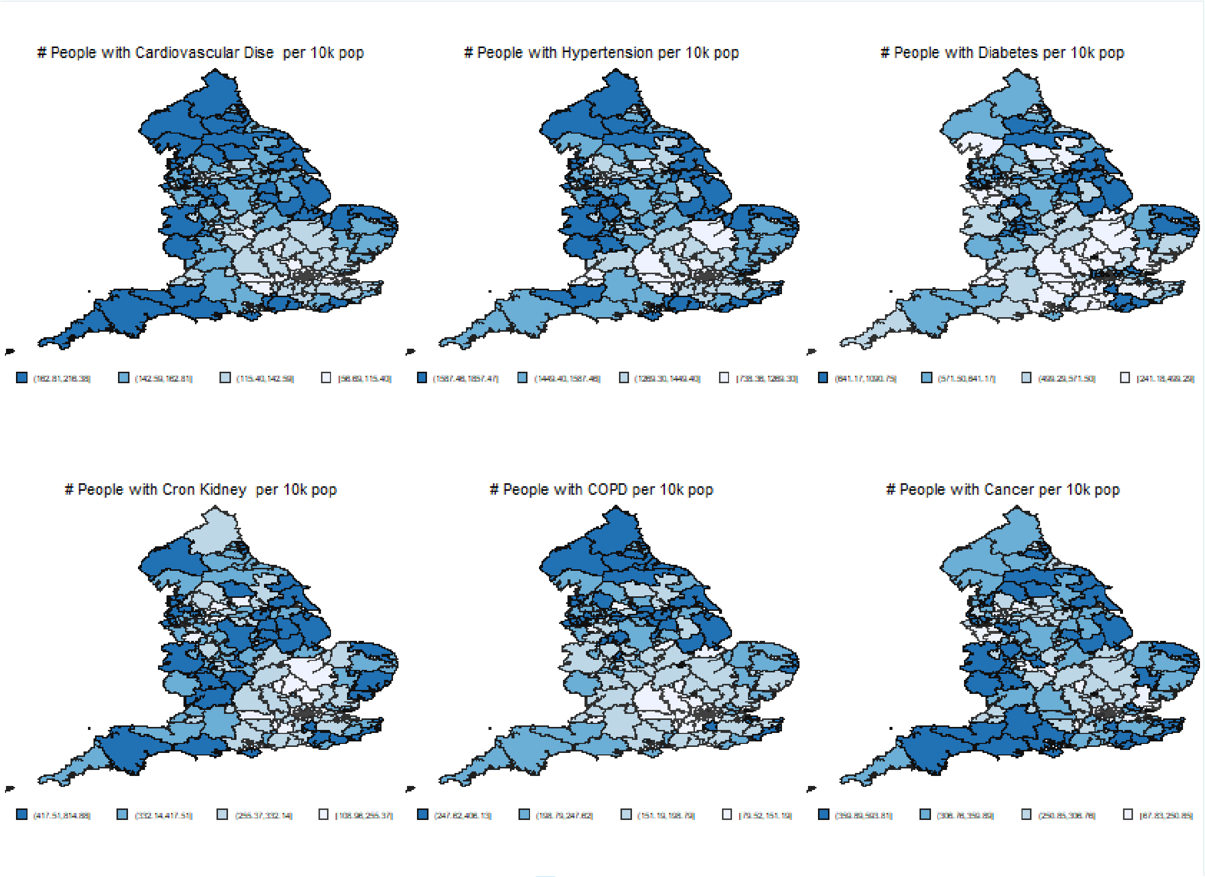

Source: Own elaboration based on NHS digital data at CCGs areas 2019.

Figure S3: Workforce employed by the National Health System at CCGs level

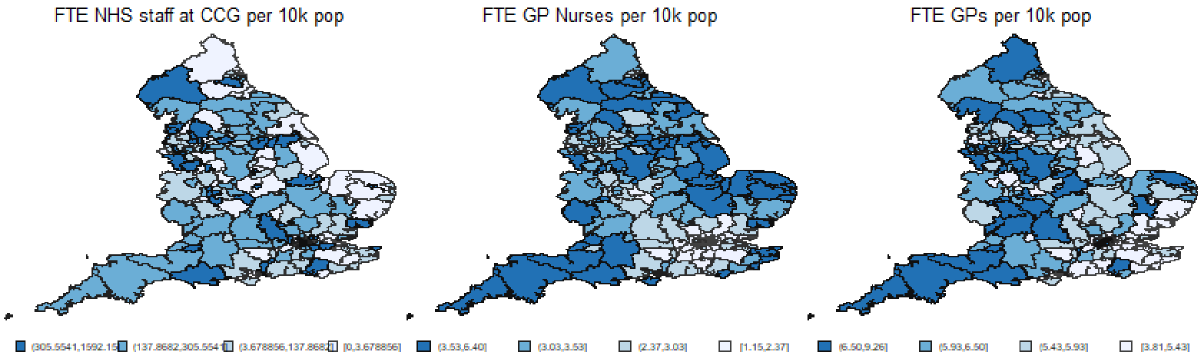

Source: Own elaboration based on NHS digital data at CCGs areas 2019.

Figure S4: Quality of Healthcare Indicators at CCGs level

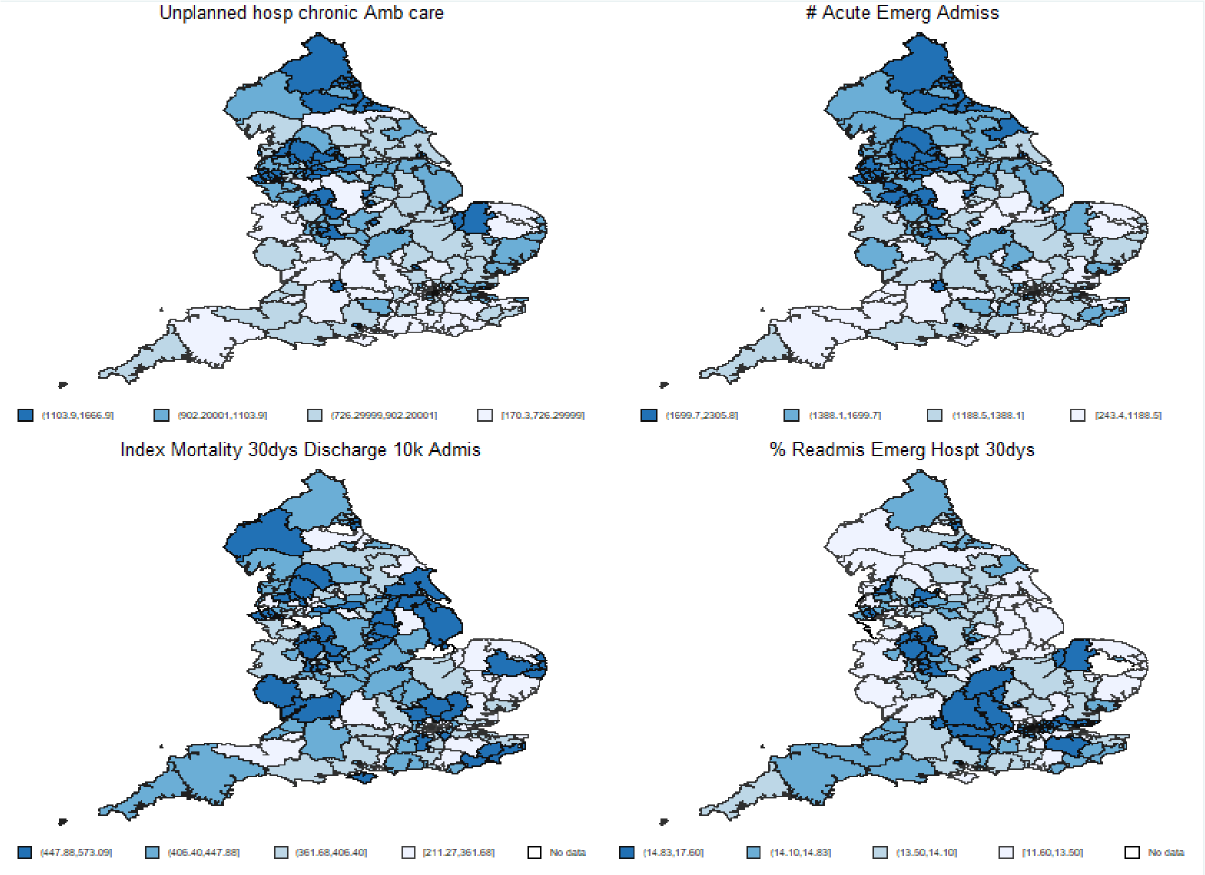

Source: Own elaboration based on NHS digital data at CCGs areas.

For peer review only

Figure S5: **Quartile-based Index of Vulnerability at CCGs level**

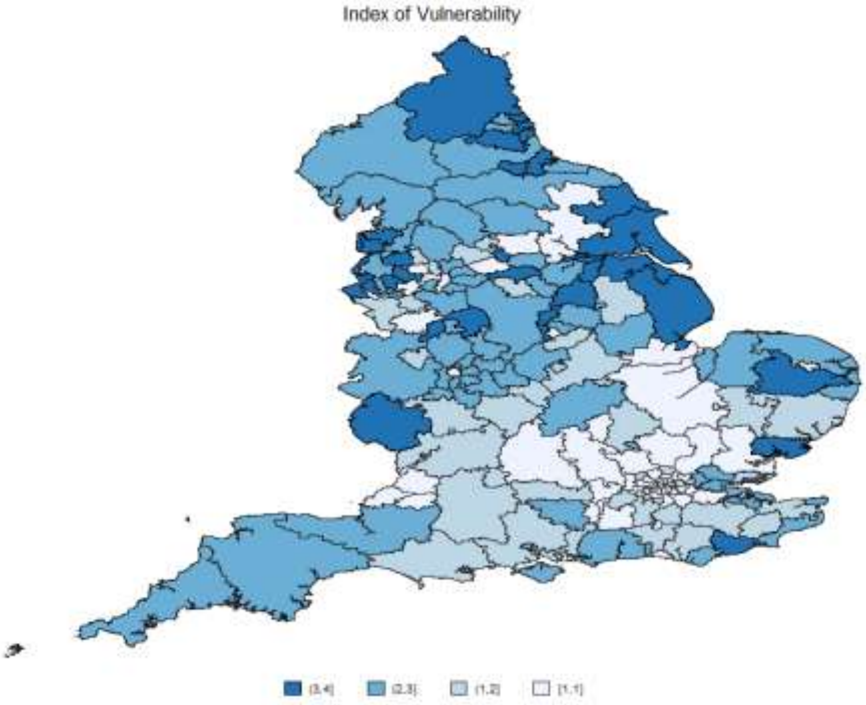

Source: Own elaboration based on NHS digital data at CCGs areas.

Figure S6: **Spatial Cross-Correlation of Index of Vulnerability (left) and IMD (right) with morality**

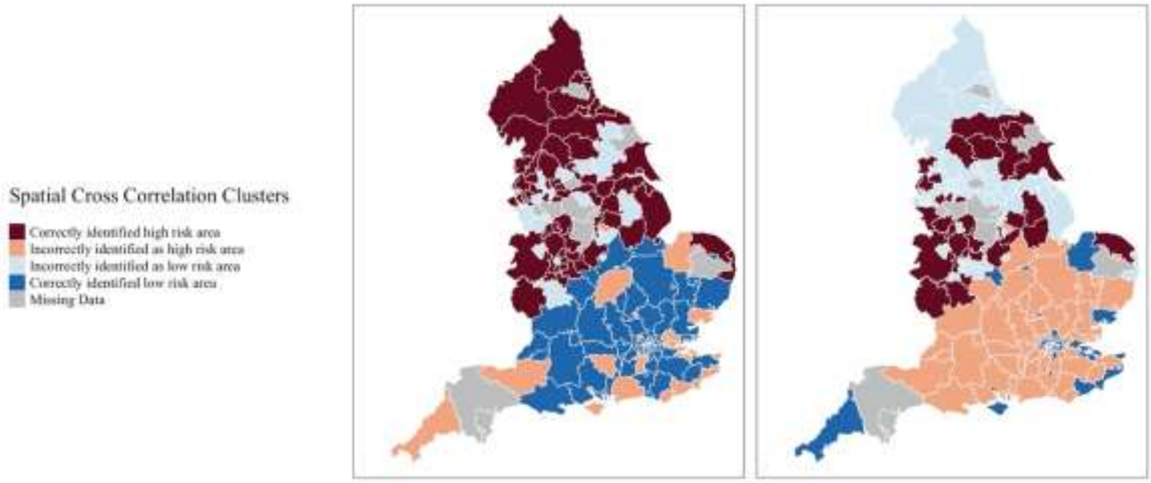

Source: Own elaboration based on NHS digital data at CCGs areas 2019 and mortality rate of the first 22 weeks of the year 2020: computed via the SpatialEco R-package

For peer review only

STROBE Statement—checklist of items that should be included in reports of observational studies

	Item No	Recommendation	Page No
**Title and abstract**	1	(*a*) Indicate the study's design with a commonly used term in the title or the abstract	1
		(*b*) Provide in the abstract an informative and balanced summary of what was done and what was found	2
**Introduction**			
Background/rationale	2	Explain the scientific background and rationale for the investigation being reported	3
Objectives	3	State specific objectives, including any prespecified hypotheses	3
**Methods**			
Study design	4	Present key elements of study design early in the paper	3
Setting	5	Describe the setting, locations, and relevant dates, including periods of recruitment, exposure, follow-up, and data collection	3
Participants	6	(*a*) *Cohort study*—Give the eligibility criteria, and the sources and methods of selection of participants. Describe methods of follow-up<br>*Case-control study*—Give the eligibility criteria, and the sources and methods of case ascertainment and control selection. Give the rationale for the choice of cases and controls<br>*Cross-sectional study*—Give the eligibility criteria, and the sources and methods of selection of participants	3
		(*b*) *Cohort study*—For matched studies, give matching criteria and number of exposed and unexposed<br>*Case-control study*—For matched studies, give matching criteria and the number of controls per case	NA
Variables	7	Clearly define all outcomes, exposures, predictors, potential confounders, and effect modifiers. Give diagnostic criteria, if applicable	3-4
Data sources/ measurement	8*	For each variable of interest, give sources of data and details of methods of assessment (measurement). Describe comparability of assessment methods if there is more than one group	3-4
Bias	9	Describe any efforts to address potential sources of bias	NA
Study size	10	Explain how the study size was arrived at	NA
Quantitative variables	11	Explain how quantitative variables were handled in the analyses. If applicable, describe which groupings were chosen and why	NA
Statistical methods	12	(*a*) Describe all statistical methods, including those used to control for confounding	4-7
		(*b*) Describe any methods used to examine subgroups and interactions	NA
		(*c*) Explain how missing data were addressed	NA
		(*d*) *Cohort study*—If applicable, explain how loss to follow-up was addressed<br>*Case-control study*—If applicable, explain how matching of cases and controls was addressed<br>*Cross-sectional study*—If applicable, describe analytical methods taking account of sampling strategy	NA
		(*e*) Describe any sensitivity analyses	4-5

Continued on next page

For peer review only - http://bmjopen.bmj.com/site/about/guidelines.xhtml
For peer review only

**Results**			
Participants	13*	(a) Report numbers of individuals at each stage of study—eg numbers potentially eligible, examined for eligibility, confirmed eligible, included in the study, completing follow-up, and analysed	NA
		(b) Give reasons for non-participation at each stage	NA
		(c) Consider use of a flow diagram	NA
Descriptive data	14*	(a) Give characteristics of study participants (eg demographic, clinical, social) and information on exposures and potential confounders	12
		(b) Indicate number of participants with missing data for each variable of interest	12
		(c) *Cohort study*—Summarise follow-up time (eg, average and total amount)	NA
Outcome data	15*	*Cohort study*—Report numbers of outcome events or summary measures over time	NA
		Case-control study—Report numbers in each exposure category, or summary measures of exposure	NA
		Cross-sectional study—Report numbers of outcome events or summary measures	NA
Main results	16	(*a*) Give unadjusted estimates and, if applicable, confounder-adjusted estimates and their precision (eg, 95% confidence interval). Make clear which confounders were adjusted for and why they were included	12-13
		(*b*) Report category boundaries when continuous variables were categorized	NA
		(*c*) If relevant, consider translating estimates of relative risk into absolute risk for a meaningful time period	NA
Other analyses	17	Report other analyses done—eg analyses of subgroups and interactions, and sensitivity analyses	4-5
**Discussion**			
Key results	18	Summarise key results with reference to study objectives	10
Limitations	19	Discuss limitations of the study, taking into account sources of potential bias or imprecision. Discuss both direction and magnitude of any potential bias	9-10
Interpretation	20	Give a cautious overall interpretation of results considering objectives, limitations, multiplicity of analyses, results from similar studies, and other relevant evidence	10
Generalisability	21	Discuss the generalisability (external validity) of the study results	10
**Other information**			
Funding	22	Give the source of funding and the role of the funders for the present study and, if applicable, for the original study on which the present article is based	11

*Give information separately for cases and controls in case-control studies and, if applicable, for exposed and unexposed groups in cohort and cross-sectional studies.

Note: An Explanation and Elaboration article discusses each checklist item and gives methodological background and published examples of transparent reporting. The STROBE checklist is best used in conjunction with this article (freely available on the Web sites of PLoS Medicine at http://www.plosmedicine.org/, Annals of Internal Medicine at http://www.annals.org/, and Epidemiology at http://www.epidem.com/). Information on the STROBE Initiative is available at www.strobe-statement.org.

For peer review only - http://bmjopen.bmj.com/site/about/guidelines.xhtml
